# Investigating the Transition of Pre-Symptomatic to Symptomatic Huntington’s Disease Status Based on Omics Data

**DOI:** 10.3390/ijms21197414

**Published:** 2020-10-08

**Authors:** Christiana C. Christodoulou, Margarita Zachariou, Marios Tomazou, Evangelos Karatzas, Christiana A. Demetriou, Eleni Zamba-Papanicolaou, George M. Spyrou

**Affiliations:** 1 Bioinformatics Department; Cyprus Institute of Neurology and Genetics; Cyprus School of Molecular Medicine, 2371 Nicosia, Cyprus; christianachr@cing.ac.cy (C.C.C.); margaritaz@cing.ac.cy (M.Z.); mariost@cing.ac.cy (M.T.); 2 Neurology Clinic D; Cyprus Institute of Neurology and Genetics; Cyprus School of Molecular Medicine, 2371 Nicosia, Cyprus; ezamba@cing.ac.cy; 3 Cyprus School of Molecular Medicine of the Cyprus Institute of Neurology and Genetics, 2371 Nicosia, Cyprus; 4 Department of Informatics and Telecommunications, University of Athens, 157 72 Athens, Greece; vagkaratzas@di.uoa.gr; 5 Department of Primary Care and Population Health, University of Nicosia, 2417 Nicosia, Cyprus; demetriou.chri@unic.ac.cy

**Keywords:** huntington’s disease, systems bioinformatics, pathways, metabolites, gene co-expression, network biology, differentially expressed genes, network rewiring

## Abstract

Huntington’s disease is a rare neurodegenerative disease caused by a cytosine–adenine–guanine (CAG) trinucleotide expansion in the Huntingtin (*HTT*) gene. Although Huntington’s disease (HD) is well studied, the pathophysiological mechanisms, genes and metabolites involved in HD remain poorly understood. Systems bioinformatics can reveal synergistic relationships among different omics levels and enables the integration of biological data. It allows for the overall understanding of biological mechanisms, pathways, genes and metabolites involved in HD. The purpose of this study was to identify the differentially expressed genes (DEGs), pathways and metabolites as well as observe how these biological terms differ between the pre-symptomatic and symptomatic HD stages. A publicly available dataset from the Gene Expression Omnibus (GEO) was analyzed to obtain the DEGs for each HD stage, and gene co-expression networks were obtained for each HD stage. Network rewiring, highlights the nodes that change most their connectivity with their neighbors and infers their possible implication in the transition between different states. The *CACNA1I* gene was the mostly highly rewired node among pre-symptomatic and symptomatic HD network. Furthermore, we identified *AF198444* to be common between the rewired genes and DEGs of symptomatic HD. *CNTN6, DEK, LTN1, MST4, ZFYVE16, CEP135, DCAKD, MAP4K3, NUPL1* and *RBM15* between the DEGs of pre-symptomatic and DEGs of symptomatic HD and *CACNA1I, DNAJB14, EPS8L3, HSDL2, SNRPD3, SOX12, ACLY, ATF2, BAG5, ERBB4, FOCAD, GRAMD1C, LIN7C, MIR22, MTHFR, NABP1, NRG2, OTC, PRAMEF12, SLC30A10, STAG2* and *Y16709* between the rewired genes and DEGs of pre-symptomatic HD. The proteins encoded by these genes are involved in various biological pathways such as phosphatidylinositol-4,5-bisphosphate 3-kinase activity, cAMP response element-binding protein binding, protein tyrosine kinase activity, voltage-gated calcium channel activity, ubiquitin protein ligase activity, adenosine triphosphate (ATP) binding, and protein serine/threonine kinase. Additionally, prominent molecular pathways for each HD stage were then obtained, and metabolites related to each pathway for both disease stages were identified. The transforming growth factor beta (TGF-β) signaling (pre-symptomatic and symptomatic stages of the disease), calcium (Ca^2+^) signaling (pre-symptomatic), dopaminergic synapse pathway (symptomatic HD patients) and Hippo signaling (pre-symptomatic) pathways were identified. The in silico metabolites we identified include Ca^2+^, inositol 1,4,5-trisphosphate, sphingosine 1-phosphate, dopamine, homovanillate and L-tyrosine. The genes, pathways and metabolites identified for each HD stage can provide a better understanding of the mechanisms that become altered in each disease stage. Our results can guide the development of therapies that may target the altered genes and metabolites of the perturbed pathways, leading to an improvement in clinical symptoms and hopefully a delay in the age of onset.

## 1. Introduction

Huntington’s disease (HD), which was first described in 1872 by Dr. George Huntington [1], is a rare, progressive and devastating neurodegenerative disease with autosomal dominant inheritance [2]. The medium spiny neurons of the basal ganglia of the central nervous system play a role in executive function, behavior and motor control as well as undergo neuronal degeneration [3]. 

HD is caused by a CAG trinucleotide repeat on the huntingtin (*HTT*) gene that is located on exon 1 of chromosome 4. The huntingtin protein (HTT) is encoded by the *HTT* gene [4]. The *HTT* gene is located in a repeated DNA fragment that consists of cytosine-adenine-guanine (CAG) which is repeated multiple times repeat [4]. 

The number of CAG repeats is the main predictor for age of onset and disease severity in HD [3]. In healthy individuals, the CAG trinucleotide is repeated normally between 10–35 times [3]. Individuals that have between 36–39 CAG repeats may or may not develop HD, meaning that there is reduced penetrance. However, individuals with 40 or more CAG repeats will always develop the signs and symptoms of HD [3].

The typical age of onset for HD is approximately 40 years, and the average life expectancy is 17 years after symptom onset [5,6]. Clinical characteristics include: (i) movement impairment such as chorea, (ii) an involuntary twitching movement and incoordination, (iii) cognitive impairment such as lapse in short-term memory and (iv) behavioral impairment such as depression, personality changes and psychosis. As the disease progresses, the involuntary movements become more prominent [5]. Clinical characteristics of HD patients are evaluated using the Unified Huntington’s Disease Rating Scale (UHDRS), which evaluates (i) motor function, (ii) cognition, (iii) behavior and (iv) functional abilities [5].

HD is a monogenetic and incurable disease and at the same time its molecular manifestations remain highly complex and involve multiple cellular processes, genes, and metabolites, which needs to be investigated to understand HD pathology. Systems bioinformatics (SB) allows the integration of different biological omics data to better understand the biological pathways, mechanisms, genes and metabolites involved in HD and lead to possible therapeutic treatments and biomarker discovery. 

SB is an interdisciplinary field which combines the research fields of systems biology and bioinformatics. SB allows the integration of biological data across the omics categories such a genomics, transcriptomics, proteomics, metabolomics, lipidomics, epigenomics and several types of omics data [7]. 

A major approach in this direction is the generation and construction of biological networks representing each level of omics data and their integration in a layered network that permits the exchange of information between and within the layers. The goal is to reveal synergistic relationships among numerous factors rather than explore each entity individually. This data integration approach results in the construction of highly complex molecular interaction networks. The biological data, obtained through large-scale omics analysis can provide a better understanding into biological mechanisms and pathways and how a dysfunction in these mechanisms and pathways can cause the disease [7]. Furthermore, the emerging importance of biological network-based approaches, allows for potential biological and clinical applications by suggesting an intuitive and trustworthy approach to explore the biological and molecular complexity of a disease of interest [8]. 

The metabolome is defined as the complete set of small chemical molecules found within a biological samples (urine, cerebrospinal fluid (CSF), serum, plasma), tissues and cells. Changes and interactions in gene and protein expression and the environment are directly revealed in the metabolome making it more chemically and physically complex than the genome, transcriptome and proteome. Metabolites are affected by the upstream influence of the genome, proteome, environmental and lifestyle factors, as well as medication and underlying diseases [9].

Metabolomics is an omics category focused in the study of metabolites. Metabolites are defined as small biological and low molecular weight (<1500 Da) compounds, they are the end-products of metabolism [10]. There are two categories of metabolites, the primary metabolites which are directly involved in the growth, reproduction and development of the cell these include amino acids, sugars and lipids and the secondary metabolites which are indirectly involved in the growth, reproduction and development of the cell such as drugs. The investigation of metabolites allows the identification of metabolic pathways that become activated or dysfunctional in patients. Identification of such disease specific metabolites can eventually result in HD biomarkers [10]. 

The purpose of this work is to shed light on the DEGs between pre-symptomatic (before the onset of symptoms) and symptomatic HD patients and to identify the biological pathways and metabolites which appear to be altered between the pre-symptomatic and symptomatic stages. The pathways and metabolites that are present or aberrant in each of the two disease stages provide further insight into HD phenoconversion and into how these elements become dysfunctional, contributing to disease onset and severity.

The aim of our work is to identify the genes, biological pathways and metabolites for pre-symptomatic and symptomatic HD patients and observe how alterations in genes, pathways and metabolites change as HD patients progress from the pre-symptomatic to the symptomatic stage of the disease. Furthermore, our work provides insight towards the development of therapeutics, aiming to delay the age of onset and potentially help decrease disease severity and symptoms. 

## 2. Results

### 2.1. Differentially Expressed Genes in Pre-Symptomatic and Symptomatic HD Patients

The top 150 over and top 150 under expressed genes, therefore a total of 300 genes were identified respectively for each group of HD patients. The DEGs for the pre-symptomatic and symptomatic HD patients, in comparison to controls, are shown in Table S1 and Table S2 respectively. DEGs that are highlighted through our pipeline and have also been already associated with HD based on the bibliography, are highlighted in Table S3. Specifically, we annotate their role in pathophysiological mechanisms such as neuro-inflammation, apoptosis, anti-oxidants and Ca^2+^ dysregulation that are involved in HD. 

### 2.2. Gene Co-Expression Networks of Pre-Symptomatic and Symptomatic HD Patients

**Figure 1:**
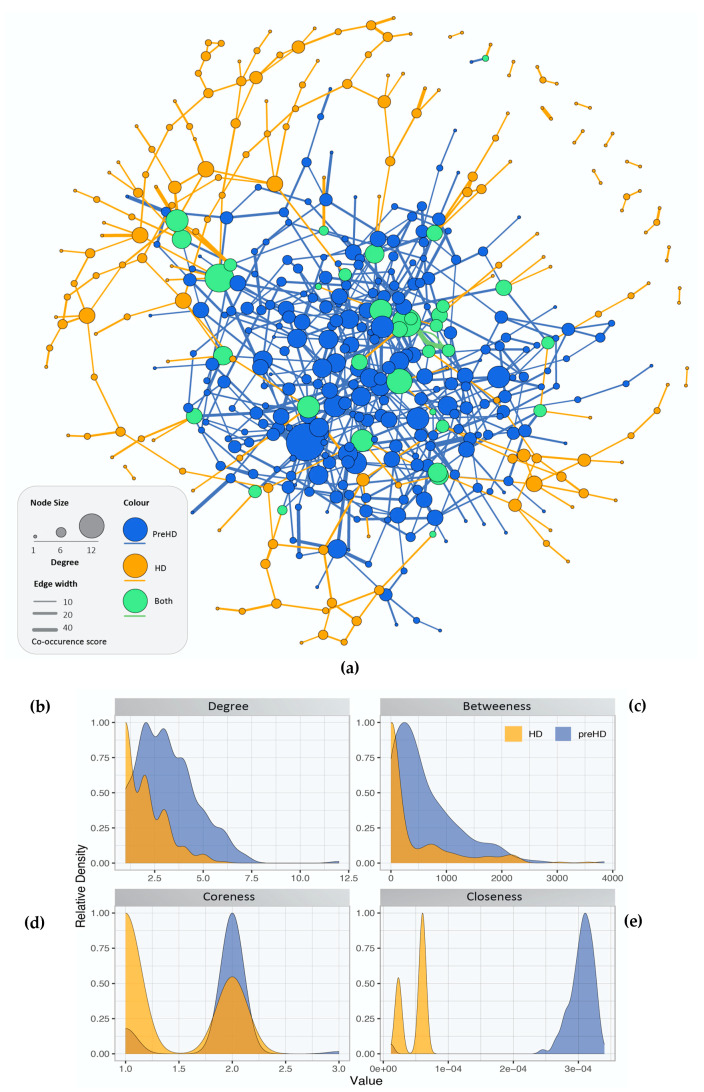
Network topological analysis of the gene co-expression. (**a**) Gene co-expression networks for controls versus pre-symptomatic and controls versus symptomatic HD. Blue nodes represent: the genes involved in pre-symptomatic HD, orange nodes represent: the genes involved in the symptomatic HD stage and green nodes represent the genes which appear in both HD networks. Edge colour represents co-expression in the respective groups (either or both HD stages) while edge thickness represents co-occurrence score (**b**–**e**) Distribution of the calculated centralities for the pre-symptomatic and symptomatic HD networks, i.e., (**b**) Degree (**c**) Betweenness (**d**) Coreness and (**e**) Closeness.

The gene co-expression networks containing the DEGs for controls versus pre-symptomatic and versus symptomatic HD patients are illustrated in (Figure 1). Blue nodes represent the genes present in pre-symptomatic HD patients, orange nodes represent genes present in symptomatic HD patients and green nodes represent genes found in both the pre-symptomatic and symptomatic HD patients. Similarly, blue, orange and green edge colour represent co-expressions observed in pre-symptomatic, symptomatic and both HD stages respectively. Additionally, network topology analysis revealed a more tightly connected co-expression network for the pre-symptomatic HD patients compared to the symptomatic, as shown by the differences in the degree, betweenness, coreness and closeness distributions in Figure 1b–e.

### 2.3. Network Rewiring between Gene Co-Expression Networks of Pre-Symptomatic and Symptomatic HD Patients Using DyNet 

**Figure 2:**
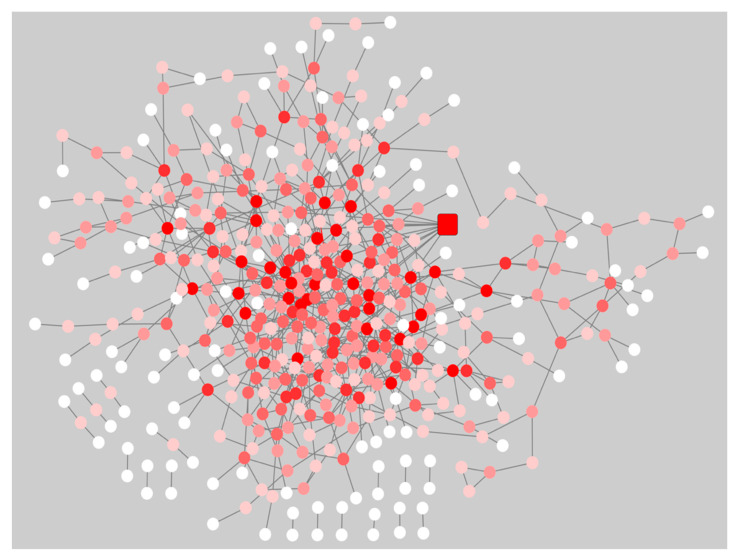
Central reference network of the pre-symptomatic and symptomatic HD network using the Cytoscape plug-in DyNet. Dark red nodes: Most highly re-wired nodes, Medium red: Highly re-wired nodes, Light red: Least most re-wired nodes and White nodes: No re-wiring. The square node indicates the *CACNA1I* gene, which was the most highly re-wired node based on the DyNet re-wiring score.

The gene co-expression networks for the pre-symptomatic and symptomatic HD networks were used to identify the most re-wired nodes between the pre-symptomatic and symptomatic HD networks using the DyNet Cytoscape plug-in [11]. The central reference network is an overlapping visualization view consisting of the pre-symptomatic and symptomatic HD networks as illustrated in (Figure 2). The most highly re-wired node identified based on the D_n_ (DyNet re-wiring) score as seen in (Figure 2) and drawn as a square node, was the calcium voltage-gated channel subunit alpha 1 I (*CACNA1I)*. In our data we identified the *CACANA1I* gene to be significant with a *p*-value of 1.96E-05 and logFC 2.744 in pre-symptomatic HD patients. The *CACNA1I* gene encodes the protein CACNA1I, which is a member of a sub-family of Ca^2+^ channels. The following voltage-gated Ca^2+^ channel is involved in Ca^2+^ signalling in neurons [https://www.genecards.org/], [12], and Supplementary Table S4 shows the presence or absence of genes indicated by true or false in the pre-symptomatic and symptomatic networks.

**Figure 3:**
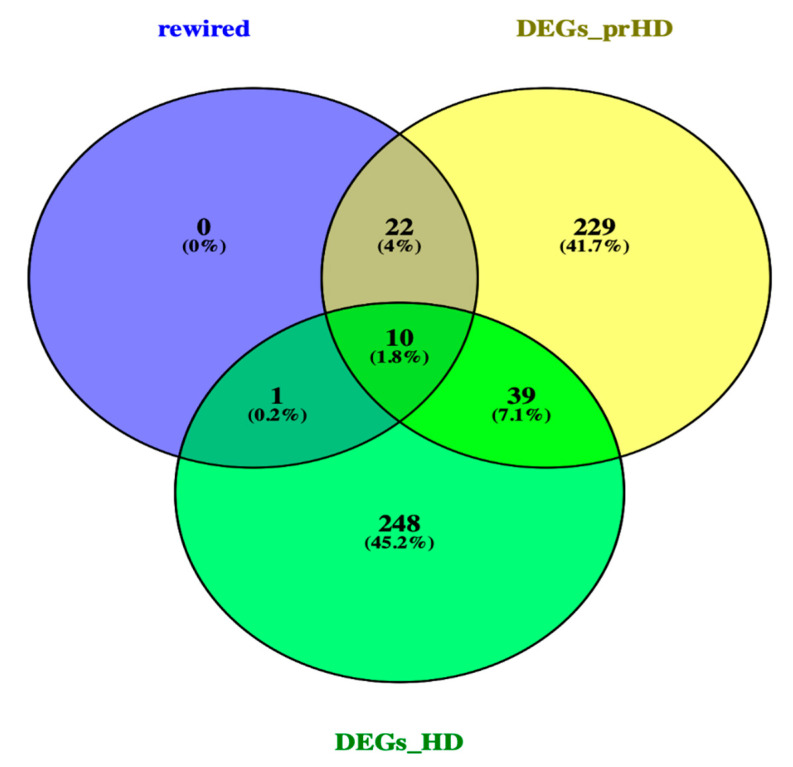
Venn diagram of rewired genes and DEGs of pre-symptomatic and symptomatic HD. Venn diagram illustrates the number of common genes between the rewired genes and DEGs of the two HD stages.

A Venn diagram [https://bioinfogp.cnb.csic.es/tools/venny/], was used to identify, the DEGs and rewired genes common between the pre-symptomatic and symptomatic HD stages. The Venn Figure 3, identified, the *AF198444* gene to be common between the rewired genes and DEGs symptomatic HD. Ten common genes such as *CNTN6, DEK, LTN1, MST4, ZFYVE16, CEP135, DCAKD, MAP4K3, NUPL1* and *RBM15* were identified between the DEGs for pre-symptomatic and DEGs for symptomatic HD. Some of the biological functions identified for the above-mentioned genes, include ubiquitin protein ligase activity, ATP binding, adenyl ribonucleotide, protein serine/threonine kinase activity, purine ribonucleotide triphosphate binding, ubiquitin-protein transferase activity, protein kinase activity and RNA binding (Table S5). Additionally, twenty-two common genes such as *CACNA1I, DNAJB14, EPS8L3, HSDL2, SNRPD3, SOX12, ACLY, ATF2, BAG5, ERBB4, FOCAD, GRAMD1C, LIN7C, MIR22, MTHFR, NABP1, NRG2, OTC, PRAMEF12, SLC30A10, STAG2 and Y16709* were identified between the rewired and DEGs of pre-symptomatic. Some of the biological pathways identified include, phosphatidylinositol-4,5-bisphosphate 3-kinase activity, phosphatidylinositol bisphosphate kinase activity, cyclic adenosine monophosphate (cAMP) response element binding protein binding, protein tyrosine kinase activity, voltage-gated ion channel activity involved in regulation of postsynaptic membrane potential, voltage-gated calcium channel activity, manganese ion transmembrane transporter activity and numerous additional pathways in Table S6. Furthermore, from our studied we identified the genes of *SP3* and *PCNP* from the DEGs symptomatic HD and *CAPZA1* from the DEGs pre-symptomatic HD. The following three genes, were previous identified by [13] as biomarkers for HD.

The DEGs and re-wired genes obtained from DyNet were separately used as input for enrichment analysis in the EnrichR web-tool [14] for pre-symptomatic and symptomatic HD cases as well as the rewired network, to identify the common enriched pathways and genes among the co-expression networks and the re-wired HD network. The common pathways between the pre-symptomatic, symptomatic HD networks and the re-wired network were selected based on the top ranking score.

Some of the common pathways identified between the pre-symptomatic and the re-wired network include: glycosaminoglycan degradation, citrate acid cycle, ErbB signaling pathway, adherens junctions, Ca^2+^ signalling and arginine biosynthesis. Common pathways identified between the symptomatic and the rewired network include the endocytosis and proteasome pathways. 

### 2.4. PathwayConnector Clustering of Pathways Identifed for Pre-Symptomatic and Symptomatic HD Patients 

We further identified the HD- related pathways for the pre-symptomatic and symptomatic HD stages using PathwayConnector [15]. The pathways for each stage are shown in Table S7 and Table S8 respectively. 

After enrichment analysis, mapping onto the Kyoto Encyclopedia of Genes and Genomes (KEEG) reference network and the construction of the complementary pathway-to-pathway networks for the pre-symptomatic and symptomatic HD stages, clustering was then implemented to group the final set of HD-related pathways into clusters. 

**Figure 4:**
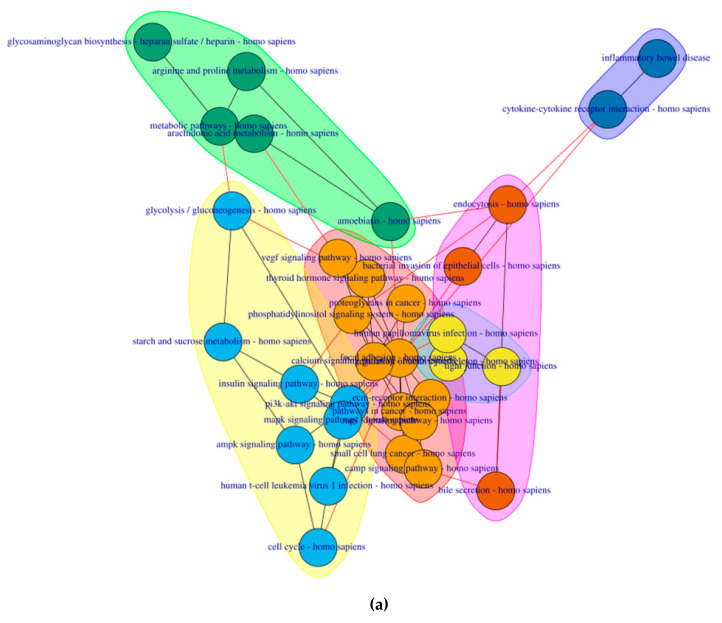

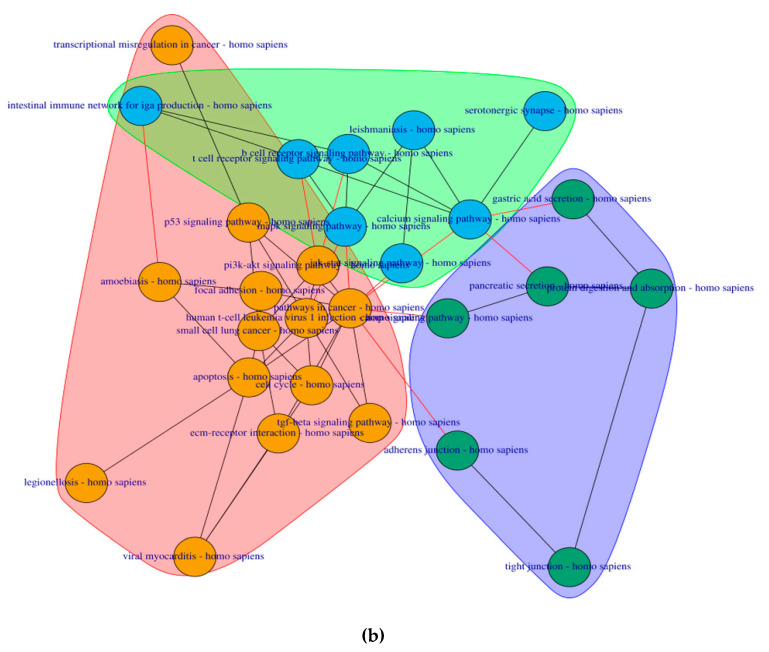
Cluster of connected pathways for pre-symptomatic and symptomatic HD using PathwayConnector (**a**) Clusters of pathways in the pre-symptomatic HD stage. There is a total of six clusters, each shaded in a different color. (**b**) Three clusters of pathways in the symptomatic HD stage.

6 clusters were recognized for the pre-symptomatic HD stage and 3 clusters for the symptomatic HD stage, clustering of pathways is based on the network property of edge-betweenness which defined as the number of shortest paths which go through an edge in a network [16]. The clusters are indicated by the different coloured circles and backgrounds for the pre-symptomatic and symptomatic stages respectively Figure 4. 

### 2.5. GeneTrail3 for the Identification of Pathways by Analysing the DEGs for Pre-Symptomatic and Symptomatic HD Patients

The pathways identified by GeneTrial3 for pre-symptomatic and symptomatic HD from each selected biological database are sorted based on the adjusted *p*-value [17]. The top-15 pathways for pre-symptomatic and symptomatic HD are shown in Table 1 and Table 2 respectively. 

### 2.6. PathWalks for the Analysis of Over and Under Expressed Genes for Pre-Symptomatic and Symptomatic HD Patients 

The results obtained from PathWalks [18] for each HD stage include (i) a list of ranked pathways (which are the most visited by the random walker) and (ii) a list of ranked edges (the most frequently traversed edges). The score indicates the times a respective pathway or edge was accessed by the random walker. 

Odds ratio (OR) analysis was performed for both HD patient groups and for each individual HD patient group. A pathway with an OR of one and above is considered to be significant as the walker did not pass by the pathway by random or by chance whereas a pathway with an OR of below 1, the pathway is not considered to be significant as illustrated in Table S9, Table S10 and Table S11 respectively. 

**Figure 5:**
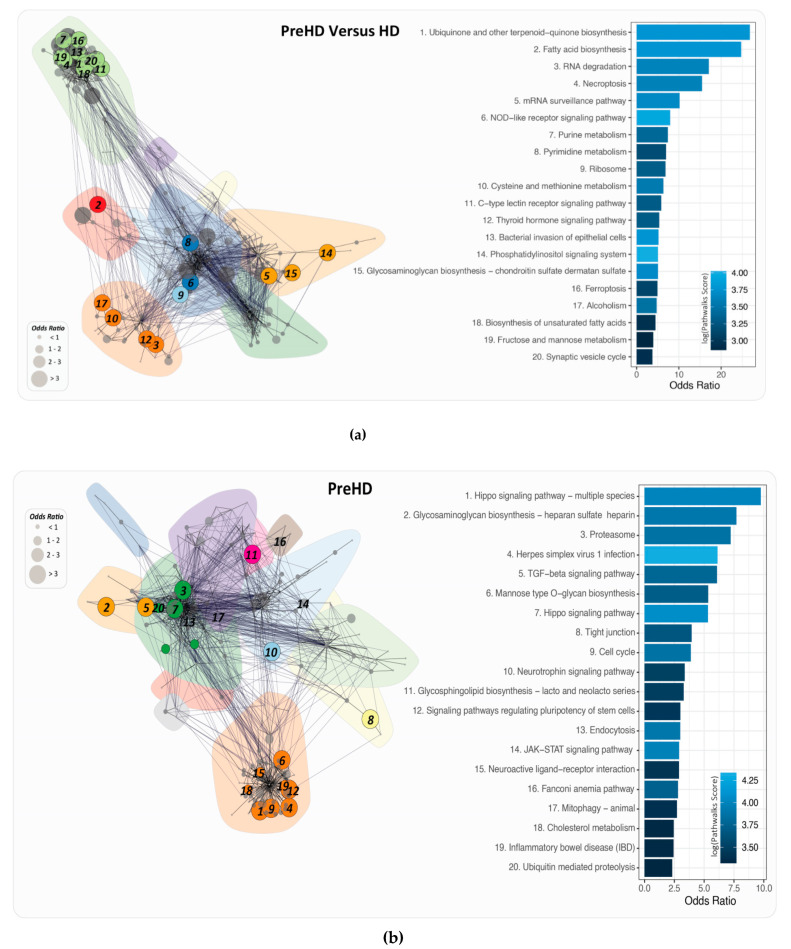

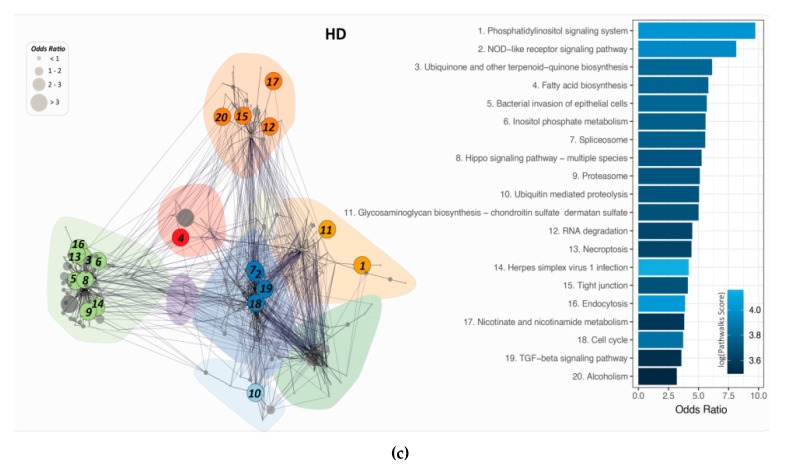
PathWalks derived pathway to pathway networks and odds ratio analysis for (**a**) pre-symptomatic versus symptomatic HD, (**b**) pre-symptomatic HD, (**c**) symptomatic HD. In each network the node size represents the odds ratio (OR) score in 4 bins. The top 20 pathways w.r.t to OR are shown in colour, while the remaining nodes are shown in grey. Edges represent walker transitions between pathways. Colour shading shows the identified communities of highly connected pathways w.r.t to PathWalks scores.

The top pathways obtained from the odd ratio analysis can be seen in Figure 5 for pre-symptomatic and symptomatic HD patients respectively. 

### 2.7. Metabolites Identified and Related to HD Using KEGG

Metabolites are the intermediate end-products of metabolism, using KEGG compound [19] we identified the metabolites relevant to each pathway for the pre-symptomatic and symptomatic HD stages. Following enrichment analysis for the identification of pathways related to pre-symptomatic and symptomatic HD using PathwayConnector [15] the Venny tool [https://bioinfogp.cnb.csic.es/tools/venny/] was used to identify the pathways that were exclusive in the pre-symptomatic and symptomatic HD stages. A total of 40 and 37 exclusive pathways were identified for the pre-symptomatic and symptomatic HD stages, respectively. 

KEGG was utilized for the identification of metabolites for each exclusive pathway in the pre-symptomatic and symptomatic HD stages. The exclusive pathways and the number of metabolites identified for each pathway are shown in Tables S12 and S13. 

Venny [https://bioinfogp.cnb.csic.es/tools/venny/] was used to identify the metabolites which are exclusive in the pre-symptomatic and symptomatic HD stages. A total of 709 and 502 metabolites were identified exclusively for pre-symptomatic and symptomatic HD respectively, and 87 common shared metabolites were identified between the two stages as illustrated in Figure 5. Some of the common metabolites identified include pyruvate, Ca^2+^, glucose 6-phosphate, K+, Na+, glutamate, prostaglandin and several additional metabolites. 

Some of the exclusive metabolites are ascorbic acid, glycine, retinol, succinic acid and 2-hydroxyestradiol and various additional metabolites are exclusive in pre-symptomatic HD whereas, long- chain fatty acids such as arachidonic acid, fructose, quinolinate, eicosonoic acid and several additional metabolites are exclusive in symptomatic HD. Tables S14 and S15 illustrates the exclusive metabolites for each HD stage and Tables S16 and S17 illustrates the exclusive pathways and their respective metabolites for pre-symptomatic and symptomatic HD respectively and Table S18 illustrates the common pathways between the multi-source integration approach and the pre-symptomatic and symptomatic HD. 

**Figure 6:**
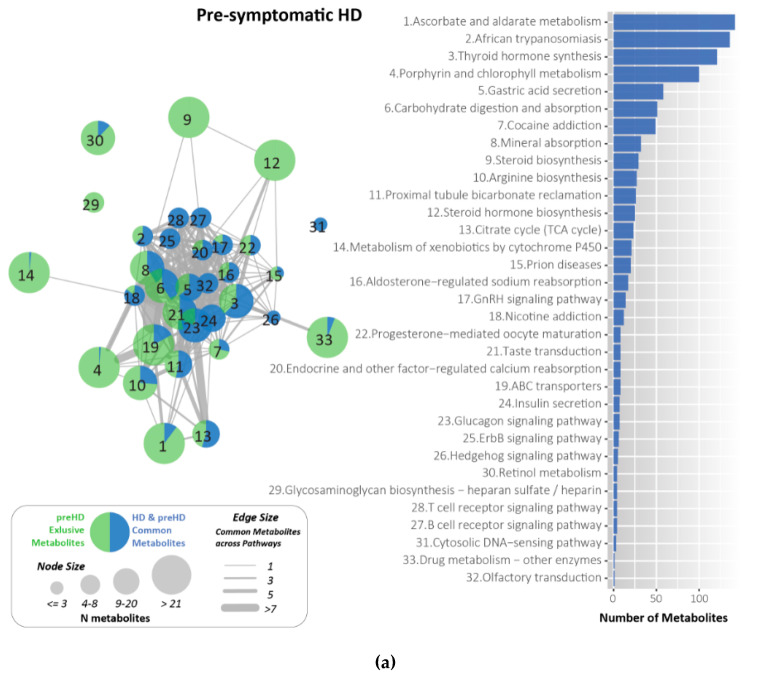

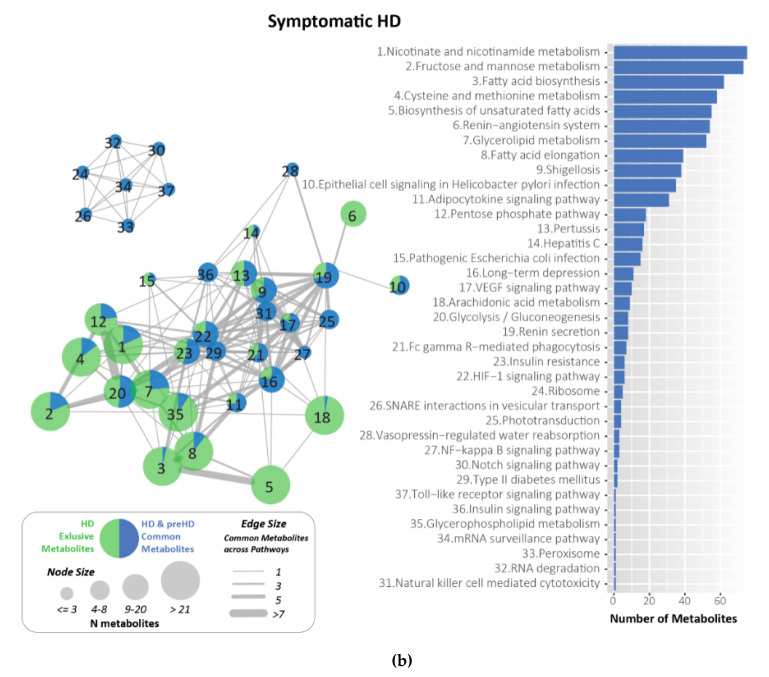
Pathway-metabolite network with pathways and the common and exclusive metabolites for pre-symptomatic and symptomatic HD (**a**) Pathways and common and exclusive metabolites for pre-symptomatic HD (**b**) Pathways and exclusive metabolites for symptomatic HD. The top pathways are shown in colour. Green nodes represent the number of exclusive metabolites for either the pre-symptomatic or symptomatic HD stage. Blue nodes represent the common metabolites in both HD stages. The node size represents the number of common metabolites and smaller nodes represent; the smaller the number of metabolites and larger nodes, the greater the number of metabolites. Edge width represents the number of common metabolites across the pathways.

The common metabolites that are shared between the two stages were excluded, as we aimed to identify exclusive-only metabolites specific for each HD stage. These exclusive metabolites could potentially be (i) important HD drug targets or (ii) biomarkers to distinguish the two stages. Therefore, the exclusive metabolites present for each HD stage were then used as input into Cytoscape to construct a new pathway-metabolite network with pathways and the exclusive metabolites only as illustrated in (Figure 6). 

## 3. Discussion

While HD is a progressive, autosomal dominant inherited disease triggered by CAG repeat expansion in the *HTT* gene, the molecular mechanisms and the pathology of HD remains highly complex and consisting of multiple biological and cellular processes and mechanisms [5].

Through the utilization of multiple bioinformatics tools such as PathwayConnector [15], GeneTrail3 [17] and PathWalks [18] we were able to highlight both pathways and communities of pathways (based on ranked edges in the pathway-pathway network) in both pre-symptomatic and symptomatic HD patients.

Some of the HD related pathways were consistent across the different tools utilized in this work.

The TGF-β pathway was identified to be present in both the pre-symptomatic HD and symptomatic HD. The above-mentioned pathway was identified by PathwayConnector, GeneTrail3 and PathWalks tools. The TGF family of genes includes a group of polypeptides that are structurally and functionally related, the TGF family includes TGF-β1, TGF-β2, TGF-β3 and bone morphogenetic proteins (BMPs) [17]. TGF-βs have a number of biological roles within the cell, such as cell proliferation, differentiation, cell migration, cell survival and apoptosis [17]. TGF-βs, are involved in both physiological and pathological processes within the Central Nervous System (CNS). The up-regulated TGF-β1 has been associated with CNS injury and neurodegeneration [17]. Dysregulation of TGF-β signaling is possibly involved in the HD pathology [18].

A previous study, identified a decrease in the amount of circulating TGF-β1 in pre-symptomatic HD patients while the levels of TGF-β1 in symptomatic HD patients was found to be increased. Furthermore, the levels of TGF-β1 in cortical neurons was identified to be decreased in post-mortem brain tissue from both pre-symptomatic and symptomatic HD patients and in HD mouse models [20]. The same study, investigated that SMAD7, an antagonist of TGF-β signaling, is significantly reduced in striatal cell lines expressing mHTT and in iPSC derived neural progenitors cells (NPCs), an event that may possibly explain the increase in TGF-β signaling in HD neurons [20]. However, further studies are required to clarify the molecular link between the TGF-β family signaling pathways and HD pathology.

The Ca^2+^ signaling pathway was identified for the pre-symptomatic HD patients and for the re-wired network. The Ca^2+^ signaling pathway was identified by both PathwayConnector and PathWalks. Additionally, based on the DyNet re-wiring score, the *CACNA1I* gene seems to play a role in the early stages of HD. A dysregulation in *CACNA1I* may affect Ca^2+^ signaling in neuronal cells. The *CACNA1I* gene encodes for the pore forming subunit of the Ca^2+^ voltage gated channel. The expression profile of CACNA1I protein, includes the brain, thyroid, spleen, small intestine and adrenal gland. The CACNA1I protein, is highly expressed in brain regions of the cerebral cortex, cerebellum, cerebellar hemisphere and Brodmann area 9 and also a high expression in the kidneys. No expression was identified for the thyroid and spleen and a low expression of CACNA1I was identified in the small intestine. The following protein is member of the sub-family of Ca^2+^ channels [12,21]. The CACNA1I channel is characterized by a lower activation and inactivation in comparison to other Ca^2+^ channels. Voltage gated Ca^2+^ channels are located in the membrane of most excitable cells and allow Ca^2+^ influx in response to depolarization, they play a role in regulating intracellular processes such as secretion, neurotransmission and gene expression. Alterations in the CACNA1I, have been associated with autism, schizophrenia epilepsy [12,21]. In neurodegeneration, alternations and dysregulation of Ca^2+^ signaling and homeostasis have been implicated Alzheimer’s disease (AD), Parkinson’s disease (PD) and HD. However, there are to no studies investigating the effect of *CACNA1I* on various neurodegenerative diseases, including HD. Further research in required to investigate the role of *CACNA1I* not only in neurodegenerative diseases but also in HD.Ca^2+^ dysregulation is known to be one of the pathophysiological mechanisms involved in HD. 

Some of the metabolites identified through KEGG [19] include, Ca^2+^, inositol 1,4,5-trisphosphate, sphingosine 1-phosphate and several additional metabolites. 

Ca^2+^ signalling pathway is involved in numerous physiological roles within the cell such as neuronal transmission, neurogenesis, synaptic plasticity and muscle contraction [21]. However, dysregulation of Ca^2+^ has been associated with other neurodegenerative diseases such as AD and PD, and it is believed that Ca^2+^ dysregulation is one of the contributing pathophysiological mechanisms for HD [22]. A number of studies, using HD animal models demonstrated that disruptions of Ca^2+^ signalling, result in alterations in the buffering capacity of Ca^2+^ binding proteins, in abnormal function of Ca^2+^ channels mainly those involved in glutamate excitotoxicity and in disruption of the mitochondrial Ca^2+^ handling system [22,23]. Ca^2+^ disruption is caused by the direct interaction of mHTT with calcium-binding proteins (CBP) such as parvalbumin, calmodulin, calbindin, hypocalcin, ryanodine receptor type 1, inositol trisphosphate receptor (InsP3R1) and different subunits of voltage-gated calcium channels (VGCCs) which result to an increase in the Ca^2+^ concentration and dysfunction of CBP proteins [22].

Furthermore, dysregulation of the Ca^2+^ signaling observed in HD, occurs at the transcriptional level as the mHTT fragments alter the expression of genes involved in Ca^2+^ homeostasis in both HD patients and animal models. Genomics studies conducted in HD animal models revealed a significant difference in the mRNA levels of genes encoding for proteins involved in the intracellular Ca^2+^ regulation and CBPs mentioned above [22]. 

The DEGS and re-wired genes are common between the pre-symptomatic and symptomatic HD stages. The *AF198444* is a long non-coding RNA and it is involved in cisplatin resistance. Cisplatin is given as a chemotherapy medication to treat numerous cancers such as ovarian, cervical, breast, bladder, head, neck and lung cancer [https://go.drugbank.com]. This gene, was identified between re-wired and DEGs symptomatic HD. *CNTN6* is an anchored glycosylphosphatidylinositol neuronal membrane protein, it may possibly be involved in the formation and development of axonal connections in the developing nervous system [12]. *ZFYVE16,* encodes for the endosomal protein that is part of the FYVE zinc finger protein family. The encoded protein is responsible for membrane trafficking in the endosome and it acts as a scaffolding protein in the TGF-β signaling pathway [12]. 

These genes, were identified to common between the rewired, DEGs of pre-symptomatic and DEGs of symptomatic. The pathways of ubiquitin protein ligase activity, ATP binding, adenyl ribonucleotide, protein serine/threonine kinase activity, purine ribonucleotide triphosphate binding, ubiquitin-protein transferase activity, protein kinase activity and RNA binding were identified from the DEGS that are common between the rewired, DEGs of pre-symptomatic and DEGs of symptomatic HD. 

The ubiquitin-proteasome system (UPS) is a highly dynamic and vital intracellular molecular machinery for the biological process of protein degradation and maintenance of protein homeostasis [24]. It plays a role in cell cycle, cell differentiation, stress signaling, inflammatory response and it has a role in cell fate and cell specification [24]. UPS facilitates the degradation of misfolded proteins and the removal of damaged soluble proteins. The mechanism of action of the UPS is a two-step process involving (1) ubiquitination and (2) proteolytic degradation of polyubiquitinated [24]. In HD, it has been suggested that mHTT can impair the UPS either by saturating the system, or by providing excess protein or by directly inhibiting the UPS [25]. One study identified a decrease in proteasome activity in HD brains, however the exact mechanism of UPS dysfunction remains unknown. The mHTT is not degraded but accumulates and forms insoluble intracellular aggregates. The mHTT aggregates are unable to be removed by the UPS and therefore, alter the normal functioning and efficiency of the UPS [24]. 

An additional pathway identifed is the protein serine/threonine kinase activity, this is a kinase enzyme that phosphorylates the OH group of serine or threonine [26]. There are various such as protein kinase A and C, which are second messengers, MAPKs which regulate cellular functions such as gene expression, mitosis, differentiation, cell survival and apoptosis, Raf kinases, that stimulate growth of cells and several additional serine/threonine family of kinases. A previous study, identified that MAPK11 and huntingtin interacting protein 1 (HIP3K) as positive modulators of mHTT levels in cells and in vivo. These kinase regulate mHTT via their kinase activity therefore, suggesting that inhibition of these kinase may be used as possible therapeutic treatment [27]. Furthermore, the kinases effect on the HTT levels are mHTT-dependent. Therefore, providing a feedback loop by which mHTT increases its own levels resulting in mHTT accumulation and disease progression. MAPK11 knockout, was identified to, significantly rescue disease-relevant behavioral phenotypes in a knock-in HD mouse model [27]. The *CACNA1I,* was previously discussed and identified as the most highly rewired node using DyNet. 

*DNAJB14,* acts as a co-chaperone with heat shock proteins (HSPA8/Hsc70); it plays a role in promoting protein folding and trafficking and preventing protein aggregation [12]. ATP citrate lyase (ACLY), is an ATP citrate lyase enzyme and it is responsible for the synthesis of cytosolic acetyl-Co-enzyme A (CoA) in many tissues. The enzyme, catalyzes the formation of acetyl-CoA and oxaloacetate from citrate and CoA. The product, acetyl-CoA, is involved in a number of biological pathways such as and cholesterogenesis. In neuronal cells, *ACLY* may play a role in the biosynthesis of the neurotransmitter acetylcholine [12]. *BAG5* is an anti-apoptotic protein that interacts with numerous apoptosis and growth-related proteins including B-cell lymphoma 2 (BCL-2), Raf kinase, steroid hormone receptors and growth factor receptors [12]. Some of the above-mentioned genes were observed in the re-wired and DEGs of pre-symptomatic HD genes. 

The pathways of phosphatidylinositol bisphosphate kinase activity, cyclic adenosine monophosphate (cAMP) response element binding protein, tyrosine kinase and voltage-gated ion channels have a vital functional role in the regulation of the postsynaptic membrane potential. Phosphatidylinositol bisphosphate kinase activity is a lipid second messenger with a number of biological functions such as signal transduction, vesicle trafficking, adhesion, and [28]. There are limited studies, on the effect of phosphatidylinositol bisphosphate kinase activity on HD. A previous study, investigated the in vitro knock down or pharmacological inhibition of the PIP4K isoform, PIP4K2C the study observed a reduction in mHTT aggregates by increasing autophagy. Therefore, proposing that the kinase could be a potential therapeutic target for HD treatment [28]. The cAMP response element binding protein was also identified, the cAMP-response element binding protein (CREB) is an intracellular protein responsible for the regulation of genes that are vital in DA neurons. DA was observed to effect CREB phosphorylation via the G protein-coupled receptors [29]. Dysfunctional CREB has been hypothesized to lead to neuronal cell death in HD. A previous study, observed the upregulation of CRE-dependent transcription in the striatum, hippocampus and cortex of the R6/2 HD mouse model. Additionally, an increase in cAMP response element binding protein phosphorylation and an increase in the levels of CREB-regulated gene product, such as the CCAAT//enhancer binding protein β, was also observed in the HD mouse model [30].

The dopaminergic synapse pathway was identified in symptomatic HD patients. This pathway was common between PathwayConnector and PathWalks. Some of the identified metabolites include dopamine, homovanillate, L-tyrosine, Ca^2+^ and diacylglycerol. Dopamine (DA) plays a role in controlling movement, behavior and addiction [31]. Alternations in DA balance in the striatum, result in neurodegenerative diseases such as PD and HD. Changes in DA brain content and receptor number contributes to movement behavioral and cognitive impairments observed in HD patients [31]. During the early hyperkinetic HD stage, the DA levels are increased and expression of DA receptors is decreased in contrast to the late akinetic HD stage, were DA levels are significantly reduced [31]. One study, using a positron emission tomography (PET), observed a decrease in striatal D1 and D2 DA receptor density in advanced HD patients but also in asymptomatic HD patients. This further indicates that the DA signaling is dysregulated in the early HD stages [31]. Striatal and cortical loss of DA receptors in pre-symptomatic and early symptomatic HD stage, was correlated with early cognitive decline, which may possibly reflect altered synaptic plasticity leading to the impairment of attention, executive function, learning and memory [31]. 

Homovanillate also known as homovanillic acid (HVA), is a catecholamine metabolite produced by reaction of monoamine oxidase and catechol-O-methyltransferase onto DA [32]. HVA is used for the exposure of oxidative enzymes and it is also associated with DA concentration in the brain. Furthermore, HVA is also used as a biological marker of metabolic stress triggered by 2-deoxy-D-glucose [33]. 

One study investigated the HVA levels obtained from the cerebrospinal fluid (CSF) of HD patients and compared them to controls. The HVA concentration was identified to be decreased in the CSF of HD patients compared to controls [34]. This decrease may possibly indicate either a decreased number or a decreased activity of dopaminergic neurons. An additional study, investigated the levels of plasma HVA in 116 HD patients, which consisted of 29 pre-symptomatic and 90 symptomatic HD patients and controls [35]. There was no significant difference in the plasma HVA levels of the pre-symptomatic HD patients compared to controls. However, the HVA levels were significantly higher in symptomatic HD patients. This increase was positively associated with disease severity and functional capacity of patients [35]. 

However, there is conflicting evidence regarding the measurement of DA and its metabolite HVA in post-mortem HD brain tissue. Some studies have found either normal HVA levels [35,36], or normal DA levels and reduced HVA [35,37] or reduced DA and HVA levels [34,35]. However, more research is required to identify the presence or absence of HVA concentration in HD patients and the effects this might have on disease progression and severity. 

The pathways of (i) ubiquinone and other terpenoid quinone biosynthesis, (ii) hippo signaling in multiple species and (iii) phosphatidylinositol signaling, were identified as the top pathways via the odd ratio analysis of pre-symptomatic versus symptomatic HD group, pre-symptomatic and symptomatic HD groups respectively. Hippo signaling is involved in controlling organ size in animals, through the regulation of cell proliferation and apoptosis, where the key signaling component is the protein kinase Hippo (Hpo) [38]. The inhibition of Hippo results in tumorigenesis and its activation may play a role in neurodegeneration [39]. 

A study investigated the role of the Hippo signaling dysregulation in human HD brains and neuronal system cells [39]. A decrease in the nuclear Yes associated protein (YAP) was observed. The study found, that YAP nuclear activity becomes altered in HD and this may be linked to HD pathogenesis. Additionally, the YAP levels in the cortex of HD brain tissues and HD embryonic stem-derived neuronal stem cells were observed to be decreased. A reduction in YAP mediates oxidative stressed, which leads to neuronal cell death and a decrease in neuronal cell survival [39]. 

YAP was also identified to interact with HTT and chaperone proteins. This interaction was not altered in the presence of mHTT. YAP also interacts with the transcriptional enhancer activator domain (TEAD). The YAP/TEAD complex promotes transcription of pro-survival genes, which are involved in cell survival and proliferation and inhibit apoptosis [39]. The YAP/TEAD interaction and expression of Hippo signaling gene were identified to be altered in HD cells [39]. 

Phosphatidylinositol plays an important role in intracellular signaling in response to extracellular signals [40]. Phosphatidylinositol undergoes a rapid turnover and is responsible for the activation of second messengers such as diacylglycerol, inositol 1,4,5-trisphosphate, phosphatidylinositol 3,4-bisphosphate and phosphatidylinositol 3,4,5-trisphosphate. The phosphatidylinositol signaling pathway plays a role in cell proliferation, survival and metabolism and in additional functions including cell migration, endocytosis and membrane dynamics [40]. 

The phosphoinositide enzyme PI5P has been recently discovered and is located in the plasma membrane, nucleus and Golgi. The possible function of PI5P in the cell, is that it may be involved in regulating chromatin function and transcriptional regulation in the nucleus [28]. 

Previous studies, investigated in vitro knock-down and pharmacological inhibition of the phosphatidylinositol 5-phosphate 4-kinase (PIP4K) isoform, phosphatidylinositol-5-phosphate 4-kinase Type 2 gamma (PIP4K2C). The study identified a decrease in mHTT protein aggregates was observed by increasing basal autophagy. This study suggested that the PIP4K2C kinase may be a potential target for the treatment progressive neurodegenerative diseases such as HD [28]. However, more research is needed to investigate and understand the phosphoinositide enzymes and their role in HD. The HD related pathways which were identified in the pre-symptomatic and symptomatic HD patients using PathwayConnector [15] were then compared to the HD-related pathways obtained through our multi-source data integration approach [for more details on methods and findings see 33]. Some of the common identified pathways between pre-symptomatic HD and symptomatic HD patients and HD related pathways were identified from the multi-source data integration approach [41]. Some of the common pathways include, cocaine addiction, dopaminergic synapse, pathways in cancer, and several other pathways. The common pathways between the multi-source data integration approach and pre-symptomatic and symptomatic HD patients are shown in Table S11.

In [13], the authors performed experimental analysis to identify genes and possible biomarkers between pre-symptomatic and symptomatic HD patients. In that work, they utilized statistical analysis to identify the DEGs in pre-symptomatic and symptomatic HD. However, no pathway analysis tools were used to identify the pathways of either the DEGs or the pathways of the genes used as biomarkers for symptomatic HD. The analysis and biomarker identification focused on symptomatic HD and controls rather than also focusing on possible biomarkers for pre-symptomatic HD. Over the years a number of bioinformatics tools have been developed for the analysis of large scale biological data. Our study, utilizes different bioinformatics tools and analysis for the identification of DEGs, pathways, network re-wiring and metabolite differences between pre-symptomatic and symptomatic HD. Furthermore, tools such as PathwayConnector, adds missing complementary pathways into a network to achieve connectivity between pathway nodes. This leads to a more informative fully connected network of the pre-symptomatic and symptomatic HD networks. DyNet was used to identify and visualize how gene molecular interactions become altered and result in changes in their connectivity and composition in response to cellular signals within a biological network. Our approach, can provide insight into the genes, pathways and metabolites which become altered prior and during HD disease onset and also a better understanding on how these pathways and genes are affected which can lead to pharmacological intervention. 

The strengths of our study include (i) identification of DEGs, pathways and exclusive metabolites between the pre-symptomatic HD and symptomatic HD patients. However, our study is not without its limitations, namely (i) the extremely limited number of HD datasets with blood samples, (ii) blood samples may not sufficiently reveal neurodegeneration in the CNS and the blood samples may not correlate with brain changes in HD, (iii) the small sample size of the dataset used, and (iv) lack of validation of our results in a larger cohort of HD patients. Although the sample size is small, which can have an implication in the significance and reproducibility of the results obtained, it is challenging to find an HD dataset with a large sample size and that consists of two stages due to the rarity of HD. While the sample size is a limitation, it can drive to findings even with an increased level of noise and provide an initial understanding of pathways and metabolites involved in each HD stage before replicating the findings in a larger sample. Although the sample size is small and this can have an implication on the significance and reproducibility of the results obtained, it is challenging to find an HD dataset with a large sample size and consisting of two stages due to the rarity of HD. While the small sample size is a limitation, it can drive to findings even with an increased level of noise and provide an initial understanding of the pathways and metabolites involved in each HD stage before replicating the findings in a larger sample. Although the blood sample does not necessarily correlate with brain changes in HD, it is an easier biological fluid to obtain from HD patients and less invasive compared to lumber puncture to obtain cerebrospinal fluid. We expect that this work will be the basis of future experimental validation of our results in a larger cohort study to validate both the DEGs, pathways, metabolites and the gene expression differences of *CACNA1I* in a larger case-control study of HD patients.

Our in silico analysis identified the biochemical pathways, genes and metabolites between pre-symptomatic and symptomatic HD patients. This analysis can shed light on the genes and pathways which become dysregulated before disease onset as well as during disease onset. Targeted treatments regarding these genes and pathways should be further pursued. Such treatments may potentially result in a delay in disease onset, improvement of clinical symptoms, improvement of energy metabolism and also increase the concentration of down-regulated enzymes of HD. Our findings are in agreement with the bibliography. Based on this work, we identified certain pathways such as TGF-β, dopaminergic synapse and Ca^2+^ signaling to be dysregulated between pre-symptomatic and symptomatic HD patients. Furthermore, we identified metabolites and their exclusive pathways for each HD stage. The dysregulation of these metabolites can contribute to HD pathology. The *CACNA1I* gene was identified to be the most highly rewired gene. Pathways and metabolites identified to be altered or dysfunctional in the pre-symptomatic and symptomatic HD stages can be further experimented on to allow the development of therapeutics which target these genes, pathways and metabolites. This can lead in a delay in the age of onset as well as in a decrease of disease severity and symptom onset in HD patients. 

## 4. Materials and Methods 

**Figure 7:**
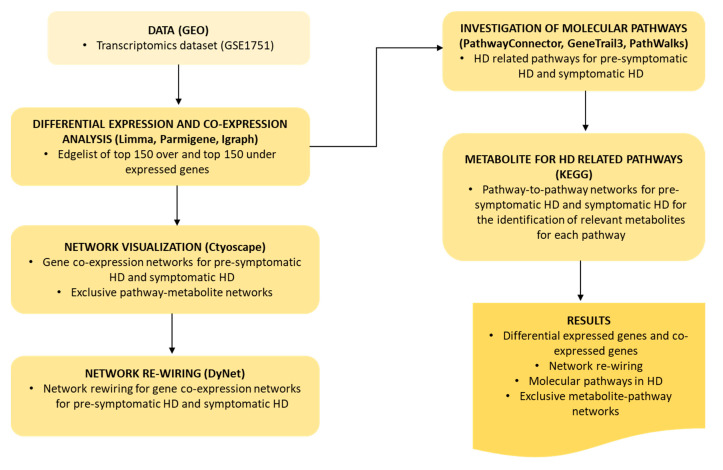
Flowchart of methodology and results.

An HD dataset was obtained from GEO, which was used to identify the DEGs and construct gene co-expression networks for each HD stage. Network visualization of gene co-expression and exclusive pathway-metabolite networks was performed using Cytoscape. Network re-wiring was performed, to identify the mostly highly re-wired node among the two HD stages. Molecular pathways, were investigated using PathwayConnector, GeneTrial3 and PathWalks. KEGG was used to identify the exclusive metabolites for each pathway identified using PathwayConnector. The workflow we applied is illustrated in Figure 7.

### 4.1. Data

A transcriptomics dataset “Human blood expression for Huntington’s disease versus controls” (GSE1751), was obtained from Gene Expression Omnibus [42]. The aforementioned dataset consists of 31 samples, 5 pre-symptomatic, 12 symptomatic HD patients (7 males and 5 females) and 14 healthy controls. No further information was provided on the number of males and females for 5 pre-symptomatic HD patients and healthy controls. 

No additional information such as demographics or clinical information about the samples and the patients were provided in [13]. Neurological status of HD patients was determined using the Unified Huntington’s Disease Rating Scale (UHDRS), which was performed by an experienced neurologist. Symptomatic patients were in stages I or II of HD, based on the total functional capacity scores (TFC) of 7–12 [13]. 

GEO [42] and Repositive [https://repositive.io] were extensively searched for additional datasets, with the criteria (i) to consist of two HD stages and (ii) the biological fluid being blood. However, this was the only dataset found, fitting these criteria. Details on the collection of peripheral blood samples from both cases and controls are in detail in [13]. 

### 4.2. Differential Expression and Gene Co-Expression Analysis 

The linear models for microarray data (Limma) is an R package that allows for the analysis of gene expression data obtained from experiments such as microarrays and RNA-seq. The Limma R package is used to identify the differential expressed genes (DEGs) between cases vs controls or treated vs non-treated individuals [43]. Limma using the adjusted *p*-value was also performed, as illustrated in Tables S19 and S20 for pre-symptomatic and symptomatic respectively. 

Parmigene is an R package that performs parallel estimation of mutual information it is based on estimates from the k-nearest neighbor’s distances and uses algorithms for the reconstruction of gene regulatory networks [44]. The input files used for the parmigene R script include the intensities matrix and top ids for each of the two disease stages. The output obtained is a clr file for controls versus pre-symptomatic and controls versus symptomatic patients. 

Graph network analysis was performed using Igraph [45]. An edgelist for controls versus pre-symptomatic and controls versus symptomatic HD patients was obtained. 

There was a total of 1615 DEGs for pre-symptomatic HD and 3683 DEGs for symptomatic HD. A total of 300 DEGs (top 150 over and top150 under-expressed genes with *p*-value < 0.05), were selected to be analysed further for each HD stage, to avoid noise within our networks and to extract meaningful information from the pre-symptomatic and symptomatic HD network. A further cut-off threshold was applied to the final edgelists for both controls versus HD stages. The weights from the final edgelist were converted to a log function (log (weight)). Therefore, genes and their weights of above 1 and more were used as input into Cytoscape for the construction of gene co-expression networks for controls versus pre-symptomatic and controls versus symptomatic HD patients. 

### 4.3. Network Visualization and Analysis

Gene co-expression networks were constructed and visualized using R’s package igraph [45], for each HD disease stage. The network shown in Figure 2 was visualized in Cytoscape [46] using the edge lists obtained from igraph. A gene co-expression network is an undirected network, consisting of nodes and edges. Each node represents a gene, a pair or group of nodes is connected to an edge if there is a meaningful co-expression relationship between the nodes. Co-expressed networks are of biological significance since the co-expressed genes maybe be controlled by either the same transcriptional regulatory factors, or they be functionally related or they may be part of the same biological pathways and mechanisms or protein complexes [47]. 

Network topology is defined as the way in which nodes and edges are arranged within the network. Network topology analysis, can help in the identification of relevant sub-structures within the network. There are different network metrics such as betweenness, centrality and closeness that can be applied to understand node relationships in a network. Network topology metrics that were calculated for the pre-symptomatic and symptomatic HD gene co-expression networks are described below. 

The degree indicates the number of a node’s neighbors. The pre-symptomatic HD network has a degree value close to 7.5 and the relative density is 1 and the symptomatic is 5 and the relative density is 1. Betweenness is defined as the measure of centrality within a network based on the shortest paths. The relative density in both networks is close to 1. The coreness, measures the importance of a node to disseminate information through the network. Furthermore, the node with more connections to its neighbors are located in the core of the network are more powerful. The pre-symptomatic HD network has a coreness value of 3 × 10^−4^ compared to 1 × 10^−4^ the symptomatic HD network, indicating that the pre-symptomatic HD network is more interlinked. Closeness, measures how short the shortest paths are from a node to all nodes in the network, the relative density is close to 1 for both the pre-symptomatic and symptomatic HD networks. 

### 4.4. Network Re-Wiring

The network rewiring approach allows for the identification and visualization of how molecular interaction networks change in their connectivity and composition in response to cellular signals DyNet is a Cytoscape plug-in that performs network re-wiring [11]. 

DynNet allows for the visualization and analysis of large scale multi-state dynamics molecular interaction networks. There are two modes of analysis, (i) the pairwise mode, compares two networks only and the (ii) multiple comparison mode: compares two or more networks. Each analysis mode assists in the visualization of node and edge variations based on either their presence, absence or the value of selected numeric attribute, for example node abundance and edge weight across the biological networks. When comparing numeric attributes between the two biological networks in the pairwise mode, DyNet calculates the *log2* fold change of the numeric attribute [11]. When comparing two or more networks, the following approach is applied to obtain the node and edge rewiring: the normal variance for the numeric attribute is calculated across all the networks, this then gives a score for the most rewired nodes in the central reference network, which consists of all the input networks you used a input [11]. The most re-wired nodes can be visualized as a dark red whereas nodes that are less re-wired are shown as light pink and nodes with no re-wiring are shown as white in the central reference network. The D_n_ score is known as the rewiring metric and it identifies the most rewired nodes in a network [11].

The gene co-expression networks for the pre-symptomatic and symptomatic HD stages were used as input in the DyNet Cytoscape [11,46] plug-in, to identify the most re-wired nodes, between the pre-symptomatic and symptomatic HD networks. The multiple comparison mode was used for the analysis between the two HD stage networks. 

### 4.5. Investigation of Molecular Pathways Related to HD

#### 4.5.1. PathwayConnector for Complementary Pathway-To-Pathway Networks

PathwayConnector is a web-based tool developed by our group, which allows for the generation and construction of complementary pathway-to-pathway networks, based on the reference pathway network of either KEGG [19] or REACTOME [48] databases for pathway mapping and clustering of pathways. PathwayConnector [15] was used for the identification of HD-related pathways present in the pre-symptomatic and symptomatic HD stages. The top up and down regulated genes identified through Limma, for the pre-symptomatic and symptomatic stages were used as input into PathwayConnector. The output obtained is a cluster of complementary pathways, related to HD.

#### 4.5.2. GeneTrial3 for the Identification of Biological Processes and Pathways

GeneTrail3 is an open source, web application that provides functional analysis for the identification, analysis and visualization of dysregulated biological processes; [17]. The GeneTrail3 tool provides a comprehensive collection of biological processes and signaling pathways for 12 organisms that can be analyzed, either through (i) over-representation analysis (ORA) which compares a reference set of genes to a test set and (ii) gene set enrichment analysis (GSEA), which scores a sorted list of genes [17]. GeneTrial3 was used for the analysis of the top 150 over and 150 under expressed gene lists obtained through Limma for pre-symptomatic and symptomatic HD. The following biological databases and categories were selected (i) Gene Ontology (GO) biological processes, cellular components, molecular function, (ii) KEGG pathways, (iii) Reactome pathways, (iv) Wikipathways, (v) BioCartia pathways, (vi) HumanCyc and (vii) Consensus PathDB-HumanCyc and the reference set selected was all supported genes. The results obtained, include pathway names for each of the databases selected, the number of hits from our gene list, an expected score and an adjusted *p*-value. 

#### 4.5.3. PathWalks Highlighting Pathway Communities

PathWalks is an approach, where a random walker, walks across a pathway-to-pathway network with the help a gene network which was constructed by integrating multi-source information regarding a disease of interest [18]. The two main networks required by PathWalks is (i) a multi-source integration, which is the synthetic gene network that represents integrated information such as gene co-expression, miRNA and physical interactions obtained from biological database and (ii) pathway-to-pathway network. The walker performs random walks on the gene-to-gene network and the nodes visited by the walker indicates the walker’s destination on the pathway-to-pathway network [18].

The top 150 over and 150 under expressed genes for pre-symptomatic and symptomatic HD stages, obtained through Limma were used as input into the PathWalks. The results generated for each HD stage include (i) pathways ranked w.r.t. the count of their respective visits, (ii) edgelists ranked w.r.t. to the count of walker transitions between the respective connected pathways.

The visit counts per pathway were adjusted for the effect of network topology where larger and highly connected pathways are favored against smaller ones. Specifically, using an odds ratio (OR) analysis we defined the ratios between the resulting visit counts when the DEGs of interest where used as an input to PathWalks (guided), to the corresponding counts of a run without any gene input (non-guided). The OR is given by:

$$OR=\frac{P_{i}^{G}/\left(1-P_{i}^{G}\right)}{P_{i}^{U}/\left(1-P_{i}^{U}\right)}$$

$$P_{i}^{G/U}=\frac{F_{i}^{G/U}}{F_{t}^{G/U}}\;i\in \left\{1,2\ldots n\right\}$$

where indices *G* and *U* correspond to the guided and non-guided runs, respectively. $P_{i}^{G/U}$ is the visiting probability of the *ith* pathway calculated as the ration of frequency $F_{i}^{G/U}$ over the total visits $F_{t}^{G/U}$ to all *n* pathways used in the PathWalks reference network. A pathway is considered more likely to be involved in HD for OR values greater than 1. OR values of less than 1 correspond to less relevant pathways to HD. The obtained pathways and corresponding OR values were visualized as a network using R’s igraph package [45]. 

### 4.6. Metabolites for HD Related Pathways

Metabolites are referred to as compounds in the KEGG database. The pathway-to-pathway networks, constructed using PathwayConnector for pre-symptomatic and symptomatic HD stages were then used to identify the relevant metabolites for each pathway, using the KEGG database [19]. 

## 5. Conclusions

In this work, a publicly available dataset consisting of both pre-symptomatic and symptomatic HD patients was analyzed. The DEGs for each HD stage were identified. These genes may become mutated during the early stages of the disease prior to symptom onset. Some of the genes are involved in signaling pathways such as the elimination of reactive oxygen species (ROS) from the cell and the protein folding and proteasome degradation. However, there is a limited number of studies investigating the effect DEGs in HD and overall in neurodegenerative diseases. Our results provide insight into: (i) the genes which become dysregulated in the pre-symptomatic and symptomatic HD patients, (ii) the HD related pathways and (iii) the exclusive metabolites for each HD stage which become dysregulated as the disease progresses from the pre-symptomatic to symptomatic HD stage. However, more research needs to be conducted to understand how abnormal gene expression and alterations in the substrates, proteins and enzymes of these pathways affect both pre-symptomatic and symptomatic HD. A better understanding of the systematic biological changes, which are associated with the genes, proteins and metabolites in HD is vital to provide a further understanding and potentially study the factors involved in the disease development and progression. Furthermore, the development of new therapeutics can target either the genes, proteins or metabolites involved in pathways identified in both the pre-symptomatic and symptomatic HD patients. This may delay the age of onset, improve clinical symptoms and overall improve the quality of life for HD patients. 

## Figures and Tables

**Table 1:** The top-15 ranked pathways obtained from GeneTrial3 for pre-symptomatic HD using WikiPathways.

Rank	Pathway Name	*p*-Value
1	Transforming growth factor-beta (TGF)-beta signaling	0.00235
2	Codeine and morphine metabolism	0.00878
3	Focal adhesion	0.00878
4	PI3K-Akt signaling	0.00878
5	Small cell lung cancer	0.01354
6	Methylene tetrahydrofolate reductase (MTHFR) deficiency	0.02864
7	Chromosomal and microsatellite instability in colorectal cancer	0.03138
8	Development and heterogeneity of the innate lymphoid cell (ILC) family	0.03138
9	Oligodendrocyte specification and differentiation(including remyelination), leading to myelin components for central nervous system (CNS)	0.03138
10	Pregnane X receptor pathway	0.03138
11	Ciliary landscape	0.03437
12	Ectoderm differentiation	0.03437
13	Sleep regulation	0.03667
14	22q11.2 deletion syndrome	0.03806
15	Mesodermal commitment pathway	0.03806

**Table 2:** The top-15 ranked pathways obtained from GeneTrial3 for symptomatic HD using WikiPathways.

Rank	Pathway Name	*p*-Value
1	Small cell lung cancer	0.00028
2	Adipogenesis	0.00100
3	Pregnane X receptor pathway	0.00368
4	Spinal cord injury	0.00374
5	Aryl hydrocarbon receptor netpath	0.00993
6	Integrated breast cancer pathway	0.00993
7	Phosphodiesterases in neuronal function	0.00993
8	Sudden infant death syndrome (SIDS) susceptibility pathways	0.00993
9	Hippo–Yap signaling	0.01068
10	Nuclear receptors meta-pathway	0.01068
11	Pathways affected in adenoid cystic carcinoma	0.01565
12	Non-small cell lung cancer	0.02004
13	Chromosomal and microsatellite instability in colorectal cancer	0.02053
14	Circadian rhythm-related genes	0.02117
15	Ciliary landscape	0.02573

## Supplementary Materials

The following are available online at https://www.mdpi.com/1422-0067/21/19/7414/s1, Table S1: Up and down-regulated genes in pre-symptomatic HD, Table S2: Up and down-regulated genes in symptomatic HD, Table S3: Description of some over and under-expressed genes in pre-symptomatic and symptomatic HD, Table S4: DyNet re-wiring between pre-symptomatic and symptomatic, Table S5: Gene Ontology (GO) biological pathways for rewired and DEGs pre-symptomatic HD, Table S6: GO biological pathways for rewired and DEGs symptomatic HD, Table S7 Pathways related to pre-symptomatic HD, Table S8: Pathways related to symptomatic HD, Table S9: OR analysis pathways for pre-symptomatic and symptomatic HD, Table S10: OR analysis pathways for pre-symptomatic HD, Table S11: OR analysis pathways for symptomatic HD, Table S12: Exclusive metabolite pathways and the number of metabolites related to pre-symptomatic HD; Table S13: Exclusive pathways and the number of metabolites related to symptomatic HD; Table S14: Metabolites exclusive to pre-symptomatic HD; Table S15: Metabolites exclusive to symptomatic HD; Table S16: Exclusive pathways and their respective metabolites related to pre-symptomatic HD, Table S17: Exclusive pathways and their respective metabolites related to symptomatic HD, Table S18: Common pathways between the multi-source integration approach and the pre-symptomatic and symptomatic HD. Table S19: Limma with adjusted *p*-value for pre-symptomatic HD; Table S20: Limma with adjusted *p*-value for symptomatic HD

## Abbreviations

ACLY	ATP citrate lyase
AD	Alzheimer’s Disease
ADORA2A	Adenosine A2a receptor
BCL-2	B-cell lymphoma
BAG5	BAG Co-chaperone 5
Ca^2+^	Calcium cation
CACNA1E	Calcium voltage-gated channel subunit alpha 1S subunit
CAG	Cytosine-adenine-guanine
CASP	Caspases
CBP	Calcium-binding protein
CSF	Cerebrospinal Fluid
COX7B	Cytochrome c oxidase subunit 7B
CNS	Central Nervous System
CREB	cAMP response element binding protein
DA	Dopamine
DEGs	Differentially expressed genes
*DNAJ*	DnaJ heat shock protein family
FDR	False Discovery Rate
FOXO3	Forkhead Box O3
GEO	Gene Expression Omnibus
GO	Gene Ontology
HD	Huntington’s disease
HIP3K	Huntingtin Interacting Protein
HLADRB4	Major Histocompatibility Complex, Class II, DR Beta 4
HTT	Huntingtin
HVA	Homovanillate
InsP3RI	Inositol Trisphosphate Receptor
IL	Interleukin
iPSCs	Induced Pluripotent Stem Cells
ITGA1	Integrin subunit Alpha 1
KCND2	Potassium Voltage-Gated Channel Subfamily D Member 2
KEGG	Kyoto Encyclopedia of genes and genomes
MAP2	Microtubule associated protein 2
MAPK	Mitogen-Activated Protein Kinase
mtDNA	Mitochondrial DNA
MTFRI	Mitochondrial Fission Regulator 1
mHTT	mutant huntingtin
NPCs	Neural Progenitors Cells
OR	Odds Ratio
OXR1	Oxidation resistance 1
PD	Parkinson’s Disease
PET	Positron Emission Tomography
PIP4K	Phosphatidylinositol-5-Phosphate 4-Kinase
PIP4K2C	Phosphatidylinositol-5-Phosphate 4-Kinase Type 2 Gamma
PolyQ	Polyglutamine
RYRI	Ryanodine receptor
ROS	Reactive Oxygen Species
SB	Systems bioinformatics
TFC	Total Functional Capacity
TGF-β	Transforming Growth Factor Beta
TRAF2	TNF receptor-associated factor 2
UHDRS	Unified Huntington’s Disease Rating Score
UPS	Ubiquitin-Proteasome system
VGCC	Voltage-gated calcium channels
