# Supplementary material for: Investigating the Transition of Pre-Symptomatic to Symptomatic Huntington’s Disease Status Based on Omics Data
Source: Int J Mol Sci. 2020 Oct 8;21(19):7414. doi: 10.3390/ijms21197414 (PMC7582902; doi:10.3390/ijms21197414)
Supplement: Supplementary file 1 [file ijms-21-07414-s001.zip › Journal_of_molecular_science_Supplementary_Data 2.docx]

**Table S1.** Over-expressed genes in pre-symptomatic HD

| **Gene names** | | | | | |
| --- | --- | --- | --- | --- | --- |
| *OXR1* | *ITGA1* | *BAG5* | *DNAJB14* | *GREB1* | *PIKFYVE* |
| *SUSD5* | *LAMB1* | *MAP4K3* | *C8orf44-SGK3* | *SLC30A10* | *CEP57* |
| *NABP1* | *AHR* | *PLEKHS1* | *DMXL1* | *ZNF83* | *C5orf28* |
| *NDUFAF7* | *CCND1* | *PSMC6* | *TRMT11* | *DLG1* | *GLCE* |
| ZNF675 | LIN7C | GNAL | BAZ2B | PHTF2 | STAG2 |
| *GRTP1* | *SLC30A1* | *YEATS4* | *DNAJC15* | *SH3GL2* | *SNAPC1* |
| *WDYHV1* | *NUPL1* | *RNF41* | *IRAK3* | *ZNF267* | *ZNF518A* |
| *BBS10* | *IFT57* | *GOLT1B* | *TARDBPP1* | *PMAIP1* | *MOB1A* |
| *FASTKD3* | *USP14* | *MIR22* | *SMARCA5* | *SNX6* | *ZNF107* |
| *MYBL1* | *PLEKHA1* | *SI* | *LAMA4* | *NRIP1* | *ATF2* |
| *BNC1* | *BBS7* | *ADCY8* | *USP1* | *Y16709* | *GRIN2A* |
| *ATRX* | *RAP2C* | *ASF1A* | *DEK* | *TMED7* | *AIMP1* |
| *RNF128* | *IMPACT* | *SLC12A1* | *SLC25A21* | *IREB2* | *GOLGA8N* |
| *TMEM5* | *HSDL2* | *IFIH1* | *CPM* | *CRBN* | *HAUS6* |
| *NIF3L1* | *TRMT13* | *ZDHHC17* | *GATA3* | *AGBL3* | *NFATC4* |
| *EBAG9* | *CDC37L1* | *GOLGA6L4* | *IGLVIVOR22-1* | *CAPZA1* | *CCDC81* |
| *RBM15* | *LTN1* | *CLGN* | *BMI1* | *GATAD1* | *HBZ* |
| *CRIM1* | *FERMT2* | *NDUFA4* | *FASTKD2* | *CCPG1* | *GRAMD1C* |
| *ZNHIT6* | *GUSBP3* | *THAP9* | *UFL1* | *INHBA* | *EXTL1* |
| *HINT1* | *IL18* | *PTPRN2* | *NUS1P3* | *CAPN7* | *TNFAIP6* |
| *APPL1* | *SLC22A3* | *RAB3GAP2* | *UGT2B4* | *SNRPD3* | *ARGLU1* |
| *SCML1* | *MST4* | *C1orf27* | *KRTAP9* | *SACS* | *COPS2* |
| *EFCAB11* | *TMOD3* | *CLTC-IT1* | *CLDND1* | *GJC1* | *F2R* |
| *DDX17* | *ZFYVE16* | *KCTD3* | *MFN1* | *PPIG* | *ITGA4* |
| *CEP135* | *PSME4* | *BRAP* | *POLA1* | *PUS1* | *ZNF140* |

**Table S1.** Under-expressed genes in pre-symptomatic HD-Cont

| **Gene names** | | | | | |
| --- | --- | --- | --- | --- | --- |
| *KLHL41* | *MIR1236* | *CACNA1I* | *PPY* | *TUSC3* | *IKBKAP* |
| *SUSD5* | *LAMB1* | *MAP4K3* | *ARSE* | *PRAMEF12* | *RPS4XP3* |
| *TAC1* | *FEZF2* | *GPR4* | *LOC100506699* | *MTFR1* | *IGHG1* |
| *JMJD6* | *NTN1* | *BTBD18* | *C4orf191* | *HOXC8* | *HPS4* |
| *NRG2* | *LUZP1* | *GINS4* | *DFNA5* | *SRPR* | *TPSD1* |
| *C14orf105* | *PP13* | *SH2B1* | *HLA-DRB4* | *AQP5* | *KCNQ1DN* |
| *MTHFR* | *PTPRB* | *DCHS1* | *NBAS* | *PRSS50* | *PIAS2* |
| *PRRG2* | *SOX12* | *DGCR5* | *HARS* | *SENP3* | *DCAKD* |
| *ZDHHC8P1* | *CRNN* | *PCSK1N* | *RND2* | *CNTN6* | *LRRC48* |
| *NARFL* | *DDR2* | *ATP1B4* | *SKIL* | *MSLN* | *UGT1A1* |
| *GRB7* | *CCKBR* | *GNRH2* | *MED22* | *SIGIRR* | *FAM215A* |
| *HNRNPA1P37* | *GCNT2* | *LOC101929910* | *ACADL* | *NR2F1* | *HYAL1* |
| *KCND2* | *ATP8A2* | *NDRG4* | *DHRS1* | *MAFK* | *E2F2* |
| *CD72* | *MAP2* | *OLIG2* | *MAGEB4* | *TMEM100* | *MIP* |
| *SYNPO2L* | *BEX4* | *LOC102724905* | *CTNNA2* | *GTF2F2* | *TGFB1I1* |
| *NAV2* | *LEPREL1* | *CNN1* | *LAMC1* | *FBXO40* | *ABCC2* |
| *RPL29P7* | *OCA2* | *ORM1* | *B3GAT1* | *HDLBP* | *XAB2* |
| *WDR76* | *SHH* | *AMBRA1* | *FAM134B* | *ZNF749* | *MATN1* |
| *ZXDC* | *YAP1* | *PLP1* | *MRPL40* | *PF4V1* | *CCDC70* |
| *TMEM255A* | *PITX2* | *FOCAD* | *IGHA1* | *ERBB4* | *MGC2889* |
| *LEPRE1* | *TNFRSF12A* | *SIRT5* | *ACLY* | *MRPS34* | *FUCA1* |
| *A2M* | *TSPAN1* | *PRMT8* | *UTP14A* | *MAP1S* | *ZC3H7B* |
| *PRCC* | *IL12RB2* | *LOC440434* | *OTC* | *ZNF227* | *CCDC177* |
| *TMEM57* | *GRIK2* | *CAND2* | *ZSCAN5A* | *ZSCAN5A* | *SLC16A1* |
| *SOAT2* | *MAP6D1* | *LRP5* | *PTPRCAP* | *DNAJC12* | *B4GALT7* |

**Table S2.** Over-expressed genes in symptomatic HD

| **Gene names** | | | | | |
| --- | --- | --- | --- | --- | --- |
| *ZNF267* | *ATF1* | *PIKFYVE* | *C12orf29* | *ANKRD12* | *UFL1* |
| *ZNF107* | *CLK1* | *ZDHHC17* | *PHTF2* | *MOB1A* | *TMEM168* |
| *PSMC6* | *CHMP2B* | *SP3* | *FAM188A* | *DMXL1* | *RB1* |
| *ASF1A* | *TMED7* | *HSPE1-MOB4* | *ACSL4* | *AHR* | *C2CD5* |
| *MBNL2* | *EVI2A* | *SH2D1A* | *TRMT13* | *RAP2C* | *ARGLU1* |
| *BAZ2B* | *PPIP5K2* | *COX7B* | *GOLGA8N* | *MYBL1* | *ABHD3* |
| *CCPG1* | *C6orf211* | *LTN1* | *SMARCA5* | *ARID4A* | *MAP4K3* |
| *ZNF83* | *PLSCR1* | *CASP3* | *HINT1* | *BMI1* | *NEK7* |
| *PCNP* | *CEP135* | *TVP23B* | *RBM15* | *DEK* | *NDUFA4* |
| *SLC25A24* | *ZFYVE16* | *IRAK3* | *OXR1* | *COPS2* | *TMEM165* |
| *SACM1L* | *MTHFD2* | *CLEC2B* | *RNF6* | *CMPK1* | *NOL8* |
| *KIAA1109* | *SNX10* | *EID1* | *COMMD8* | *ZNF518A* | *ANKRD49* |
| *MICU2* | *SLC35A1* | *CCP110* | *CLDND1* | *HAT1* | *SRSF10* |
| *ACTG1P4* | *AIMP1* | *TWF1* | *HLA-DQA1* | *MBNL1* | *SLC35A5* |
| *CXCL8* | *SRSF11* | *RPL9* | *RNF138* | *NRIP1* | *MIR1304* |
| *NAMPT* | *CDC14A* | *ERGIC2* | *CDC73* | *STK3* | *ERBB2IP* |
| *IFRD1* | *BUB3* | *C1D* | *CD2AP* | *FNDC3A* | *DDX50* |
| *SMCHD1* | *BBS10* | *ACTR6* | *MST4* | *SLMO2* | *YIPF4* |
| *GNG10* | *PPP1CB* | *ACN9* | *NUPL1* | *UBA3* | *DENND4A* |
| *PDZD8* | *ITGA4* | *ACAP2* | *KRCC1* | *PELI1* | *RPS3A* |
| *LOC100996668* | *RALGAPA1* | *PCMTD2* | *CKS2* | *CBR4* | *KIF2A* |
| *LOC145783* | *VCAN* | *MEX3C* | *LBR* | *CHMP5* | *EMC2* |
| *PDE6D* | *CASP8AP2* | *APPL1* | *IGJ* | *APIP* | *PTGS2* |
| *P2RY14* | *MME* | *SECISBP2L* | *ITSN2* | *CSGALNACT2* | *PTX3* |
| *TMEM123* | *PSMA3* | *C2CD2L* | *AGL* | *CTBP2* | *SLC38A2* |

**Table S2.** Under-expressed genes in symptomatic HD-Cont

| **Gene names** | | | | | |
| --- | --- | --- | --- | --- | --- |
| *HLA-DQA1* | *C2CD2L* | *UBE2O* | *DKK2* | *HLA-DQB1* | *RP4-621B10.8* |
| *GP1BB* | *CNTN6* | *TECR* | *FMO3* | *SEMA3E* | *NENF* |
| *AF198444* | *EPB42* | *CTSZ* | *ADRA2A* | *RFPL1* | *TCF3* |
| *ZNF652* | *DNAJB2* | *ANKRD7* | *SRPX2* | *SLC25A31* | *RGS13* |
| *ZIC4* | *SLC4A8* | *APBA1* | *CLIC3* | *LPA* | *TTC27* |
| *MAFK* | *VPS39* | *LRRC17* | *PTOV1* | *OR7E156P* | *HAPLN2* |
| *PDGFA* | *MAGEB1* | *RPGRIP1L* | *ADORA2A* | *CLU* | *KCNJ5* |
| *PDZD2* | *GPD1* | *PBXIP1* | *RXRA* | *BRPF1* | *CDKN1C* |
| *AKR1B10* | *FKBP8* | *TCF7L2* | *LOC100506282* | *TMPRSS5* | *GOSR1* |
| *CLCA3P* | *ASCC2* | *CTRB1* | *PP14571* | *PNPLA2* | *ANKZF1* |
| *CPS1-IT1* | *HSPB8* | *ACTR3P2* | *BTBD18* | *TPM2* | *ALDOB* |
| *LARGE* | *PVALB* | *F5* | *RP11-15P13.1* | *SHARPIN* | *MAT1A* |
| *PARD3* | *MGMT* | *FOXO3* | *TMSB4Y* | *POU3F2* | *CACNA1E* |
| *MYOD1* | *NRGN* | *TSSC4* | *SCUBE3* | *WISP1* | *NCOA2* |
| *HYAL1* | *GPR22* | *SF3A2* | *NADSYN1* | *RPLP2* | *POLA2* |
| *CHERP* | *ADIRF* | *F2RL3* | *CYP2B7P* | *TSGA10* | *HOXA11* |
| *CTDSPL* | *FARSA* | *MEA1* | *LY6E* | *COL4A6* | *LOC100130331* |
| *CCNT1* | *GNGT1* | *BRD4* | *DRP2* | *CCDC121* | *DCAKD* |
| *STAG3L3* | *ARL17A* | *SPACA1* | *GPER1* | *NEUROD6* | *ARPC4* |
| *SLC6A9* | *SIT* | *CLPB* | *SCGB2A1* | *SHCBP1L* | *ACKR1* |
| *LOC101060747* | *AQP3* | *DDX49* | *DHRS2* | *ESYT1* | *TBL1X* |
| *ASAP1-IT1* | *PDE6C* | *LOC101930075* | *ANGPT2* | *MAN1B1* | *CELA3B* |
| *SLC48A1* | *PTPRCAP* | *LOC100287590* | *TBL3* | *KDM2A* | *GUCY1A2* |
| *LOC101927458* | *SEMA6C* | *MAP4K1* | *ANXA2P3* | *RUNX1-IT1* | *PRPF6* |
| *LMTK2* | *RPL27A* | *DNAJC12* | *CLDN17* | *DDR1-AS1* | *SGCB* |

**Table S3.** Description of some over and under-expressed genes in pre-symptomatic and symptomatic HD patients

| **Gene name** | **Description** | **HD patient stage** | **Reference** |
| --- | --- | --- | --- |
| *OXR1* | Protective role against oxidative damage | Pre-symptomatic (over-expressed) | [50, 51] |
|  |  |  |  |
| *DNAJC15* | Negative regulator of oxidative phosphorylation and controls mitochondrial generation of ATP | Pre-symptomatic (over-expressed) | [50] |
|  |  |  |  |
| *IL-18* | Pro-inflammatory cytokine that increases natural killer cell activity and stimulates interferon gamma production in T-helper type 1 cell | Pre-symptomatic (over-expressed) | [50; 52] |
|  |  |  |  |
| *MAP4K3* | Activation of effector proteins in the c-Jun signaling pathways and it may play a role in response to environmental stress | Pre-symptomatic (over-expressed) | [23, 50] |
|  |  |  |  |
|  |  |  |  |
| *COX7B* | Final stage of oxidative phosphorylation, it catalyses the transfer of electrons from a reduced cytochrome c to molecular oxygen and it also plays a role in the development of the CNS | Symptomatic (over-expressed) | [23] |
|  |  |  |  |
| *CASP3* | Activation and cleavage of caspases 6, 7, and 9, which lead to the activation of the apoptosis cascade. Caspases are involved in numerous signaling pathways besides apoptosis such as necrosis and inflammation | Symptomatic (over-expressed) | [23] |
|  |  |  |  |
| *CACNA1E* | receptor (RYRI), this triggers the release of Ca^2+^ from the sarcoplasmic reticulum and eventually resulting in muscle contraction. Mutations lead to both psychiatric and neurological disorders. | Symptomatic (under-expressed) | [23, 53] |
|  |  |  |  |
| *ADORA2A* | Implicated in inflammatory and neurodegenerative diseases. | Symptomatic (under-expressed) | [23] |
|  | Part of the G protein coupled receptors. respond to various extracellular signals and activate intracellular signal transduction pathways |  |  |
|  |  |  |  |
| *SLC4A8* | responsible for the transport of sodium and bicarbonate ions across the cell membrane, they also play a vital role in pH regulation in neuronal cell, alternations and abnormal function of solute carriers can result in neurodegenerative disease | Symptomatic (under-expressed) | [23] |

**Table S4.** DyNet rewiring score and genes of pre-symptomatic and symptomatic HD network

| DyNet central reference network between pre-symptomatic and symptomatic HD | | | | | |
| --- | --- | --- | --- | --- | --- |
| Dn-Score(Degree corrected) | **DyNet Rewiring (Dn-score)** | **Edge count** | **Gene name** | **Presence/absence of genes in Pre-HD** | **Presence/ absence of genes in HD** |

0.5 6 12 CACNA1I TRUE FALSE

0.5 4.5 9 CNTN6 TRUE TRUE

0.5 4 8 DEK TRUE TRUE

0.5 3.5 7 MST4 TRUE TRUE

0.5 3.5 7 ZFYVE16 TRUE TRUE

0.5 3.5 7 LTN1 TRUE TRUE

0.5 3.5 7 AF198444 FALSE TRUE

0.5 3.5 7 SOX12 TRUE FALSE

0.5 3.5 7 SNRPD3 TRUE FALSE

0.5 3.5 7 DNAJB14 TRUE FALSE

0.5 3.5 7 EPS8L3 TRUE FALSE

0.5 3.5 7 HSDL2 TRUE FALSE

0.5 3 6 RBM15 TRUE TRUE

0.5 3 6 MAPK43 TRUE TRUE

0.5 3 6 CEP135 TRUE TRUE

0.5 3 6 NUPL1 TRUE TRUE

0.5 3 6 DCAKD TRUE TRUE

0.5 3 6 MTHFR TRUE FALSE

0.5 3 6 GRAMD1C TRUE FALSE

0.5 3 6 OTC TRUE FALSE

0.5 3 6 PRAMEFI2 TRUE FALSE

0.5 3 6 LIN7C TRUE FALSE

0.5 3 6 ATF2 TRUE FALSE

**Table S5.** GO biological pathways for rewired and DEGs pre-symptomatic HD

| **Pathway** | **p-value** | **Combined score** | **Genes** |
| --- | --- | --- | --- |
| Ribosomal Large Subunit Binding (GO:0043023) | 0.005487568 | 946.412758 | LTN1 |
| MAP Kinase Activity (GO:0008349) | 0.005487568 | 946.412758 | MAP4K3 |
| 1-Phosphatidylinositol Binding (GO:0005545) | 0.015888706 | 258.8841717 | ZFYVE16 |
| Kinase Activity (GO:0016301) | 0.039296097 | 80.91575164 | DCAKD;MAP4K3 |
| Phosphatidylinositol-3,4,5-Trisphosphate Binding (GO:0005547) | 0.039778377 | 79.6156 | ZFYVE16 |
| Protein Transporter Activity (GO:0008565) | 0.00675955 | 78.6897483 | ZFYVE16 |
| Phosphatidylinositol Phosphate Binding (GO:1901981) | 0.008160401 | 68.69231345 | ZFYVE16 |
| Phosphatidylinositol Binding (GO:0035091) | 0.048900391 | 60.35939767 | ZFYVE16 |
| Ubiquitin-Like Protein Ligase Activity (GO:0061659) | 0.089221858 | 25.98526049 | LTN1 |
| Ubiquitin Protein Ligase Activity (GO:0061630) | 0.091976707 | 24.85645754 | LTN1 |
| ATP Binding (GO:0005524) | 0.120453166 | 16.59995505 | MAP4K3 |
| Adenyl Ribonucleotide Binding (GO:0032559) | 0.131088144 | 14.56548621 | MAP4K3 |
| RNA Binding (GO:0003723) | 0.169523439 | 9.645456947 | RBM15;DEK |
| Protein Serine/Threonine Kinase Activity (GO:0004674) | 0.181294986 | 8.624393028 | MAP4K3 |

**Table S6.** GO biological pathways for rewired and DEGs symptomatic HD

| **Pathway** | **p-value** | **Combined score** | **Genes** |
| --- | --- | --- | --- |
| Manganese Ion Transmembrane Transporter Activity (GO:0005384) | 0.006583 | 761.1095257 | SLC30A10 |
| CAMP Response Element Binding (GO:0035497) | 0.007676 | 632.4280876 | ATF2 |
| CAMP Response Element Binding Protein Binding (GO:0008140) | 0.008768 | 538.2601143 | ATF2 |
|  |  |  |  |
| Adenyl-Nucleotide Exchange Factor Activity (GO:0000774) | 0.012037 | 365.2733585 | BAG5 |
| Adenyl Nucleotide Binding (GO:0030554) | 0.012037 | 365.2733585 | BAG5 |
| Rac Guanyl-Nucleotide Exchange Factor Activity (GO:0030676) | 0.015295 | 271.4427057 | EPS8L3 |
| Oxidoreductase Activity, Acting On The CH-NH Group Of Donors, NAD Or NADP As Acceptor (GO:0016646) | 0.019624 | 198.5352854 | MTHFR |
| Phosphatidylinositol-4,5-Bisphosphate 3-Kinase Activity (GO:0046934) | 0.002518 | 160.0074223 | ERBB4;NRG2 |
| Zinc Ion Transmembrane Transporter Activity (GO:0005385) | 0.023935 | 154.2323308 | SLC30A10 |
| Telomerase RNA Binding (GO:0070034) | 0.023935 | 154.2323308 | SNRPD3 |
| Phosphatidylinositol Bisphosphate Kinase Activity (GO:0052813) | 0.002741 | 151.0703443 | ERBB4;NRG2 |
|  |  |  |  |
| Voltage-Gated Ion Channel Activity Involved In Regulation Of Postsynaptic Membrane Potential (GO:1905030) | 0.02501 | 145.7902804 | CACNA1I |
| Voltage-Gated  Sodium Channel Activity (Go:0005248) | 0.02501 | 145.7902804 | CACNA1I |
| Phosphatidylinositol 3-Kinase Activity (GO:0035004) | 0.003134 | 137.932383 | ERBB4;NRG2 |
| Epidermal Growth Factor Receptor Binding (GO:0005154) | 0.027156 | 131.1330613 | ERBB4 |
| Enhancer Binding (GO:0035326) | 0.028227 | 124.7364238 | ATF2 |
| Hsp70 Protein Binding (GO:0030544) | 0.029298 | 118.8634537 | DNAJB14 |
| Transition Metal Ion Transmembrane Transporter Activity (GO:0046915) | 0.034633 | 95.53864758 | SLC30A10 |
| SnRNA Binding (GO:0017069) | 0.03888 | 82.00171642 | SNRPD3 |
| Voltage-Gated Calcium Channel Activity (GO:0005245) | 0.039939 | 79.12509753 | CACNA1I |
| Divalent Inorganic Cation Transmembrane Transporter Activity (GO:0072509) | 0.040997 | 76.41735943 | SLC30A10 |
| Atpase Regulator Activity (GO:0060590) | 0.040997 | 76.41735943 | BAG5 |
| RNA Polymerase II Transcription Coactivator Activity (GO:0001105) | 0.042054 | 73.86462627 | SOX12 |
| Peptide-Lysine-N-Acetyltransferase Activity (GO:0061733) | 0.042054 | 73.86462627 | ATF2 |
|  |  |  |  |
| Sodium Channel Activity (GO:0005272) | 0.04311 | 71.45447536 | CACNA1I |
| Histone Acetyltransferase Activity (GO:0004402) | 0.044165 | 69.1757536 | ATF2 |
| RNA Polymerase II Activating Transcription Factor Binding (GO:0001102) | 0.05047 | 57.7636852 | ATF2 |
| Flavin Adenine Dinucleotide Binding (GO:0050660) | 0.051517 | 56.17141644 | MTHFR |
| Protein Tyrosine Kinase Activity (GO:0004713) | 0.011256 | 55.49610409 | ERBB4;NRG2 |
| Transcriptional Activator Activity, RNA Polymerase II Transcription Factor Binding (GO:0001190) | 0.053607 | 53.20130759 | SOX12 |
| RNA Polymerase II Distal Enhancer Sequence-Specific DNA Binding (GO:0000980) | 0.055693 | 50.48765794 | ATF2 |
| Rho Guanyl-Nucleotide Exchange Factor Activity (GO:0005089) | 0.06296 | 42.60782564 | EPS8L3 |
| Transmembrane Receptor Protein Tyrosine Kinase Activity (GO:0004714) | 0.065027 | 40.72955232 | ERBB4 |
| Transmembrane Receptor Protein  Kinase Activity (GO:0019199) | 0.066059 | 39.84183398 | ERBB4 |
| Transcription Factor Activity, RNA Polymerase II Distal Enhancer Sequence-Specific Binding (GO:0003705) | 0.068119 | 38.16058849 | ATF2 |
| Enhancer Sequence-Specific DNA Binding (GO:0001158) | 0.068119 | 38.16058849 | ATF2 |
| Mitogen-Activated Protein Kinase Kinase Binding (GO:0031434) | 0.069147 | 37.36393926 | ERBB4 |
| Transcription Regulatory Region DNA Binding (GO:0044212) | 0.007673 | 35.51344766 | ATF2;ERBB4;SOX12 |
| Activating Transcription Factor Binding (GO:0033613) | 0.072226 | 35.13318204 | ATF2 |
| MAP Kinase Activity (GO:0004709) | 0.082417 | 29.09042763 | ERBB4 |
| RNA Polymerase II Transcription Cofactor Activity (GO:0001104) | 0.088481 | 26.24421865 | SOX12 |
| Single-Stranded DNA Binding (GO:0003697) | 0.091499 | 24.9888298 | NABP1 |
| Growth Factor  Receptor Binding (GO:0070851) | 0.096507 | 23.10418247 | ERBB4 |
| Ion Channel Activity (GO:0005216) | 0.097505 | 22.75513849 | CACNA1I |
| Calcium Channel Activity (GO:0005262) | 0.097505 | 22.75513849 | CACNA1I |
| Protein Kinase Binding (GO:0019901) | 0.016358 | 22.6614906 | ATF2;BAG5;ERBB4 |
| Cation Transmembrane Transporter Activity (GO:0008324) | 0.101488 | 21.44153418 | CACNA1I |
| Transcriptional Activator Activity, RNA Polymerase II  Transcription Regulatory Region Sequence-Specific Binding (GO:0001228) | 0.038507 | 20.8508535 | ATF2;SOX12 |
|  |  |  |  |
| Voltage-Gated Cation Channel Activity (GO:0022843) | 0.104465 | 20.53550986 | CACNA1I |
| Transcription Coactivator Activity (GO:0003713) | 0.040247 | 20.07324784 | ATF2;SOX12 |
| Transcription Regulatory Region Sequence-Specific DNA Binding (GO:0000976) | 0.040498 | 19.96580247 | ATF2;SOX12 |
| Sequence-Specific Double-Stranded DNA Binding (GO:1990837) | 0.116276 | 17.46578727 | SOX12 |
| RNA Polymerase II Transcription Factor Binding (GO:0001085) | 0.125037 | 15.62090061 | ATF2 |
| Cation Channel Activity (GO:0005261) | 0.148941 | 11.85682586 | CACNA1I |
| Kinase Binding (GO:0019900) | 0.076492 | 11.18124768 | ATF2;BAG5 |
| Transcriptional Activator Activity, RNA Polymerase II Core Promoter Proximal Region Sequence-Specific Binding (GO:0001077) | 0.175888 | 9.028096745 | SOX12 |
| Protein Kinase Activity (GO:0004672) | 0.10835 | 7.87662491 | ERBB4;NRG2 |
| RNA Polymerase II Regulatory Region  DNA Binding (GO:0001012) | 0.199353 | 7.29388202 | ATF2 |
| Regulatory Region DNA Binding (GO:0000975) | 0.219578 | 6.152789734 | ERBB4 |
| Actin Binding (GO:0003779) | 0.245226 | 5.030685324 | EPS8L3 |
| ATP Binding (GO:0005524) | 0.246067 | 4.998752758 | ACLY |
| Adenyl Ribonucleotide Binding  (GO:0032559) | 0.265983 | 4.315156217 | ACLY |
| Transcription Factor Activity, RNA Polymerase II Core Promoter Proximal Region Sequence-Specific Binding (GO:0000982) | 0.266802 | 4.289765046 | SOX12 |
| Ubiquitin Protein Ligase Binding (GO:0031625) | 0.270069 | 4.190391401 | BAG5 |
| Ubiquitin-Like Protein Ligase Binding (GO:0044389) | 0.28059 | 3.889995229 | BAG5 |
| DNA Binding (GO:0003677) | 0.257501 | 2.76235803 | NABP1;SOX12 |
| Purine Ribonucleoside Triphosphate Binding (GO:0035639) | 0.356094 | 2.370431505 | ACLY |
| RNA Polymerase II Regulatory Region Sequence-Specific DNA Binding (GO:0000977) | 0.400813 | 1.80684039 | ATF2 |
| Protein Homodimerization Activity (GO:0042803) | 0.524408 | 0.883741683 | ERBB4 |
| RNA Binding (GO:0003723) | 0.794445 | 0.15082377 | SNRPD3 |

**Table S7.** Pathways related to pre-symptomatic HD

| **Pathway** | **KEGG Reference** | **Combined score** |
| --- | --- | --- |
| **INITIAL PATHWAYS** |  |  |
| Glycosaminoglycan biosynthesis | hsa00534 | 43.580 |
| Small cell lung cancer | hsa05222 | 29.228 |
| ECM receptor interaction | hsa04512 | 19.715 |
| Inflammatory bowel disease | hsa05321 | 16.858 |
| Bile secretion | hsa04976 | 14.336 |
| Starch and sucrose metabolism | hsa00500 | 10.575 |
| Calcium signaling | hsa04020 | 10.254 |
| Focal adhesion  HTLV-1 infection | hsa04510  hsa05166 | 7.872  6.581 |
| Pathways in cancer | hsa05200 | 6.041 |
| **COMPLEMENTARY PATHWAYS** |  |  |
| Ascorbate and aldarate metabolism | hsa00053 | 13.766 |
| African trypanosomiasis | hsa05143 | 8.902 |
| Pentose and glucuronate interconversions | hsa00040 | 8.479 |
| Amoebiasis | hsa05146 | 7.341 |
| Thyroid hormone synthesis | hsa04918 | 6.75 |
| Bladder cancer | hsa05219 | 6.743 |
| Porphyrin & chlorophyll metabolism  Gastric acid secretion | hsa00860  hsa04971 | 6.457  6.226 |
| Arrhythmogenic right ventricular cardiomyopathy | hsa05412 | 6.226 |
| Type I diabetes mellitus | hsa04940 | 6.189 |
| Hepatitis B | hsa05161 | 6.096 |
| Proteasome | hsa03050 | 5.936 |
| Carbohydrate digestion & absorption | hsa04973 | 5.697 |
| Hippo signaling pathway | hsa04390 | 5.478 |
| Drug metabolism | hsa00983 | 5.472 |
| Other glycan degradation | hsa00511 | 5.312 |
| Aldosterone synthesis and secretion | hsa04925 | 5.192 |
| Toxoplasmosis | hsa05145 | 5.161 |
| Intestinal immune network for IgA production | hsa04672 | 5.057 |
| Glycosaminoglycan degradation | hsa00531 | 4.868 |
| Cocaine addiction | hsa05030 | 4.866 |
| TGF-beta signaling pathway | hsa04350 | 4.818 |
| Insulin secretion | hsa04911 | 4.702 |
| PI3K-Akt signaling pathway | hsa04151 | 4.614 |
| Mineral absorption | hsa04978 | 4.513 |
| Chondroitin sulfate | hsa00532 | 4.477 |
| Steroid biosynthesis | hsa00100 | 4.477 |
| One carbon pool by folate | hsa00670 | 4.477 |
| Arginine biosynthesis | hsa00220 | 4.477 |
| cGMP-PKG signaling pathway | hsa04022 | 4.458 |
| Hematopoietic cell lineage | hsa04640 | 4.373 |
| Endometrial cancer | hsa05213 | 4.350 |
| Salivary secretion | hsa04970 | 4.271 |
| Endocytosis | hsa04144 | 4.220 |
| Dilated cardiomyopathy | hsa05414 | 4.171 |
| Longevity regulating pathway | hsa0421 | 3.801 |
| Non-small cell lung cancer | hsa05223 | 3.771 |
| Circadian entrainment | hsa04713 | 3.716 |
| Protein export | hsa03060 | 3.550 |
| Proximal tubule bicarbonate reclamation | hsa04964 | 3.550 |
| Steroid hormone biosynthesis | hsa00140 | 3.521 |
| Viral myocarditis | hsa05416 | 3.405 |
| Colorectal cancer | hsa05210 | 3.084 |
| Glycosphingolipd biosynthesis | hsa00601 | 2.881 |
| cAMP signaling pathway | hsa04024 | 2.871 |
| Giloma | hsa05214 | 2.803 |
| Retinal metabolism | hsa00830 | 2.803 |
| Long-term potentiation | hsa04720 | 2.717 |
| Pancreatic cancer | hsa05212 | 2.717 |
| Amphetamine addiction | hsa05031 | 2.634 |
| Oxytocin signaling pathway | hsa04921 | 2.606 |
| p53 signaling pathway | hsa04115 | 2.479 |
| Drug metabolism | hsa00982 | 2.479 |
| Glutamatergic synapse | hsa04724 | 2.470 |
| Thyroid cancer | hsa05216 | 2.380 |
| Regulation of actin cytoskeleton | hsa04810 | 2.363 |
| Melanoma | hsa05218 | 2.335 |
| Leukocyte transendothelial migration | hsa04670 | 2.278 |
| Galactose metabolism | hsa00052 | 2.241 |
| Citrate cycle (TCA cycle) | hsa00020 | 2.241 |
| Leishmaniasis | hsa05140 | 2.202 |
| Chronic myeloid leukemia | hsa05220 | 2.202 |
| Metabolism of xenobiotics by cytochrome P450 | hsa00980 | 2.202 |
| Neurotrophin signaling pathway | hsa04722 | 2.189 |
| Adherens junction | hsa04520 | 2.139 |
| Other types of O-glycan biosynthesis | hsa00514 | 2.113 |
| Asthma | hsa05310 | 2.113 |
| Lysosome | hsa04142 | 2.064 |
| Cell cycle | hsa04110 | 2.024 |
| Influenza A | hsa05164 | 2.003 |
| Complement and coagulation cascades | hsa04610 | 1.858 |
| Dopaminergic synapse | hsa04728 | 1.838 |
| Chemical carcinogenesis | hsa05204 | 1.711 |
| Prion diseases | hsa05020 | 1.693 |
| Hypertrophic cardiomyopathy (HCM) | hsa05410 | 1.666 |
| Systemic lupus erythematosus | hsa05322 | 1.641 |
| DNA replication | hsa03030 | 1.606 |
| ErbB signaling pathway | hsa04012 | 1.499 |
| Allograft rejection | hsa05330 | 1.451 |
| Wnt signaling pathway | hsa04310 | 1.443 |
| Prostate cancer | hsa05215 | 1.423 |
| Rheumatoid arthritis | hsa05323 | 1.387 |
| Aldosterone-regulated sodium reabsorption | hsa04960 | 1.381 |
| GnRH signaling pathway | hsa04912 | 1.352 |
| Nicotine addiction | hsa05033 | 1.316 |
| Adrenergic signaling in cardiomyocytes | hsa04261 | 1.295 |
| Viral carcinogenesis | hsa05203 | 1.294 |
| Cytokine-cytokine receptor interaction | hsa04060 | 1.263 |
| Graft-versus-host disease | hsa05332 | 1.254 |
| Pancreatic secretion | hsa04972 | 1.193 |
| Estrogen signaling pathway | hsa04915 | 1.109 |
| Fatty acid degradation | hsa00071 | 1.092 |
| ABC transporters | hsa02010 | 1.092 |
| Jak-STAT signaling pathway | hsa04630 | 1.087 |
| Basal transcription factors | hsa03022 | 1.045 |

**Table S8.** Pathways related to symptomatic HD

| **Pathway** | **KEGG Reference** | **Combined score** |
| --- | --- | --- |
| **INITIAL PATHWAYS** |  |  |
| Viral myocarditis | hsa05416 | 20.171 |
| Small cell lung cancer | hsa05222 | 18.151 |
| Protein digestion and absorption | hsa04974 | 16.658 |
| Transcriptional mis-regulation in cancer | hsa05202 | 15.305 |
| Intestinal immune network for IgA production | hsa04672 | 14.025 |
| Pathways in cancer | hsa05200 | 13.912 |
| Legionellosis | hsa05134 | 10.995 |
| Serotonergic synapse | hsa04726 | 10.833 |
| ECM receptor interaction | hsa04512 | 5.098 |
| TGF-beta signaling pathway | hsa04350 | 0.263 |
| cAMP signaling pathway | hsa04024 | 0.149 |
| **COMPLEMENTARY PATHWAYS** |  |  |
| Leishmaniasis | hsa05140 | 13.698 |
| Thyroid cancer | hsa05216 | 12.304 |
| Nicotinate and nicotinamide metabolism | hsa00760 | 12.304 |
| Asthma | hsa05310 | 11.008 |
| Non-small cell lung cancer | hsa05223 | 10.640 |
| Fructose and mannose metabolism | hsa00051 | 6.743 |
| HTLV-I infection | hsa05166 | 9.656 |
| Fatty acid biosynthesis | hsa00061 | 8.884 |
| Colorectal cancer | hsa05210 | 8.812 |
| Circadian entrainment | hsa04713 | 8.208 |
| Allograft rejection | hsa05330 | 7.760 |
| Retrograde endocannabinoid signaling | hsa04723 | 7.241 |
| Graft-versus-host disease | hsa05332 | 6.783 |
| Bladder cancer | hsa05219 | 6.783 |
| Cell adhesion molecules (CAMs) | hsa04514 | 6.537 |
| Parkinson's disease | hsa05012 | 6.537 |
| Arrhythmogenic right ventricular cardiomyopathy (ARVC) | hsa05412 | 6.267 |
| Type I diabetes mellitus | hsa04940 | 6.226 |
| Proteasome | hsa03050 | 5.972 |
| Cysteine and methionine metabolism | hsa00270 | 5.732 |
| Non-alcoholic fatty liver disease (NAFLD | hsa04932 | 5.689 |
| Hippo signaling pathway | hsa04390 | 5.520 |
| Complement and coagulation cascades | hsa04610 | 5.499 |
| Fatty acid metabolism | hsa01212 | 5.089 |
| Hypertrophic cardiomyopathy (HCM) | hsa05410 | 4.973 |
| Malaria | hsa05144 | 4.897 |
| Glycosaminoglycan degradation | hsa00531 | 4.894 |
| Platelet activation | hsa04611 | 4.830 |
| Cell cycle | hsa04110 | 4.658 |
| Glycosaminoglycan biosynthesis | hsa00532 | 4.502 |
| One carbon pool by folate | hsa00670 | 4.502 |
| Hematopoietic cell lineage | hsa04640 | 4.404 |
| GABAergic synapse | hsa04727 | 4.404 |
| Endometrial cancer | hsa05213 | 4.377 |
| Prostate cancer | hsa05215 | 4.301 |
| Regulation of actin cytoskeleton | hsa04810 | 4.268 |
| Dopaminergic synapse | hsa04728 | 4.268 |
| Autoimmune thyroid disease | hsa05320 | 4.200 |
| Dilated cardiomyopathy | hsa05414 | 4.200 |
| Morphine addiction | hsa05032 | 4.103 |
| Spliceosome | hsa03040 | 3.904 |
| Staphylococcus aureus infection | hsa05150 | 3.796 |
| Regulation of lipolysis in adipocytes | hsa04923 | 3.796 |
| Alcoholism | hsa05034 | 3.795 |
| Ribosome | hsa03010 | 3.708 |
| NOD-like receptor signaling pathway | hsa04621 | 3.667 |
| Biosynthesis of unsaturated fatty acids | hsa01040 | 3.570 |
| Renin-angiotensin system | hsa04614 | 3.570 |
| Glycerolipid metabolism | hsa00561 | 3.427 |
| Wnt signaling pathway | hsa04310 | 3.408 |
| Chemokine signaling pathway | hsa04062 | 3.400 |
| Amoebiasis | hsa05146 | 3.347 |
| AGE-RAGE signaling pathway in diabetic complication | hsa04933 | 3.274 |
| Fatty acid elongation | hsa00062 | 3.099 |
| PI3K-Akt signaling pathway | hsa04151 | 3.080 |
| Longevity regulating pathway | hsa04213 | 2.912 |
| Inflammatory bowel disease (IBD) | hsa05321 | 2.822 |
| Glioma | hsa05214 | 2.822 |
| Shigellosis | hsa05131 | 2.822 |
| Phototransduction | hsa04744 | 2.714 |
| MAPK signaling pathway | hsa04010 | 2.666 |
| Oxytocin signaling pathway | hsa04921 | 2.627 |
| Epithelial cell signaling in Helicobacter pylori infection | hsa05120 | 2.573 |
| Endocytosis | hsa04144 | 2.550 |
| PPAR signaling pathway | hsa03320 | 2.496 |
| Glutamatergic synapse | hsa04724 | 2.489 |
| Rap1 signaling pathway | hsa04015 | 2.478 |
| Adipocytokine signaling pathway | hsa04920 | 2.422 |
| Pentose phosphate pathway | hsa00030 | 2.394 |
| Melanoma | hsa05218 | 2.351 |
| Toxoplasmosis | hsa05145 | 2.296 |
| cGMP-PKG signaling pathway | hsa04022 | 2.283 |
| Galactose metabolism | hsa00052 | 2.254 |
| Alzheimer's disease | hsa05010 | 2.248 |
| Chronic myeloid leukemia | hsa05220 | 2.218 |
| Vascular smooth muscle contraction | hsa04270 | 2.207 |
| Adherens junction | hsa04520 | 2.154 |
| Biosynthesis of amino acids | hsa01230 | 2.154 |
| Pertussis | hsa05133 | 2.093 |
| Purine metabolism | hsa00230 | 1.991 |
| Antigen processing and presentation | hsa04612 | 1.978 |
| Bacterial invasion of epithelial cells | hsa05100 | 1.924 |
| Cardiac muscle contraction | hsa04260 | 1.924 |
| SNARE interactions in vesicular transport | hsa04130 | 1.797 |
| Herpes simplex infection | hsa05168 | 1.742 |
| Hepatitis C | hsa05160 | 1.718 |
| DNA replication | hsa03030 | 1.616 |
| Pentose and glucuronate interconversions | hsa00040 | 1.616 |
| Huntington's disease | hsa05016 | 1.550 |
| Salmonella infection | hsa05132 | 1.550 |
| Gap junction | hsa04540 | 1.471 |
| Focal adhesion | hsa04510 | 1.363 |
| Epstein-Barr virus infection | hsa05169 | 1.363 |
| Hepatitis B | hsa05161 | 1.354 |
| NF-kappa B signaling pathway | hsa04064 | 1.296 |
| Pancreatic secretion | hsa04972 | 1.203 |
| Phagosome | hsa04145 | 1.176 |
| Phosphatidylinositol signaling system | hsa04070 | 1.145 |
| Estrogen signaling pathway | hsa04915 | 1.118 |
| Neuroactive ligand-receptor interaction | hsa04080 | 1.109 |
| Fatty acid degradation | hsa00071 | 1.100 |
| Vasopressin-regulated water reabsorption | hsa04962 | 1.100 |

**Table S9.** OR analysis pathways for pre-symptomatic and symptomatic HD

| **Rank** | **Pathway Name** | **Topology** | **OR value** |
| --- | --- | --- | --- |
| 1 | Ubiquinone and other terpenoid-quinone biosynthesis | 74 | 26.81 |
| 2 | Fatty acid biosynthesis | 78 | 24.75 |
| 3 | RNA degradation | 86 | 17.09 |
| 4 | Necroptosis | 92 | 15.48 |
| 5 | mRNA surveillance pathway | 158 | 10.18 |
| 6 | NOD-like receptor signaling pathway | 354 | 7.95 |
| 7 | Purine metabolism | 124 | 7.39 |
| 8 | Pyrimidine metabolism | 84 | 6.97 |
|  | Ribosome | 110 | 6.84 |
| 9 | Cysteine and methionine metabolism | 192 | 6.33 |
| 10 | C-type lectin receptor signaling pathway | 124 | 5.82 |
| 11 | Thyroid hormone signaling pathway | 138 | 5.36 |
| 12 | Bacterial invasion of epithelial cells | 378 | 5.17 |
| 13 | Phosphatidylinositol signaling system | 611 | 5.06 |
| 14 | Glycosaminoglycan biosynthesis - chondroitin sulfate dermatan sulfate infection | 321 | 5.06 |
| 15 | Ferroptosis | 100 | 4.93 |
| 16 | Alcoholism | 226 | 4.73 |
| 17 | Biosynthesis of unsaturated fatty acids | 80 | 4.46 |
| 18 | Fructose and mannose metabolism | 76 | 3.98 |
| 19 | Synaptic vesicle cycle | 94 | 3.72 |
| 20 | Phototransduction | 76 | 3.66 |
| 21 | Vasopressin-regulated water reabsorption | 94 | 3.52 |
| 22 | One carbon pool by folate | 397 | 3.48 |
|  | Peroxisome |  |  |
|  |  | 98 | 3.32 |
| 23 | Spliceosome |  |  |
| 24 | Fatty acid elongation | 108 | 3.27 |
| 25 | Viral myocarditis | 139 | 3.25 |
| 26 | Natural killer cell mediated cytotoxicity | 177 | 3.19 |
| 27 | Renin-angiotensin system | 106 | 3.17 |
| 28 | Morphine addiction | 129 | 3.17 |
| 29 | Glutathione metabolism | 188 | 3.02 |
| 30 | Notch signaling pathway | 98 | 3.01 |
| 31 | Epithelial cell signaling in Helicobacter pylori infection | 96 | 2.61 |
| 32 | Insulin resistance | 118 | 2.59 |
| 33 | Nicotinate and nicotinamide metabolism | 490 | 2.56 |
| 34 | Cholinergic synapse | 122 | 2.54 |
| 35 | Inositol phosphate metabolism | 729 | 2.51 |
| 36 | Chronic myeloid leukemia | 178 | 2.45 |
| 37 | IL-17 signaling pathway | 106 | 2.35 |
| 38 | Renin secretion |  |  |
|  |  | 190 | 2.35 |
| 39 | Drug metabolism-Cytochrome P450 | 198 | 2.30 |
| 40 | HIF-1 signaling pathway | 92 | 2.29 |
| 41 | Adipocytokine signaling pathway | 276 | 2.82 |
| 42 | Pentose phosphate pathway | 94 | 2.24 |
| 43 | Ribosome biogenesis in eukaryotes | 444 | 2.23 |
| 44 | Drug metabolism-other enzymes | 174 | 2.20 |
| 45 | Cellular senescence | 154 | 2.19 |
| 46 | Ubiquitin mediated proteolysis | 769 | 2.17 |
| 47 | Regulation of lipolysis in adipocytes | 157 | 2.16 |
| 48 | cGMP-PKG signaling pathway | 370 | 2.15 |
| 49 | Long term depression | 112 | 2.03 |
| 50 | Non-small cell lung cancer | 177 | 2.00 |
| 51 | Type II diabetes mellitus | 134 | 1.98 |
| 52 | Circadian entrainment | 208 | 1.97 |
| 53 | Influenza A | 2545 | 1.92 |
| 54 | Transcriptional mis-regulation in cancer | 329 | 1.90 |
| 55 | Oocyte meiosis | 123 | 1.90 |
| 56 | Hepatitis C | 364 | 1.87 |
| 57 | Insulin signaling pathway | 282 | 1.85 |
| 58 | Folate biosynthesis | 108 | 1.82 |
| 59 | Thyroid cancer | 148 | 1.81 |
| 60 | Longevity regulating pathway-multiple species | 168 | 1.77 |
| 61 | Malaria | 301 | 1.77 |
| 62 | Retrograde endocannabinoid signaling | 813 | 1.76 |
| 63 | GABAergic synapse | 423 | 1.75 |
|  |  |  | 1.72 |
| 64 | Relaxin signaling pathway | 183 |  |
|  |  |  | 1.71 |
| 65 | Endocrine resistance | 215 |  |
| 66 | AGE-RAGE signaling in diabetic complications | 137 | 1.67 |
| 67 | Fatty acid degradation | 478 | 1.66 |
| 68 | Kaposi sarcoma associated herpesvirus infection | 493 | 1.66 |
| 69 | Prostate cancer | 603 | 1.65 |
| 70 | VEGF signaling pathway | 104 | 1.64 |
| 71 | AMPK signaling pathway | 347 | 1.62 |
| 72 | Steroid biosynthesis | 656 | 1.62 |
| 73 | Bladder cancer | 284 | 1.62 |
| 74 | Vascular smooth muscle contraction | 189 | 1.60 |
| 75 | Parkinson disease | 436 | 1.59 |
| 76 | Ovarian steroidgensis | 136 | 1.58 |
| 77 | FoxO signaling pathway | 209 | 1.58 |
| 78 | Shigellosis | 199 | 1.53 |
| 80 | Glioma | 187 | 1.50 |
| 81 | Melanoma | 182 | 1.50 |
| 82 | PPAR signaling pathway | 275 | 1.49 |
| 83 | Prolactin signaling pathway | 106 | 1.48 |
| 84 | Cardiac muscle contraction | 477 | 1.48 |
| 85 | Cell adhesion molecules | 568 | 1.46 |
| 86 | Glycerolipid metabolism | 762 | 1.46 |
| 87 | Pancreatic cancer | 159 | 1.45 |
| 88 | Long term potentiation | 297 | 1.40 |
| 89 | Maturity onset diabetes of the young | 66 | 1.39 |
| 90 | Adrenergic signaling in cardiomyocytes | 618 | 1.37 |
| 91 | Selenocompound metabolism | 74 | 1.37 |
| 92 | Riboflavin metabolism | 62 | 1.35 |
| 93 | Oxytocin signaling pathway | 260 | 1.34 |
| 94 | Arachidonic acid metabolism | 245 | 1.33 |
| 95 | Small cell lung cancer | 592 | 1.31 |
| 96 | Osteoclast differentiation | 92 | 1.29 |
| 97 | Endocytosis | 2464 | 1.29 |
| 98 | Alzheimer disease | 2218 | 1.28 |
| 99 | TNF signaling pathway | 187 | 1.27 |
| 100 | Phospholipase D signaling pathway | 1792 | 1.27 |
| 101 | Platelet activation | 269 | 1.26 |
| 102 | Th17 cell differentiation | 1158 | 1.25 |
| 103 | Metabolic pathways | 16471 | 1.24 |
| 104 | Homologous recombination | 62 | 1.23 |
| 105 | Taurine and hypotaurine metabolism | 78 | 1.23 |
| 106 | Glycosylphospatidylinositol (GPI)- anchor biosynthesis | 86 | 1.23 |
| 107 | MicroRNAs in cancer | 176 | 1.23 |
| 108 | Fc gamma R-mediated phagocytosis | 129 | 1.21 |
| 109 | Progesterone mediated oocyte maturation | 244 | 1.21 |
| 110 | Glutamatergic synapse | 960 | 1.21 |
| 111 | Melanogenesis | 137 | 1.20 |
| 112 | Oxidative phosphorylation | 14014 | 1.19 |
| 113 | Ras signaling pathway | 623 | 1.18 |
| 114 | D-glutamine and D-glutamate metabolism | 78 | 1.18 |
| 115 | Glycosphingolipid biosynthesis - ganglio series | 80 | 1.75 |
| 116 | Axon guidance | 745 | 1.72 |
| 117 | Lysine degradation | 72 | 1.70 |
| 118 | Vibrio cholerae infection | 76 | 1.16 |
| 119 | Phenylalanine metabolism | 90 | 1.15 |
| 120 | Non-alcoholic fatty liver disease (NAFLD) | 9031 | 1.14 |
| 121 | Biotin metabolism | 88 | 1.13 |
| 122 | Apoptosis | 6522 | 1.13 |
| 123 | D-Arginine and D-ornithine metabolism | 86 | 1.11 |
| 124 | Glycolysis Gluconeogenesis | 156 | 1.11 |
| 125 | Hepatocellular carcinoma | 183 | 1.10 |
| 126 | RNA transport | 1734 | 1.10 |
| 127 | Histidine metabolism | 112 | 1.08 |
| 128 | beta-Alanine metabolism | 76 | 1.08 |
| 129 | Calcium signaling pathway | 7358 | 1.07 |
| 130 | Apelin signaling pathway | 240 | 1.07 |
| 131 | Primary immunodeficiency | 84 | 1.07 |
| 132 | Intestinal immune network for IgA production | 334 | 1.06 |
| 133 | 2-Oxocarboxylic acid metabolism | 92 | 1.06 |
| 134 | Thermogenesis | 2487 | 1.05 |
| 135 | Thiamine metabolism | 76 | 1.05 |
| 136 | Dopaminergic synapse | 1473 | 1.05 |
| 137 | Fluid shear stress and atherosclerosis | 104 | 1.05 |
| 138 | Regulation of actin cytoskeleton | 1988 | 1.04 |
| 139 | Human cytomegalovirus infection | 392 | 1.04 |
| 140 | Tight junction | 1558 | 1.04 |
| 141 | Valine, leucine and isoleucine biosynthesis | 82 | 1.02 |
| 142 | Tyrosine metabolism | 92 | 1.02 |
| 143 | MAPK signaling pathway | 10784 | 1.01 |
| 144 | Glycerophospholipid metabolism | 475 | 1.01 |
| 145 | Human immunodeficiency virus 1 infection | 373 | 1.00 |
| 146 | Tryptophan metabolism | 88 | 1.00 |
| 147 | Mucin type O-glycan biosynthesis | 90 | 1.00 |

**Table S10.** OR analysis pathways for pre-symptomatic HD

| **Rank** | **Pathway Name** | **Topology** | **OR value** |
| --- | --- | --- | --- |
| 1 | Hippo signaling pathway-Multiple species | 14543 | 9.75 |
| 2 | Glycosaminoglycan biosynthesis - heparan sulfate heparin | 14929 | 7.70 |
| 3 | Proteasome | 14682 | 7.22 |
| 4 | Herpes simplex virus 1 infection | 43873 | 6.12 |
| 5 | TGF-beta signaling pathway | 14755 | 6.08 |
| 6 | Mannose type O-glycan biosynthesis | 14612 | 5.34 |
| 7 | Hippo signaling pathway | 29720 | 5.32 |
| 8 | Tight junction | 17923 | 3.95 |
| 9 | Cell cycle | 26902 | 3.88 |
| 10 | Neurotrophin signaling pathway | 14953 | 3.36 |
| 11 | Glycosphingolipid biosynthesis - lacto and neolacto series | 14975 | 3.28 |
| 12 | Signaling pathways regulating pluripotency of stem cells | 14931 | 3.00 |
| 13 | Endocytosis | 37479 | 2.97 |
| 14 | JAK-STAT signaling pathway | 47463 | 2.88 |
| 15 | Neuroactive ligand-receptor interaction | 14747 | 2.88 |
|  |  |  |  |
| 16 | Fanconi anemia pathway | 29108 | 2.79 |
| 17 | Mitophagy - animal | 15090 | 2.71 |
| 18 | Cholesterol metabolism | 14877 | 2.44 |
| 19 | Inflammatory bowel disease (IBD) | 14834 | 2.42 |
| 20 | Ubiquitin mediated proteolysis | 15012 | 2.31 |
| 21 | Basal transcription factors | 29670 | 2.29 |
| 22 | Axon guidance | 14838 | 2.27 |
| 23 | Amoebiasis | 14877 | 2.25 |
| 24 | Inositol phosphate metabolism | 14861 | 2.21 |
| 25 | ECM-receptor interaction | 43800 | 2.20 |
| 26 | African trypanosomiasis | 14862 | 2.19 |
| 27 | Epstein-Barr virus infection | 54034 | 2.15 |
| 28 | Wnt signaling pathway | 92442 | 2.13 |
| 29 | Serotonergic synapse | 14667 | 2.14 |
| 30 | Estrogen signaling pathway | 27692 | 2.12 |
| 31 | Arrhythmogenic right ventricular cardiomyopathy (ARVC) | 14607 | 2.11 |
| 32 | Mineral absorption | 15044 | 2.08 |
| 33 | Adherens junction | 14867 | 2.01 |
| 34 | Antifolate resistance | 14624 | 1.98 |
| 35 | ErbB signaling pathway | 14822 | 1.98 |
| 36 | Longevity regulating pathway | 1500 | 1.97 |
| 37 | Type I diabetes mellitus | 14816 | 1.96 |
| 38 | Steroid biosynthesis | 15185 | 1.95 |
| 39 | Phosphatidylinositol signaling system | 14434 | 1.91 |
| 40 | Colorectal cancer | 57818 | 1.88 |
| 41 | Glycosaminoglycan degradation | 19718 | 1.87 |
| 42 | Bile secretion | 24692 | 1.85 |
| 43 | Small cell lung cancer | 14726 | 1.82 |
| 44 | RNA transport | 44420 | 1.76 |
| 45 | Viral carcinogenesis | 46195 | 1.75 |
| 46 | Protein export | 15042 | 1.74 |
| 47 | Phospholipase D signaling pathway | 46703 | 1.73 |
| 48 | Human papillomavirus infection | 87482 | 1.72 |
| 49 | Dilated cardiomyopathy (DCM) | 14874 | 1.72 |
| 50 | Mismatch repair | 43631 | 1.71 |
| 51 | Cytokine-cytokine receptor interaction | 33758 | 1.71 |
| 52 | Spliceosome | 14930 | 1.71 |
| 53 | Focal adhesion | 112460 | 1.67 |
| 54 | Cushing syndrome | 22022 | 1.66 |
| 55 | Toll-like receptor signaling pathway | 104478 | 1.61 |
|  |  |  | 1.59 |
| 56 | Autophagy - animal | 36908 |  |
|  |  |  | 1.57 |
| 57 | Gastric acid secretion | 14832 |  |
| 58 | Apoptosis - multiple species | 15252 | 1.56 |
| 59 | Cocaine addiction | 14704 | 1.53 |
| 60 | Hematopoietic cell lineage | 14587 | 1.53 |
| 61 | Th1 and Th2 cell differentiation | 15083 | 1.53 |
| 62 | Pathways in cancer | 457037 | 1.50 |
| 63 | Gap junction | 29679 | 1.51 |
| 64 | Nicotinate and nicotinamide metabolism | 14943 | 1.48 |
|  |  |  | 1.48 |
| 65 | Arginine biosynthesis | 14970 |  |
| 66 | Other glycan degradation | 14962 | 1.47 |
| 67 | Platinum drug resistance | 14913 | 1.46 |
| 68 | Hypertrophic cardiomyopathy (HCM) | 14859 | 1.45 |
| 69 | Phagosome | 21687 | 1.44 |
| 70 | RIG-I-like receptor signaling pathway | 15737 | 1.41 |
| 71 | Salivary secretion | 28716 | 1.40 |
| 72 | Renal cell carcinoma | 14546 | 1.40 |
| 73 | Lysosome | 16171 | 1.40 |
| 74 | N-Glycan biosynthesis | 23550 | 1.36 |
| 75 | Toxoplasmosis | 15178 | 1.34 |
| 76 | Complement and coagulation cascades | 29917 | 1.31 |
| 77 | GnRH signaling pathway | 25177 | 1.26 |
| 78 | Inflammatory mediator regulation of TRP channels | 14667 | 1.25 |
| 79 | B cell receptor signaling pathway | 14744 | 1.24 |
| 80 | Starch and sucrose metabolism | 14802 | 1.23 |
| 81 | Gastric cancer |  |  |
|  | Th17 cell differentiation | 14686 | 1.20 |
| 82 |  |  |  |
|  | Galactose metabolism | 14882 | 1.20 |
| 83 |  |  |  |
| 84 | Taste transduction | 14863 | 1.20 |
| 85 | Protein digestion and absorption | 29439 | 1.18 |
| 86 | Amphetamine addiction | 14924 | 1.16 |
| 87 | mTOR signaling pathway | 38118 | 1.14 |
| 88 | Ras signaling pathway | 25148 | 1.12 |
| 89 | Protein processing in endoplasmic reticulum | 29872 | 1.12 |
| 90 | Systemic lupus erythematosus | 16064 | 1.11 |
| 91 | Nicotine addiction | 14952 | 1.10 |
| 92 | Bacterial invasion of epithelial cells | 15585 | 1.09 |
| 93 | Dopaminergic synapse | 61069 | 1.09 |
| 94 | Carbohydrate digestion and absorption | 14874 | 1.08 |
| 95 | Ascorbate and aldarate metabolism | 14733 | 1.07 |
| 96 | Prion diseases | 14508 | 1.07 |
| 97 | Leishmaniasis | 30944 | 1.06 |
| 98 | Influenza A | 108816 | 1.05 |
| 99 | MAPK signaling pathway | 472571 | 1.03 |
| 100 | Intestinal immune network for IgA production | 14648 | 1.03 |
| 101 | Leukocyte transendothelial migration | 26951 | 1.03 |
| 102 | Parkinson disease | 19229 | 1.02 |
| 103 | NOD-like receptor signaling pathway | 15679 | 1.02 |
| 104 | Huntington disease | 29634 | 1.01 |
| 105 | Regulation of actin cytoskeleton | 88624 | 1.04 |
| 106 | Transcriptional misregulation in cancer | 14859 | 1.00 |

**Table S11.** OR analysis pathways for symptomatic HD

| **Rank** | **Pathway Name** | **Topology** | **OR value** |
| --- | --- | --- | --- |
| 1 | Phosphatidylinositol signaling system | 14434 | 9.72 |
| 2 | NOD-like receptor signaling pathway | 15679 | 8.13 |
| 3 | Ubiquinone and other terpenoid-quinone biosynthesis | 14648 | 6.12 |
| 4 | Fatty acid biosynthesis | 15001 | 5.82 |
| 5 | Bacterial invasion of epithelial cells | 15585 | 5.68 |
| 6 | Inositol phosphate metabolism | 14861 | 5.58 |
| 7 | Spliceosome | 14930 | 5.56 |
| 8 | Hippo signaling pathway - multiple species | 14543 | 5.25 |
| 9 | Proteasome | 14682 | 5.10 |
| 10 | Ubiquitin mediated proteolysis | 15012 | 5.05 |
| 11 | Glycosaminoglycan biosynthesis - chondroitin sulfate dermatan sulfate | 14689 | 5.00 |
| 12 | RNA degradation | 14837 | 4.48 |
| 13 | Necroptosis |  | 4.41 |
| 14 | Herpes simplex virus 1 infection | 43873 | 4.19 |
| 15 | Tight junction | 17923 | 4.11 |
| 16 | Endocytosis | 37479 | 3.88 |
| 17 | Nicotinate and nicotinamide metabolism | 14943 | 3.81 |
| 18 | Cell Cycle | 26902 | 3.71 |
| 19 | TGF-beta signaling pathway | 14755 | 3.57 |
| 20 | Alcoholism | 15189 | 3.18 |
| 21 | Steroid biosynthesis | 15185 | 3.17 |
| 22 | One carbon pool by folate | 19968 | 3.13 |
| 23 | Hippo signaling pathway | 29720 | 3.12 |
| 24 | Purine metabolism | 14852 | 2.79 |
| 25 | Axon guidance | 14838 | 2.66 |
| 26 | mRNA surveillance pathway | 29448 | 2.46 |
| 27 | Small cell lung cancer | 14726 | 2.39 |
| 28 | Ribosome | 14830 | 2.29 |
| 29 | Thyroid hormone signaling pathway | 14924 | 2.24 |
| 30 | Phospholipase D signaling pathway | 46703 | 2.21 |
| 31 | C-type lectin receptor signaling pathway | 14797 | 2.21 |
| 32 | Cholesterol metabolism | 14877 | 2.04 |
| 33 | Influenza A | 108816 | 2.04 |
| 34 | RNA transport | 44420 | 1.98 |
| 35 | Transcriptional misregulation in cancer | 14859 | 1.95 |
| 36 | Pyrimidine metabolism | 14950 | 1.77 |
| 37 | Neuroactive ligand-receptor interaction | 14747 | 1.75 |
| 38 | Longevity regulating pathway | 15000 | 1.75 |
| 39 | Epstein-Barr virus infection | 54034 | 1.74 |
| 40 | Estrogen signaling pathway | 27692 | 1.74 |
| 41 | JAK-STAT signaling pathway | 47463 | 1.67 |
| 42 | Arrhythmogenic right ventricular cardiomyopathy (ARVC) | 14607 | 1.66 |
| 43 | Parkinson disease | 19229 | 1.63 |
| 44 | Malaria | 15023 | 1.61 |
| 45 | Wnt signaling pathway | 92442 | 1.55 |
| 46 | Toll-like receptor signaling pathway | 104478 | 1.55 |
| 47 | Glycosaminoglycan degradation | 19718 | 1.54 |
| 48 | Th17 cell differentiation | 43382 | 1.53 |
| 49 | Ferroptosis | 14781 | 1.51 |
| 50 | Ribosome biogenesis in eukaryotes | 29831 | 1.50 |
| 51 | Dilated cardiomyopathy (DCM) | 14874 | 1.48 |
| 52 | Serotonergic synapse | 14667 | 1.44 |
| 53 | Focal adhesion | 112460 | 1.42 |
| 54 | Bladder cancer | 14765 | 1.41 |
| 55 | Neurotrophin signaling pathway | 14953 | 1.41 |
| 56 | Drug metabolism - cytochrome P450 | 14747 | 1.40 |
| 57 | Viral myocarditis | 14940 | 1.36 |
| 58 | Chronic myeloid leukemia | 14680 | 1.34 |
| 59 | Ras signaling pathway | 25148 | 1.32 |
| 60 | Hypertrophic cardiomyopathy (HCM) | 14859 | 1.28 |
| 61 | Cushing syndrome | 22022 | 1.25 |
| 62 | ECM-receptor interaction | 43800 | 1.24 |
| 63 | Human papillomavirus infection | 87482 | 1.24 |
| 64 | Long-term potentiation | 15239 | 1.24 |
| 65 | Circadian entrainment | 14955 | 1.24 |
| 66 | Morphine addiction | 14895 | 1.24 |
| 67 | Glycerolipid metabolism | 41962 | 1.20 |
| 68 | Pathways in cancer | 457037 | 1.19 |
| 69 | Drug metabolism - other enzymes | 14822 | 1.17 |
| 70 | cGMP-PKG signaling pathway | 30601 | 1.17 |
| 71 | Mannose type O-glycan biosynthesis | 14612 | 1.17 |
| 72 | Dopaminergic synapse | 61069 | 1.15 |
| 73 | Complement and coagulation cascades | 29917 | 1.13 |
| 74 | Endocrine resistance | 14798 | 1.12 |
| 75 | Amphetamine addiction | 14924 | 1.12 |
| 76 | Fatty acid degradation | 32022 | 1.12 |
| 77 | Apoptosis | 301799 | 1.10 |
| 78 | Intestinal immune network for IgA production | 14648 | 1.10 |
| 79 | Biosynthesis of unsaturated fatty acids | 14680 | 1.10 |
| 80 | Phagosome | 21687 | 1.09 |
| 81 | Synaptic vesicle cycle | 14770 | 1.09 |
| 82 | Cardiac muscle contraction | 29554 | 1.08 |
| 83 | Oxytocin signaling pathway | 14739 | 1.07 |
| 84 | Non-small cell lung cancer | 14976 | 1.07 |
| 85 | Adipocytokine signaling pathway | 26676 | 1.06 |
| 86 | Fatty acid elongation | 15007 | 1.06 |
| 87 | Gap junction | 29679 | 1.06 |
| 88 | Glutamatergic synapse | 49419 | 1.06 |
| 89 | Regulation of actin cytoskeleton | 88624 | 1.06 |
| 90 | MAPK signaling pathway | 472571 | 1.05 |
| 91 | Regulation of lipolysis in adipocytes | 14604 | 1.05 |
| 92 | Calcium signaling pathway | 342037 | 1.04 |
| 93 | Prostate cancer | 43119 | 1.04 |
| 94 | PPAR signaling pathway | 17788 | 1.04 |
| 95 | GABAergic synapse | 32186 | 1.04 |
| 96 | Renin-angiotensin system | 14790 | 1.02 |
| 97 | Platelet activation | 14937 |  |
| 98 | Cellular senescence | 14906 | 1.02 |
| 99 | Protein processing in endoplasmic reticulum | 29872 | 1.01 |
| 100 | Peroxisome | 14911 | 1.00 |
| 101 | Leishmaniasis | 30944 | 1.00 |

**Table S12.** Exclusive metabolites pathways and the number of metabolites related to pre-symptomatic HD.

| **Pathway** | **KEGG id** | **No. of metabolites** |
| --- | --- | --- |
| **Pre-symptomatic HD** | | |
| Glycosaminoglycan biosynthesis | hsa00532 | 5 |
| Ascorbate and aldarate metabolism | hsa00053 | 49 |
| African trypanosomiasis | hsa05143 | 8 |
| Thyroid hormone synthesis | hsa04918 | 21 |
| Porphyrin and chlorophyll metabolism | hsa00860 | 142 |
| Gastric acid secretion | hsa04971 | 14 |
| Carbohydrate digestion and absorption | hsa04973 | 27 |
| Drug metabolism | hsa00983 | 52 |
| Other glycan degradation | hsa00511 | 0 |
| Cocaine addiction | hsa05030 | 7 |
| Insulin secretion | hsa04911 | 12 |
| Mineral absorption | hsa04978 | 29 |
| Steroid biosynthesis | hsa00100 | 58 |
| Arginine biosynthesis | hsa00220 | 23 |
| Protein export | hsa03060 | 0 |
| Proximal tubule bicarbonate reclamation | hsa04964 | 17 |
| Steroid hormone biosynthesis | hsa00140 | 99 |
| Glycosphingolipid biosynthesis - lacto and neolacto series | hsa00601 | 0 |
| Retinol metabolism | hsa00830 | 25 |

**Table S13.** Exclusive pathways and the number of metabolites related to symptomatic HD.

| **Pathway** | **KEGG id** | **No. of metabolites** |
| --- | --- | --- |
| **Symptomatic HD** | | |
| Nicotinate and nicotinamide metabolism | hsa00760 | 55 |
| Fructose and mannose metabolism | hsa00051 | 54 |
| Fatty acid biosynthesis | hsa00061 | 58 |
| Cysteine and methionine metabolism | hsa00270 | 63 |
| Ribosome | hsa03010 | 0 |
| Biosynthesis of unsaturated fatty acids | hsa01040 | 74 |
| Renin-angiotensin system | hsa04614 | 11 |
| Glycerolipid metabolism | hsa00561 | 38 |
| Shigellosis | hsa05131 | 16 |
| Phototransduction | hsa04744 | 8 |
| Epithelial cell signaling in Helicobacter pylori infection | hsa05120 | 5 |
| Adipocytokine signaling pathway | hsa04920 | 7 |
| Pertussis | hsa05133 | 10 |
| SNARE interactions in vesicular transport | hsa0430 | 0 |
| Hepatitis C | hsa05160 | 2 |
| NF-kappa B signaling pathway | hsa04964 | 3 |
| Vasopressin-regulated water reabsorption | hsa04962 | 2 |
| Type II diabetes mellitus | hsa04930 | 6 |
| Notch signaling pathway | hsa04330 | 0 |
| Pathogenic Escherichia coli infection | hsa05130 | 3 |
| Long-term depression | hsa04730 | 9 |
| VEGF signaling pathway | hsa04370 | 6 |
| Arachidonic acid metabolism | hsa00590 | 75 |
| Natural killer cell mediated cytotoxicity | hsa04650 | 4 |
| Renin secretion | hsa04924 | 17 |
| Glycolysis/Gluconeogenesis | hsa00010 | 31 |
| RNA degradation | hsa00010 | 0 |
| Peroxisome | hsa03018 | 0 |
| mRNA surveillance | hsa03015 | 0 |
| Fc gamma R mediated phagocytosis | hsa04666 | 8 |
| Glycerophospholipid metabolism | hsa00564 | 52 |
| HIF-1 signaling pathway | hsa04066 | 15 |
| Toll-like receptor signaling pathway | hsa04620 | 0 |
| Insulin resistance | hsa04931 | 19 |
| Insulin signaling pathway | hsa04910 | 4 |

**Table S14.** Metabolites exclusive to pre-symptomatic HD


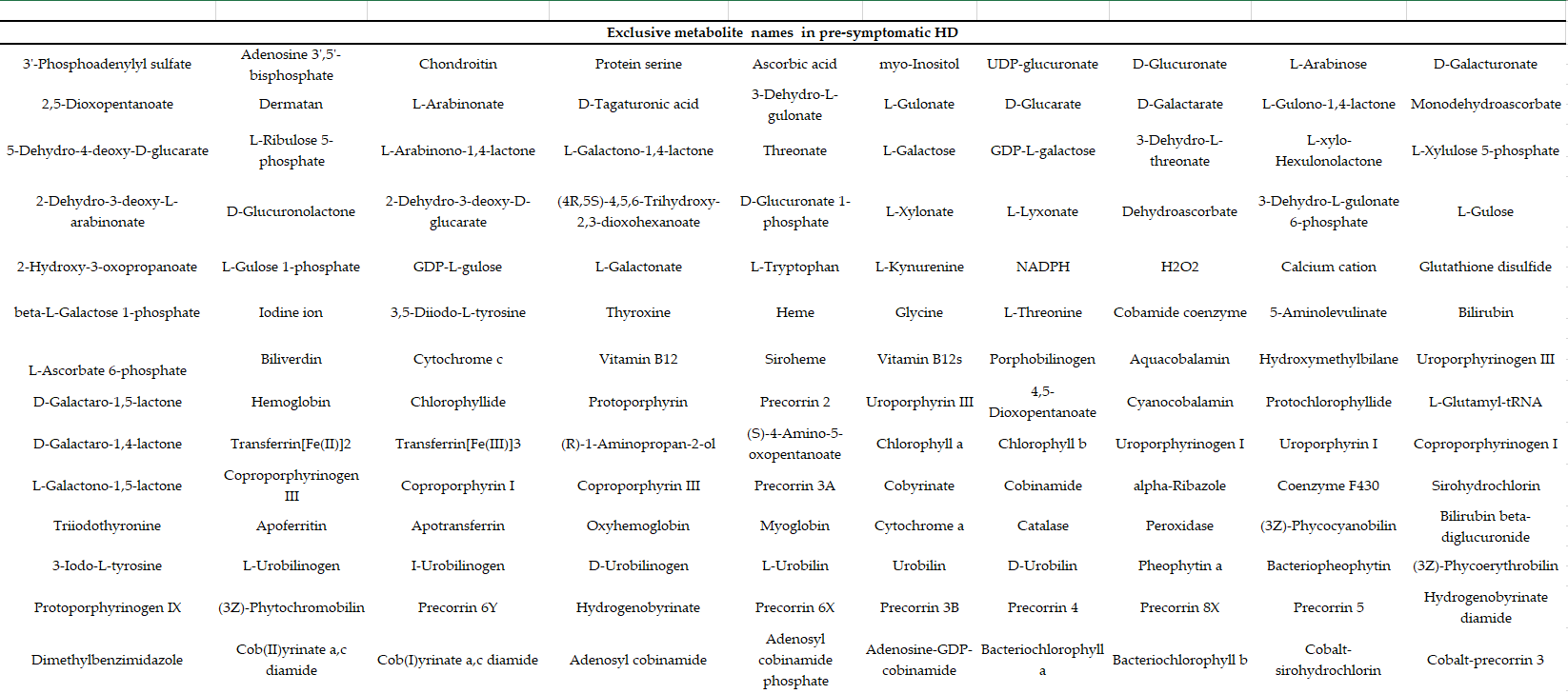


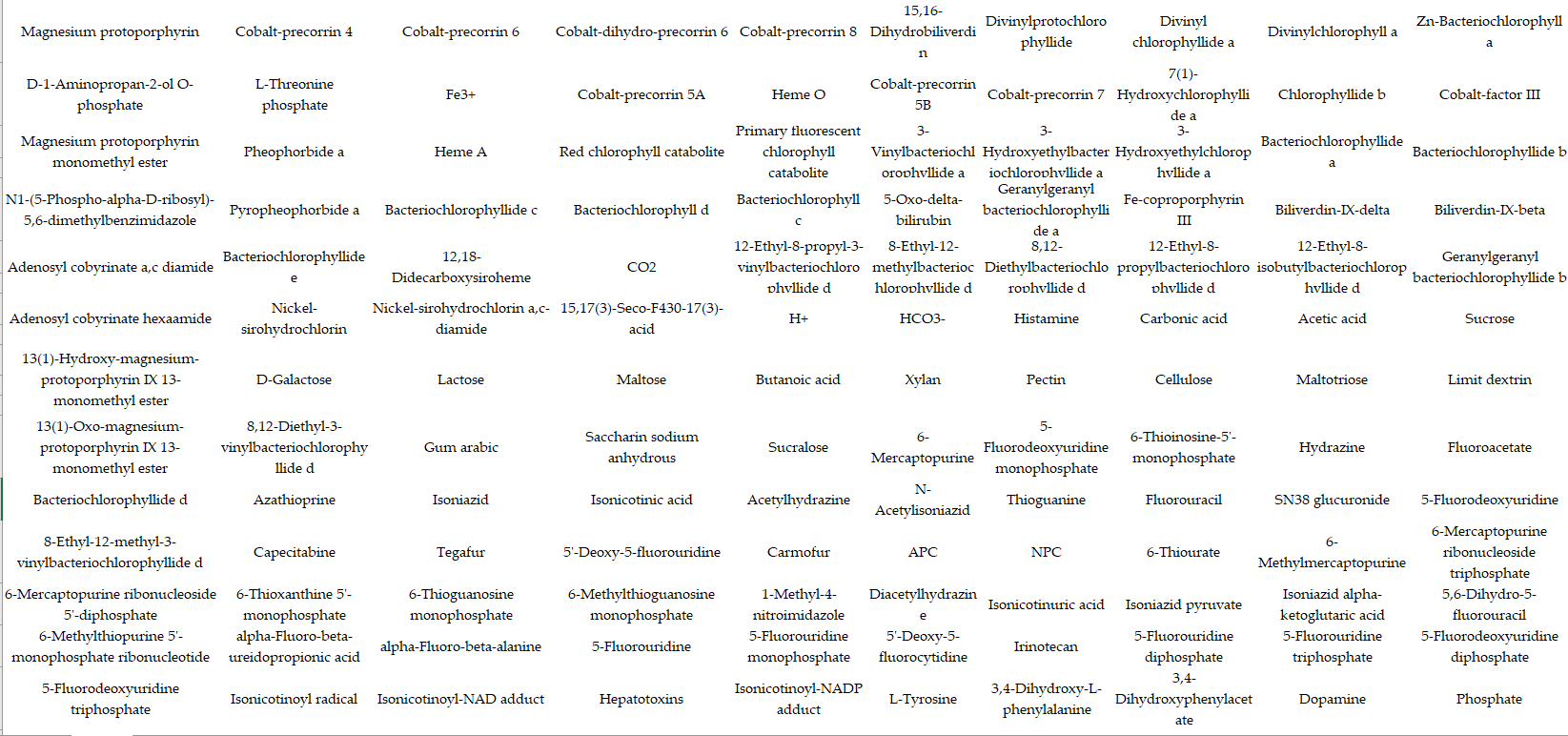


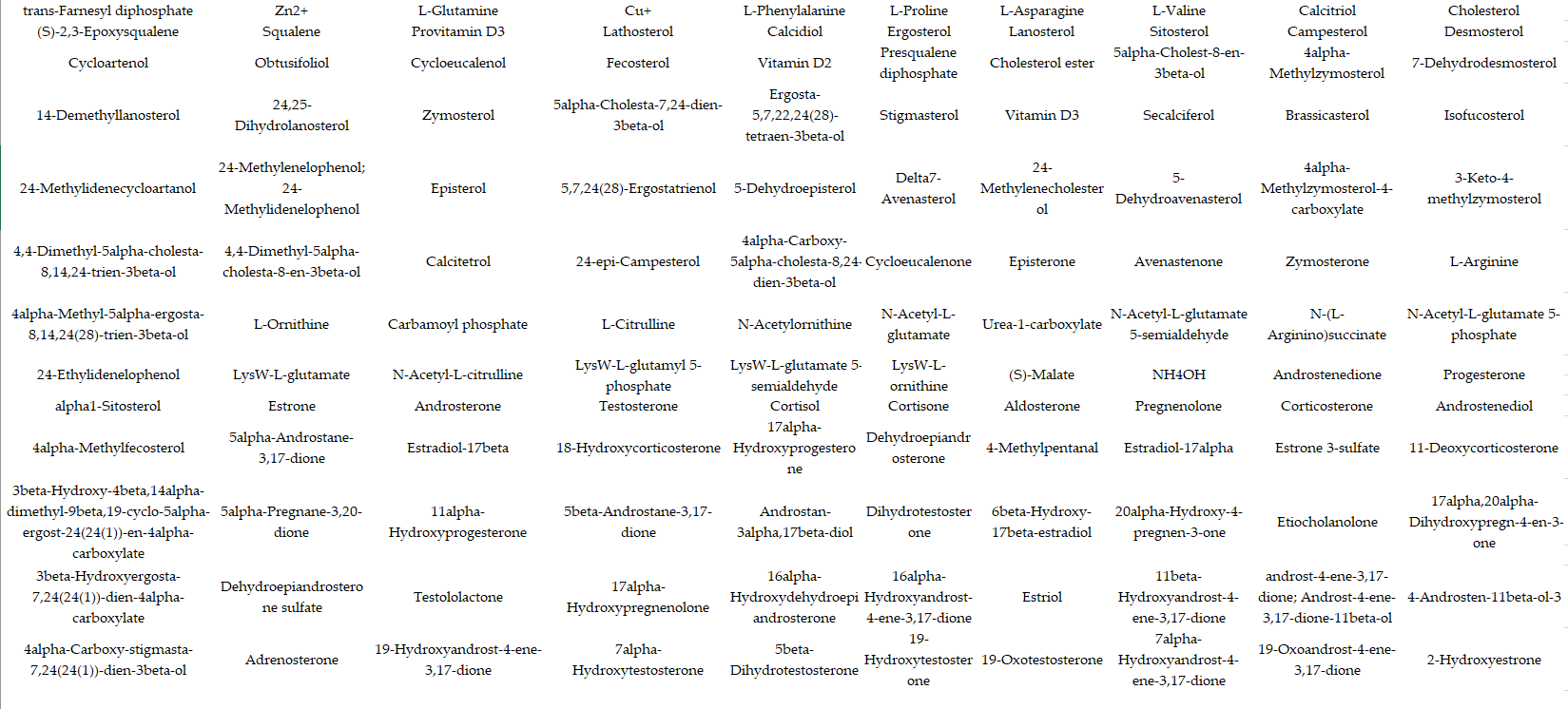


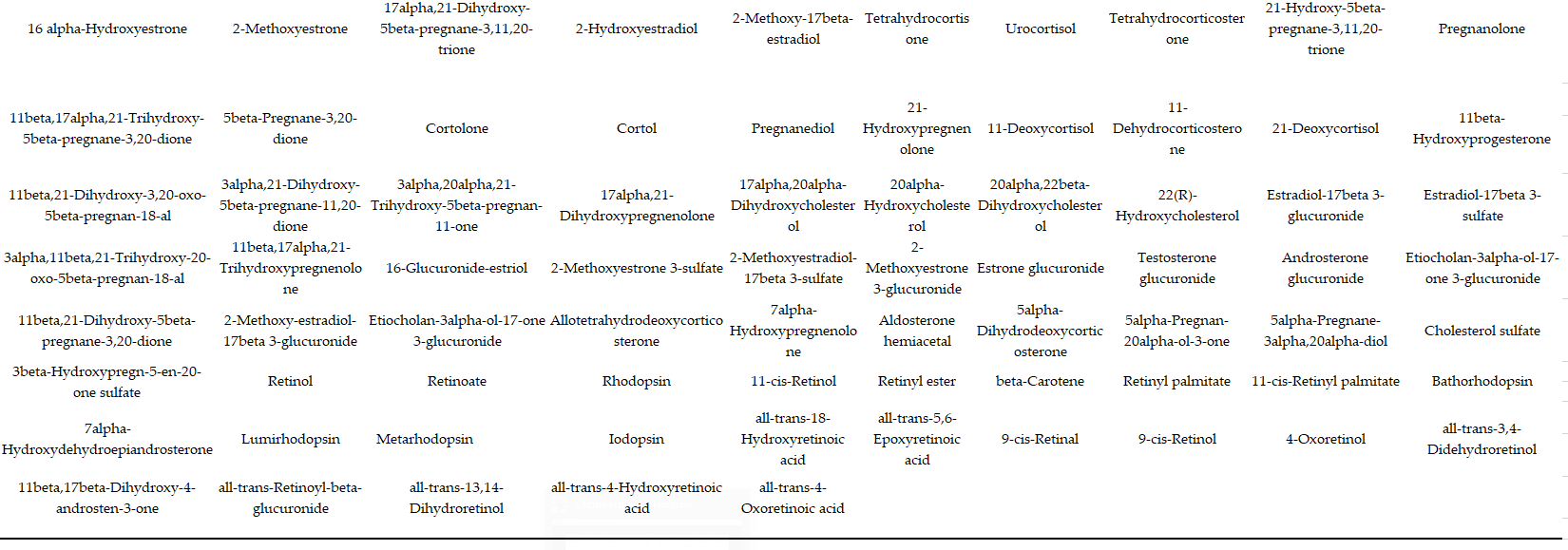


**Table S15.** Metabolites exclusive to symptomatic HD


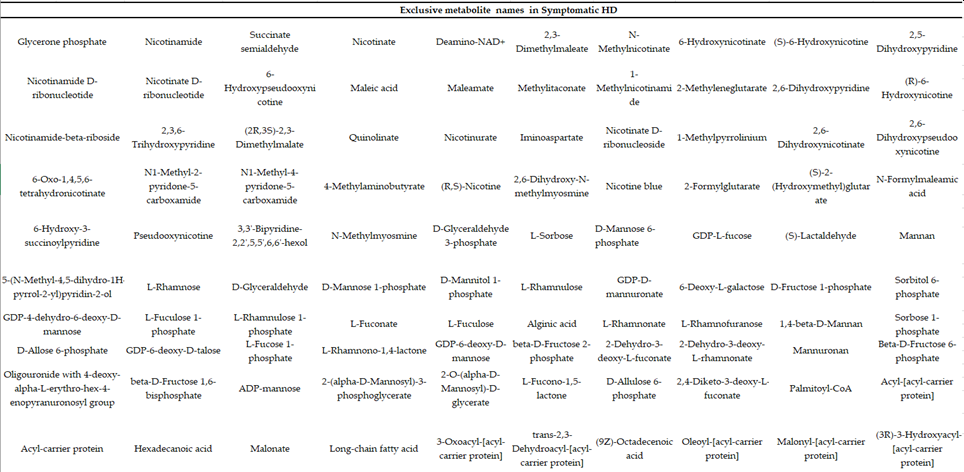


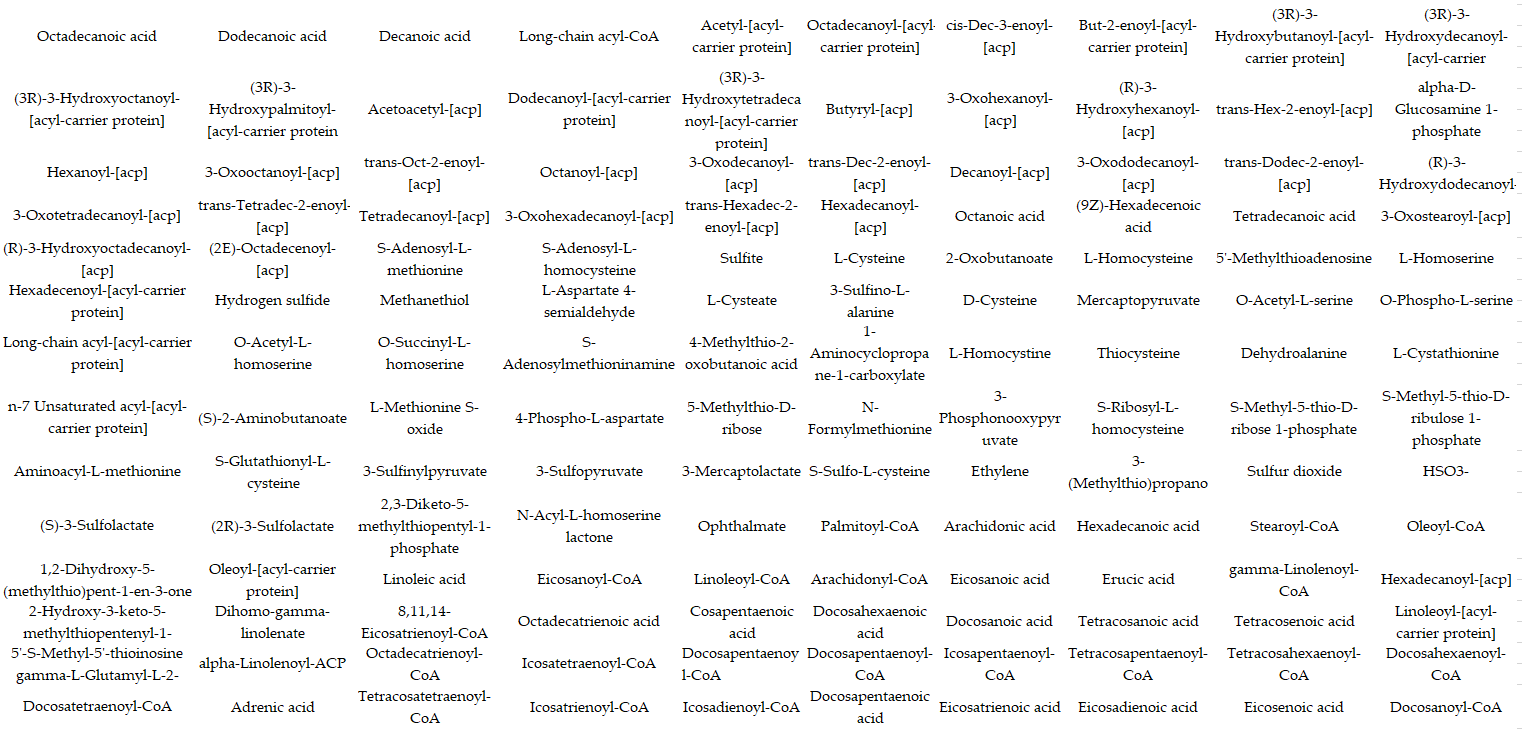


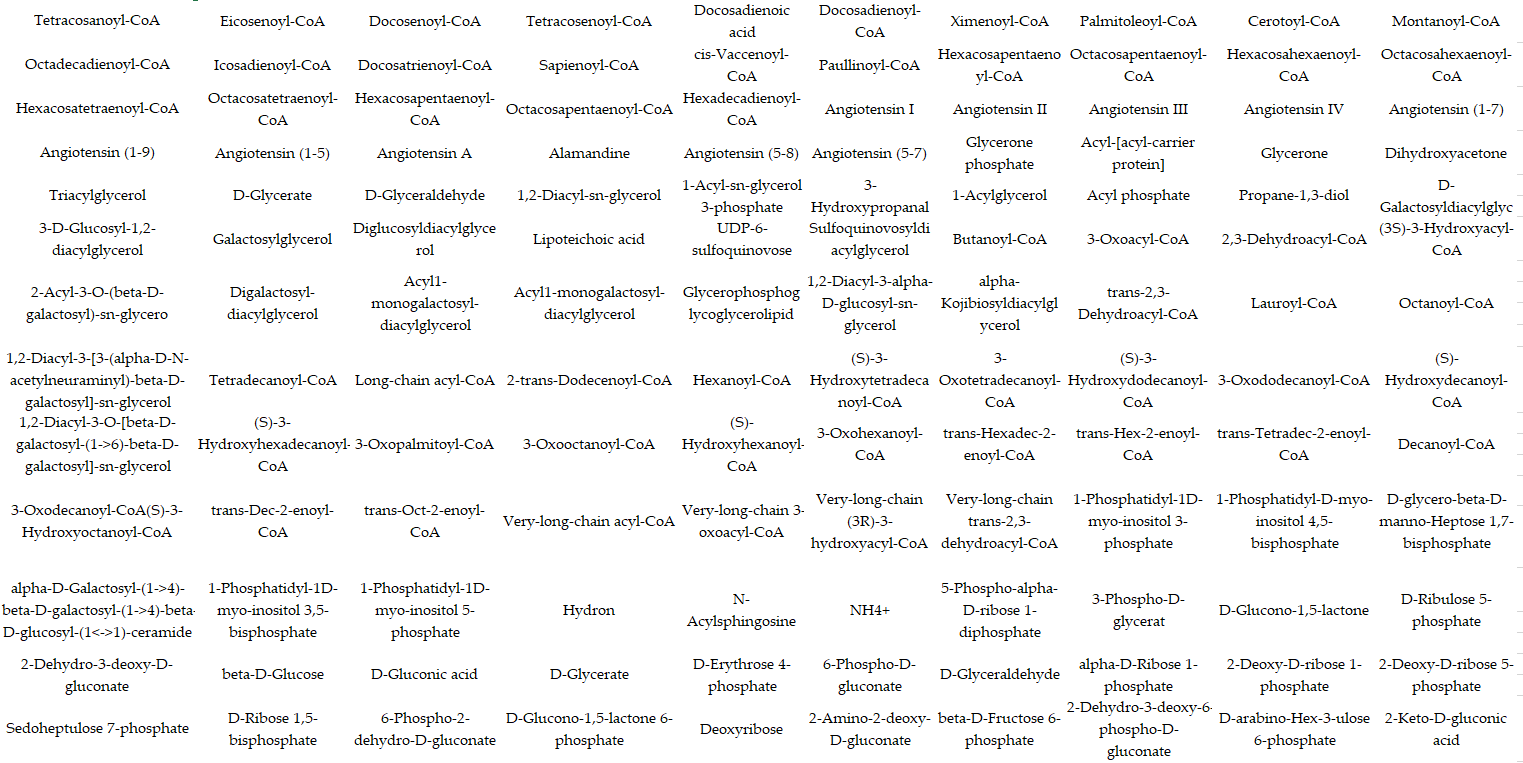


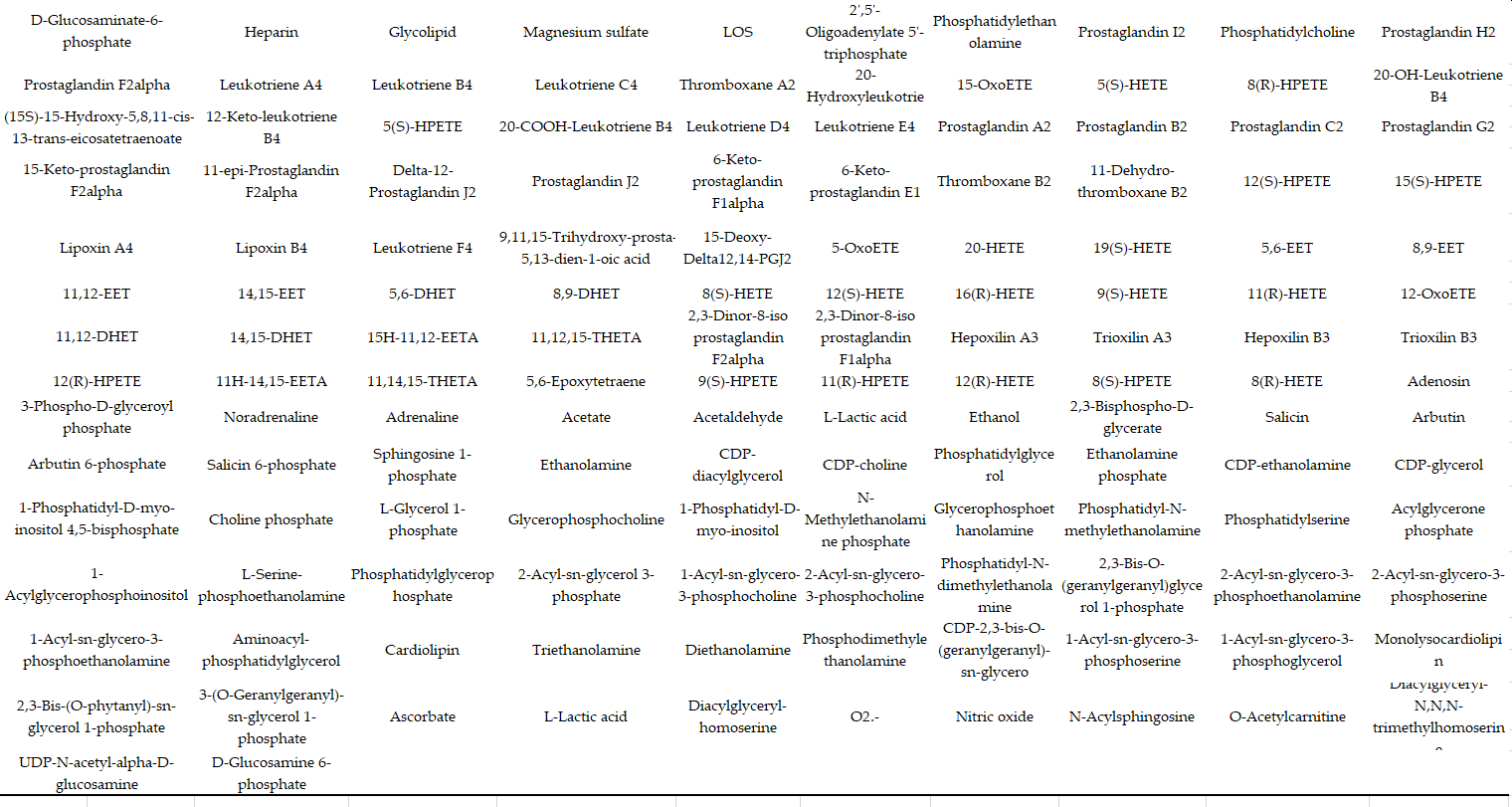


**Table S16.** Exclusive pathways and their respective metabolites related to pre-symptomatic HD

| **Pathway** | **Metabolite** |
| --- | --- |
| Glycosaminoglycan biosynthesis - heparan sulfate / heparin | 3'-Phosphoadenylyl sulfate |
|  | Adenosine 3',5'-bisphosphate |
|  | Chondroitin |
|  | Dermatan |
|  | Protein serine |
| Ascorbate and aldarate metabolism | Ascorbic acid |
|  | myo-Inositol |
|  | UDP-glucuronate |
|  | D-Glucuronate |
|  | L-Arabinose |
|  | D-Galacturonate |
|  | 2,5-Dioxopentanoate |
|  | L-Arabinonate |
|  | D-Tagaturonic acid |
|  | 3-Dehydro-L-gulonate |
|  | 5-Dehydro-4-deoxy-D-glucarate |
|  | 2-Dehydro-3-deoxy-L-arabinonate |
|  | L-Gulonate |
|  | D-Glucarate |
|  | D-Galactarate |
|  | L-Gulono-1,4-lactone |
|  | Monodehydroascorbate |
|  | L-Ribulose 5-phosphate |
|  | L-Arabinono-1,4-lactone |
|  | L-Galactono-1,4-lactone |
|  | 2-Hydroxy-3-oxopropanoate |
|  | Threonate |
|  | L-Galactose |
|  | GDP-L-galactose |
|  | D-Glucuronolactone |
|  | 3-Dehydro-L-threonate |
|  | L-xylo-Hexulonolactone |
|  | L-Xylulose 5-phosphate |
|  | 2-Dehydro-3-deoxy-D-glucarate |
|  | (4R,5S)-4,5,6-Trihydroxy-2,3-dioxohexanoate |
|  | D-Glucuronate 1-phosphate |
|  | L-Xylonate |
|  | L-Lyxonate |
|  | Dehydroascorbate |
|  | 3-Dehydro-L-gulonate 6-phosphate |
|  | L-Gulose |
|  | L-Gulose 1-phosphate |
|  | GDP-L-gulose |
|  | beta-L-Galactose 1-phosphate |
|  | L-Galactonate |
|  | L-Ascorbate 6-phosphate |
|  | D-Galactaro-1,5-lactone |
|  | D-Galactaro-1,4-lactone |
|  | L-Galactono-1,5-lactone |
|  |  |
| **African trypanosomiasis** | L-Tryptophan |
|  | L-Kynurenine |
|  |  |
| **Thyroid hormone synthesis** | NADPH |
|  | H2O2 |
|  | Glutathione disulfide |
|  | I^-^ |
|  | 3,5-Diiodo-L-tyrosine |
|  | Thyroxine |
|  | Triiodothyronine |
|  | 3-Iodo-L-tyrosine |
|  |  |
| Porphyrin and chlorophyll metabolism | Heme |
|  | Glycine |
|  | L-Threonine |
|  | Cobamide coenzyme |
|  | 5-Aminolevulinate |
|  | Bilirubin |
|  | Biliverdin |
|  | Cytochrome c |
|  | Vitamin B12 |
|  | Siroheme |
|  | Vitamin B12s |
|  | Porphobilinogen |
|  | Aquacobalamin |
|  | Hydroxymethylbilane |
|  | Uroporphyrinogen III |
|  | Protoporphyrinogen IX |
|  | Hemoglobin |
|  | Chlorophyllide |
|  | Protoporphyrin |
|  | Precorrin 2 |
|  | Uroporphyrin III |
|  | 4,5-Dioxopentanoate |
|  | Cyanocobalamin |
|  | Protochlorophyllide |
|  | L-Glutamyl-tRNA |
|  | Transferrin[Fe(II)]2 |
|  | Dimethylbenzimidazole |
|  | Transferrin[Fe(III)]2 |
|  | (R)-1-Aminopropan-2-ol |
|  | Coproporphyrinogen III |
|  | Magnesium protoporphyrin |
|  | (S)-4-Amino-5-oxopentanoate |
|  | D-1-Aminopropan-2-ol O-phosphate |
|  | Magnesium protoporphyrin monomethyl ester |
|  | N1-(5-Phospho-alpha-D-ribosyl)-5,6-dimethylbenzimidazole |
|  | Chlorophyll a |
|  | Chlorophyll b |
|  | Uroporphyrinogen I |
|  | Uroporphyrin I |
|  | Coproporphyrinogen I |
|  | Coproporphyrin I |
|  | Coproporphyrin III |
|  | Precorrin 3A |
|  | Cobyrinate |
|  | Cobinamide |
|  | alpha-Ribazole |
|  | Coenzyme F430 |
|  | Sirohydrochlorin |
|  | Apoferritin |
|  | Apotransferrin |
|  | Oxyhemoglobin |
|  | Myoglobin |
|  | Cytochrome a |
|  | Catalase |
|  | Peroxidase |
|  | (3Z)-Phycocyanobilin |
|  | Bilirubin beta-diglucuronide |
|  | L-Urobilinogen |
|  | I-Urobilinogen |
|  | D-Urobilinogen |
|  | L-Urobilin |
|  | Urobilin |
|  | D-Urobilin |
|  | Pheophytin a |
|  | Bacteriopheophytin |
|  | (3Z)-Phycoerythrobilin |
|  | (3Z)-Phytochromobilin |
|  | Precorrin 6Y |
|  | Precorrin 6X |
|  | Hydrogenobyrinate |
|  | Precorrin 3B |
|  | Precorrin 4 |
|  | Precorrin 8X |
|  | Precorrin 5 |
|  | Hydrogenobyrinate diamide |
|  | Cob(II)yrinate a,c diamide |
|  | Cob(I)yrinate a,c diamide |
|  | Adenosyl cobyrinate a,c diamide |
|  | Adenosyl cobyrinate hexaamide |
|  | Adenosyl cobinamide |
|  | Adenosyl cobinamide phosphate |
|  | Adenosine-GDP-cobinamide |
|  | Bacteriochlorophyll a |
|  | Bacteriochlorophyll b |
|  | Cobalt-sirohydrochlorin |
|  | Cobalt-precorrin 3 |
|  | Cobalt-precorrin 4 |
|  | Cobalt-precorrin 6 |
|  | Cobalt-dihydro-precorrin 6 |
|  | Cobalt-precorrin 8 |
|  | 15,16-Dihydrobiliverdin |
|  | 13(1)-Hydroxy-magnesium-protoporphyrin IX 13-monomethyl ester |
|  | 13(1)-Oxo-magnesium-protoporphyrin IX 13-monomethyl ester |
|  | Divinylprotochlorophyllide |
|  | Divinyl chlorophyllide a |
|  | Divinylchlorophyll a |
|  | Zn-Bacteriochlorophyll a |
|  | L-Threonine phosphate |
|  | Fe3+ |
|  | Heme A |
|  | Heme O |
|  | Cobalt-precorrin 5A |
|  | Cobalt-precorrin 5B |
|  | Cobalt-precorrin 7 |
|  | 7(1)-Hydroxychlorophyllide a |
|  | Chlorophyllide b |
|  | Cobalt-factor III |
|  | Pheophorbide a |
|  | Red chlorophyll catabolite |
|  | Pyropheophorbide a |
|  | Primary fluorescent chlorophyll catabolite |
|  | 7(1)-Hydroxychlorophyll a |
|  | 3-Vinylbacteriochlorophyllide a |
|  | 3-Hydroxyethylbacteriochlorophyllide a |
|  | 3-Hydroxyethylchlorophyllide a |
|  | Bacteriochlorophyllide a |
|  | Bacteriochlorophyllide b |
|  | 8-Ethyl-12-methyl-3-vinylbacteriochlorophyllide d |
|  | Bacteriochlorophyllide d |
|  | Bacteriochlorophyllide c |
|  | Bacteriochlorophyll d |
|  | Bacteriochlorophyll c |
|  | 5-Oxo-delta-bilirubin |
|  | Geranylgeranyl bacteriochlorophyllide a |
|  | Fe-coproporphyrin III |
|  | 8,12-Diethyl-3-vinylbacteriochlorophyllide d |
|  | 12-Ethyl-8-propyl-3-vinylbacteriochlorophyllide d |
|  | 12-Ethyl-8-isobutyl-3-vinylbacteriochlorophyllide d |
|  | 8-Ethyl-12-methylbacteriochlorophyllide d |
|  | 8,12-Diethylbacteriochlorophyllide d |
|  | 12-Ethyl-8-propylbacteriochlorophyllide d |
|  | 12-Ethyl-8-isobutylbacteriochlorophyllide d |
|  | Geranylgeranyl bacteriochlorophyllide b |
|  | Nickel-sirohydrochlorin |
|  | Nickel-sirohydrochlorin a,c-diamide |
|  | 15,17(3)-Seco-F430-17(3)-acid |
|  | Bacteriochlorophyllide e |
|  | Biliverdin-IX-delta |
|  | Biliverdin-IX-beta |
|  | 12,18-Didecarboxysiroheme |
|  |  |
| Gastric acid secretion | CO2 |
|  | H+ |
|  | HCO3- |
|  | Histamine |
|  | Carbonic acid |
|  |  |
| **Carbohydrate digestion and absorption** | CO2 |
|  | Acetic acid |
|  | Sucrose |
|  | D-Galactose |
|  | Maltose |
|  | Lactose |
|  | Butanoic acid |
|  | Hydrogen |
|  | Xylan |
|  | Pectin |
|  | Cellulose |
|  | Maltotriose |
|  | Limit dextrin |
|  | Gum arabic |
|  | Saccharin sodium anhydrous |
|  | Sucralose |
|  |  |
| **Drug metabolism - other enzymes** | 6-Mercaptopurine |
|  | 5-Fluorodeoxyuridine monophosphate |
|  | 6-Thioinosine-5'-monophosphate |
|  | Hydrazine |
|  | Fluoroacetate |
|  | Azathioprine |
|  | Isoniazid |
|  | Isonicotinic acid |
|  | Acetylhydrazine |
|  | N-Acetylisoniazid |
|  | Thioguanine |
|  | Fluorouracil |
|  | SN38 glucuronide |
|  | 5-Fluorodeoxyuridine |
|  | Capecitabine |
|  | Tegafur |
|  | 5'-Deoxy-5-fluorouridine |
|  | Carmofur |
|  | APC |
|  | NPC |
|  | 6-Thiourate |
|  | 6-Methylmercaptopurine |
|  | 6-Methylthiopurine 5'-monophosphate ribonucleotide |
|  | 6-Mercaptopurine ribonucleoside 5'-diphosphate |
|  | 6-Mercaptopurine ribonucleoside triphosphate |
|  | 6-Thioxanthine 5'-monophosphate |
|  | 6-Thioguanosine monophosphate |
|  | 6-Methylthioguanosine monophosphate |
|  | 1-Methyl-4-nitroimidazole |
|  | Diacetylhydrazine |
|  | Isonicotinuric acid |
|  | Isoniazid pyruvate |
|  | Isoniazid alpha-ketoglutaric acid |
|  | 5,6-Dihydro-5-fluorouracil |
|  | alpha-Fluoro-beta-ureidopropionic acid |
|  | alpha-Fluoro-beta-alanine |
|  | 5-Fluorouridine |
|  | 5-Fluorouridine monophosphate |
|  | 5'-Deoxy-5-fluorocytidine |
|  | Irinotecan |
|  | 5-Fluorouridine diphosphate |
|  | 5-Fluorouridine triphosphate |
|  | 5-Fluorodeoxyuridine diphosphate |
|  | 5-Fluorodeoxyuridine triphosphate |
|  | Isonicotinoyl radical |
|  | Hepatotoxins |
|  | Isonicotinoyl-NAD adduct |
|  | Isonicotinoyl-NADP adduct |
|  |  |
| **Cocaine addiction** | H2O2 |
|  | L-Tyrosine |
|  | 3,4-Dihydroxy-L-phenylalanine |
|  | 3,4-Dihydroxyphenylacetate |
|  | Dopamine |
|  |  |
| **Mineral absorption** | Phosphate |
|  | Heme |
|  | Glycine |
|  | Zn2+ |
|  | L-Glutamine |
|  | Cu+ |
|  | L-Tryptophan |
|  | L-Phenylalanine |
|  | H+ |
|  | D-Galactose |
|  | L-Proline |
|  | L-Asparagine |
|  | L-Valine |
|  | L-Threonine |
|  | HCO3- |
|  | Calcitriol |
|  | Fe3+ |
|  |  |
| Steroid biosynthesis | Cholesterol |
|  | trans,trans-Farnesyl diphosphate |
|  | Squalene |
|  | (S)-2,3-Epoxysqualene |
|  | Provitamin D3 |
|  | Lathosterol |
|  | Calcidiol |
|  | Calcitriol |
|  | Ergosterol |
|  | Lanosterol |
|  | Sitosterol |
|  | Campesterol |
|  | Desmosterol |
|  | Cycloartenol |
|  | Obtusifoliol |
|  | Cycloeucalenol |
|  | Presqualene diphosphate |
|  | Cholesterol ester |
|  | 5alpha-Cholest-8-en-3beta-ol |
|  | Fecosterol |
|  | 4alpha-Methylzymosterol |
|  | 7-Dehydrodesmosterol |
|  | 14-Demethyllanosterol |
|  | 24,25-Dihydrolanosterol |
|  | Zymosterol |
|  | 5alpha-Cholesta-7,24-dien-3beta-ol |
|  | Ergosta-5,7,22,24(28)-tetraen-3beta-ol |
|  | Vitamin D2 |
|  | Stigmasterol |
|  | Vitamin D3 |
|  | Secalciferol |
|  | Brassicasterol |
|  | Isofucosterol |
|  | 24-Methylidenecycloartanol |
|  | 4,4-Dimethyl-5alpha-cholesta-8,14,24-trien-3beta-ol |
|  | 4alpha-Methyl-5alpha-ergosta-8,14,24(28)-trien-3beta-ol |
|  | 24-Methylenelophenol; 24-Methylidenelophenol |
|  | 24-Ethylidenelophenol; alpha1-Sitosterol |
|  | 4alpha-Methylfecosterol |
|  | Episterol |
|  | 5,7,24(28)-Ergostatrienol |
|  | 5-Dehydroepisterol |
|  | 24-Methylenecholesterol |
|  | Delta7-Avenasterol |
|  | 5-Dehydroavenasterol |
|  | 4alpha-Methylzymosterol-4-carboxylate |
|  | 3-Keto-4-methylzymosterol |
|  | 4,4-Dimethyl-5alpha-cholesta-8-en-3beta-ol |
|  | Calcitetrol |
|  | 24-epi-Campesterol |
|  | 4alpha-Carboxy-5alpha-cholesta-8,24-dien-3beta-ol |
|  | 3beta-Hydroxy-4beta,14alpha-dimethyl-9beta,19-cyclo-5alpha-ergost-24(24(1))-en-4alpha-carboxylate |
|  | 3beta-Hydroxyergosta-7,24(24(1))-dien-4alpha-carboxylate |
|  | 4alpha-Carboxy-stigmasta-7,24(24(1))-dien-3beta-ol |
|  | Cycloeucalenone |
|  | Episterone |
|  | Avenastenone |
|  | Zymosterone |
|  |  |
| **Arginine biosynthesis** | CO2 |
|  | L-Arginine |
|  | L-Glutamine |
|  | L-Ornithine |
|  | Carbamoyl phosphate |
|  | L-Citrulline |
|  | N-Acetylornithine |
|  | N-Acetyl-L-glutamate |
|  | Urea-1-carboxylate |
|  | N-Acetyl-L-glutamate 5-semialdehyde |
|  | N-(L-Arginino)succinate |
|  | N-Acetyl-L-glutamate 5-phosphate |
|  | N-Acetyl-L-citrulline |
|  | LysW-L-glutamate |
|  | LysW-L-glutamyl 5-phosphate |
|  | LysW-L-glutamate 5-semialdehyde |
|  | LysW-L-ornithine |
|  |  |
| **Proximal tubule bicarbonate reclamation** | H2O |
|  | CO2 |
|  | L-Glutamine |
|  | H+ |
|  | (S)-Malate |
|  | HCO3- |
|  | Carbonic acid |
|  | NH4OH |
|  |  |
| Steroid hormone biosynthesis | Cholesterol |
|  | Androstenedione |
|  | Progesterone |
|  | Estrone |
|  | Androsterone |
|  | Testosterone |
|  | 5alpha-Androstane-3,17-dione |
|  | Cortisol |
|  | Cortisone |
|  | Estradiol-17beta |
|  | 18-Hydroxycorticosterone |
|  | 17alpha-Hydroxyprogesterone |
|  | Dehydroepiandrosterone |
|  | Aldosterone |
|  | Pregnenolone |
|  | Corticosterone |
|  | 4-Methylpentanal |
|  | Estradiol-17alpha |
|  | Estrone 3-sulfate |
|  | 11-Deoxycorticosterone |
|  | 5alpha-Pregnane-3,20-dione |
|  | 11alpha-Hydroxyprogesterone |
|  | 5beta-Androstane-3,17-dione |
|  | Androstan-3alpha,17beta-diol |
|  | Dihydrotestosterone |
|  | 6beta-Hydroxy-17beta-estradiol |
|  | 20alpha-Hydroxy-4-pregnen-3-one |
|  | Androstenediol |
|  | Etiocholanolone |
|  | 17alpha,20alpha-Dihydroxypregn-4-en-3-one |
|  | Dehydroepiandrosterone sulfate |
|  | Testololactone |
|  | 17alpha-Hydroxypregnenolone |
|  | 16alpha-Hydroxydehydroepiandrosterone |
|  | 16alpha-Hydroxyandrost-4-ene-3,17-dione |
|  | Estriol |
|  | 11beta-Hydroxyandrost-4-ene-3,17-dione |
|  | androst-4-ene-3,17-dione; Androst-4-ene-3,17-dione-11beta-ol; 4-Androsten-11beta-ol-3, |
|  | Adrenosterone |
|  | 19-Hydroxyandrost-4-ene-3,17-dione |
|  | 7alpha-Hydroxytestosterone |
|  | 5beta-Dihydrotestosterone |
|  | 19-Hydroxytestosterone |
|  | 19-Oxotestosterone |
|  | 7alpha-Hydroxyandrost-4-ene-3,17-dione |
|  | 19-Oxoandrost-4-ene-3,17-dione |
|  | 2-Hydroxyestrone |
|  | 2-Methoxyestrone |
|  | 16alpha-Hydroxyestrone |
|  | 2-Hydroxyestradiol |
|  | 2-Methoxy-17beta-estradiol |
|  | 17alpha,21-Dihydroxy-5beta-pregnane-3,11,20-trione |
|  | Tetrahydrocortisone |
|  | 11beta,17alpha,21-Trihydroxy-5beta-pregnane-3,20-dione |
|  | Urocortisol |
|  | 11beta,21-Dihydroxy-3,20-oxo-5beta-pregnan-18-al |
|  | 3alpha,11beta,21-Trihydroxy-20-oxo-5beta-pregnan-18-al |
|  | 11beta,21-Dihydroxy-5beta-pregnane-3,20-dione |
|  | Tetrahydrocorticosterone |
|  | 21-Hydroxy-5beta-pregnane-3,11,20-trione |
|  | 3alpha,21-Dihydroxy-5beta-pregnane-11,20-dione |
|  | 5beta-Pregnane-3,20-dione |
|  | Pregnanolone |
|  | Cortolone |
|  | Cortol |
|  | 3alpha,20alpha,21-Trihydroxy-5beta-pregnan-11-one |
|  | Pregnanediol |
|  | 21-Hydroxypregnenolone |
|  | 17alpha,21-Dihydroxypregnenolone |
|  | 11-Deoxycortisol |
|  | 11beta,17alpha,21-Trihydroxypregnenolone |
|  | 11-Dehydrocorticosterone |
|  | 21-Deoxycortisol |
|  | 11beta-Hydroxyprogesterone |
|  | 17alpha,20alpha-Dihydroxycholesterol |
|  | 20alpha-Hydroxycholesterol |
|  | 20alpha,22beta-Dihydroxycholesterol |
|  | 22(R)-Hydroxycholesterol |
|  | Estradiol-17beta 3-glucuronide |
|  | 16-Glucuronide-estriol |
|  | Estradiol-17beta 3-sulfate |
|  | 2-Methoxyestrone 3-sulfate |
|  | 2-Methoxyestradiol-17beta 3-sulfate |
|  | 2-Methoxy-estradiol-17beta 3-glucuronide |
|  | 2-Methoxyestrone 3-glucuronide |
|  | Estrone glucuronide |
|  | Testosterone glucuronide |
|  | Androsterone glucuronide |
|  | Etiocholan-3alpha-ol-17-one 3-glucuronide |
|  | Allopregnanolone |
|  | Allotetrahydrodeoxycorticosterone |
|  | 7alpha-Hydroxypregnenolone |
|  | Aldosterone hemiacetal |
|  | 5alpha-Dihydrodeoxycorticosterone |
|  | 5alpha-Pregnan-20alpha-ol-3-one |
|  | 5alpha-Pregnane-3alpha,20alpha-diol |
|  | Cholesterol sulfate |
|  | 3beta-Hydroxypregn-5-en-20-one sulfate |
|  | 7alpha-Hydroxydehydroepiandrosterone |
|  | 11beta,17beta-Dihydroxy-4-androsten-3-one |
|  |  |
| Retinol metabolism | Retinol |
|  | Retinoate |
|  | Rhodopsin |
|  | 11-cis-Retinol |
|  | Retinyl ester |
|  | beta-Carotene |
|  | Retinyl palmitate |
|  | 11-cis-Retinyl palmitate |
|  | Bathorhodopsin |
|  | Lumirhodopsin |
|  | Metarhodopsin |
|  | Iodopsin |
|  | all-trans-Retinoyl-beta-glucuronide |
|  | all-trans-13,14-Dihydroretinol |
|  | all-trans-4-Hydroxyretinoic acid |
|  | all-trans-4-Oxoretinoic acid |
|  | all-trans-18-Hydroxyretinoic acid |
|  | all-trans-5,6-Epoxyretinoic acid |
|  | 9-cis-Retinal |
|  | 9-cis-Retinol |
|  | 4-Oxoretinol |
|  | all-trans-3,4-Didehydroretinol |
|  |  |
| Citrate cycle (TCA cycle) | Succinic acid |
|  | Succinyl-CoA |
|  | (S)-Malate |
|  | Citrate |
|  | Isocitrate |
|  | cis-Aconitate |
|  | Oxalosuccinate |
|  | 3-Carboxy-1-hydroxypropyl-ThPP |
|  | [Dihydrolipoyllysine-residue succinyltransferase] S-succinyldihydrolipoyllysine |
|  |  |
| Metabolism of xenobiotics by cytochrome P450 - Homo sapiens | Nicotine |
|  | Naphthalene |
|  | 1,4-Naphthoquinone |
|  | Naphthalene-1,2-diol |
|  | 1,2-Dihydronaphthalene-1,2-diol |
|  | Chloroacetic acid |
|  | Trichloroethene |
|  | Aflatoxin B1 |
|  | Chloral hydrate |
|  | Trichloroethanol |
|  | Benzpyrene |
|  | Bromobenzene |
|  | Ethylene dibromide |
|  | Trichloroethylene epoxide |
|  | Dichloroacetate |
|  | Trichloroacetate |
|  | Aflatoxin B1exo-8,9-epoxide-GSH |
|  | 2-Naphthol |
|  | 1-Naphthol |
|  | 1,1-Dichloroethylene |
|  | 1-Nitronaphthalene |
|  | 4-Bromophenol |
|  | 9-Hydroxybenzo[a]pyrene |
|  | 1,2-Naphthoquinone |
|  | 1,2-Dihydroxy-3,4-epoxy-1,2,3,4-tetrahydronaphthalene |
|  | 1,4-Dihydroxynaphthalene |
|  | (1R,2S)-Naphthalene 1,2-oxide |
|  | (1S,2R)-Naphthalene 1,2-oxide |
|  | 1-Nitrosonaphthalene |
|  | N-Hydroxy-1-aminonaphthalene |
|  | 1-Naphthylamine |
|  | (1R)-Hydroxy-(2R)-glutathionyl-1,2-dihydronaphthalene |
|  | (1S)-Hydroxy-(2S)-glutathionyl-1,2-dihydronaphthalene |
|  | (1R)-Glutathionyl-(2R)-hydroxy-1,2-dihydronaphthalene |
|  | (1R)-Hydroxy-(2R)-N-acetyl-L-cysteinyl-1,2-dihydronaphthalene |
|  | (1R)-N-Acetyl-L-cysteinyl-(2R)-hydroxy-1,2-dihydronaphthalene |
|  | (1S)-Hydroxy-(2S)-N-acetyl-L-cysteinyl-1,2-dihydronaphthalene |
|  | (1R,2R)-3-[(1,2-Dihydro-2-hydroxy-1-naphthalenyl)thio]-2-oxopropanoic acid |
|  | 1-Nitronaphthalene-5,6-oxide |
|  | 1-Nitro-5,6-dihydroxy-dihydronaphthalene |
|  | 1-Nitronaphthalene-7,8-oxide |
|  | 1-Nitro-7-hydroxy-8-glutathionyl-7,8-dihydronaphthalene |
|  | 1-Nitro-7-glutathionyl-8-hydroxy-7,8-dihydronaphthalene |
|  | 1-Nitro-5-hydroxy-6-glutathionyl-5,6-dihydronaphthalene |
|  | 1-Nitro-5-glutathionyl-6-hydroxy-5,6-dihydronaphthalene |
|  | Bromobenzene-3,4-oxide |
|  | Bromobenzene-2,3-oxide |
|  | 2-Bromophenol |
|  | Bromobenzene-2,3-dihydrodiol |
|  | 4-Bromocatechol |
|  | Bromobenzene-3,4-dihydrodiol |
|  | 4-Bromophenol-2,3-epoxide |
|  | 4-Bromo-3,5-cyclohexadiene-1,2-dione |
|  | 3,4-Dihydro-3-hydroxy-4-S-glutathionyl bromobenzene |
|  | 2,3-Dihydro-2-S-glutathionyl-3-hydroxy bromobenzene |
|  | Benzo[a]pyrene-9,10-oxide; Benzo[a]pyrene-9,10-epoxide |
|  | Benzo[a]pyrene-7,8-oxide; Benzo[a]pyrene-7,8-epoxide |
|  | Benzo[a]pyrene-4,5-oxide; Benzo[a]pyrene-4,5-epoxide |
|  | Benzo[a]pyrene-7,8-diol; Benzo[a]pyrene-7,8-dihydrodiol |
|  | Benzo[a]pyrene-7,8-dihydrodiol-9,10-oxide; 7,8-Dihydro-7,8-dihydroxybenzo[a]pyrene 9,10-oxide |
|  | 9-Hydroxybenzo[a]pyrene-4,5-oxide; 9-Hydroxybenzo[a]pyrene-4,5-epoxide |
|  | 4,5-Dihydro-4-hydroxy-5-S-glutathionyl-benzo[a]pyrene |
|  | 7,8-Dihydro-7-hydroxy-8-S-glutathionyl-benzo[a]pyrene |
|  | 1,1-Dichloroethylene epoxide; 2,2-Dichlorooxirane |
|  | 2,2-Dichloroacetaldehyde |
|  | Chloroacetyl chloride |
|  | 2,2-Dichloro-1,1-ethanediol |
|  | S-(2,2-Dichloro-1-hydroxy)ethyl glutathione |
|  | 2-S-Glutathionyl acetate; S-(Carboxymethyl)glutathione |
|  | 2-(S-Glutathionyl)acetyl glutathione |
|  | S-(2-Chloroacetyl)glutathione |
|  | 2-(S-Glutathionyl)acetyl chloride |
|  | Chloral |
|  | Dichloroacetyl chloride |
|  | S-(1,2-Dichlorovinyl)glutathione |
|  | Trichloroethanol glucuronide |
|  | S-(Formylmethyl)glutathione |
|  | 2-Bromoacetaldehyde |
|  | Thiodiacetic acid |
|  | Thiodiacetic acid sulfoxide |
|  | Glutathione episulfonium ion |
|  | S-(2-Hydroxyethyl)glutathione |
|  | S-(2-Hydroxyethyl)-N-acetyl-L-cysteine |
|  | S-[2-(N7-Guanyl)ethyl]-N-acetyl-L-cysteine |
|  | 4-(N-Nitrosomethylamino)-1-(3-pyridyl)-1-butanone |
|  | Aflatoxin M1 |
|  | 7,12-Dimethylbenz[a]anthracene |
|  | 1a,11b-Dihydro-4,9-dimethylbenz[a]anthra[3,4-b]oxirene |
|  | trans-3,4-Dihydro-3,4-dihydroxy-7,12-dimethylbenz[a]anthracene |
|  | (1aalpha,2beta,3alpha,11calpha)-1a,2,3,11c-Tetrahydro-6,11-dimethylbenzo[6,7]phenanthro[3,4-b]oxiren |
|  | 7-Hydroxymethyl-12-methylbenz[a]anthracene |
|  | 7-Hydroxymethyl-12-methylbenz[a]anthracene sulfate |
|  | 4-[(Hydroxymethyl)nitrosoamino]-1-(3-pyridinyl)-1-butanone |
|  | 4-(Nitrosoamino)-1-(3-pyridinyl)-1-butanone |
|  | 4-Hydroxy-1-(3-pyridinyl)-1-butanone |
|  | 4-Hydroxy-4-(methylnitrosoamino)-1-(3-pyridinyl)-1-butanone |
|  | N-Nitrosomethanamine |
|  | 4-(Methylnitrosamino)-1-(3-pyridyl)-1-butanol |
|  | 1-(Methylnitrosoamino)-4-(3-pyridinyl)-1,4-butanediol |
|  | 5-(3-Pyridyl)-2-hydroxytetrahydrofuran |
|  | gamma-Hydroxy-3-pyridinebutanoate |
|  | alpha-[3-[(Hydroxymethyl)nitrosoamino]propyl]-3-pyridinemethanol |
|  | alpha-[3-(Nitrosoamino)propyl]-3-pyridinemethanol |
|  | 1-(3-Pyridinyl)-1,4-butanediol |
|  | Aflatoxin Q1 |
|  | Aflatoxin B1-exo-8,9-epoxide |
|  | Aflatoxin B1 diol |
|  | Aflatoxin B1 dialdehyde |
|  | 6-[2,3-Dihydroxy-1-(hydroxymethyl)propyl]-1,2-dihydro-7-hydroxy-9-methoxy-cyclopenta[c][1]benzopyran |
|  | 1,2,3,4-Tetrahydro-alpha,7-dihydroxy-beta-(hydroxymethyl)-9-methoxy-3,4-dioxocyclopenta[c][1]benzopy |
|  | alpha-(1,2-Dihydroxyethyl)-1,2,3,4-tetrahydro-7-hydroxy-9-methoxy-3,4-dioxocyclopenta[c][1]benzopyra |
|  | Aflatoxin-M1-8,9-epoxide |
|  | Aflatoxin B1-endo-8,9-epoxide |
|  | 4-(Methylnitrosamino)-1-(1-oxido-3-pyridinyl)-1-butanone |
|  | 4-(Methylnitrosamino)-1-(3-pyridyl-N-oxide)-1-butanol |
|  | 7,12-Dimethylbenz[a]anthracene 5,6-oxide; DMBA-5,6-epoxide |
|  | 4-(Methylnitrosamino)-1-(3-pyridyl)-1-butanol glucuronide |
|  | NNAL-N-glucuronide |
|  | trans-5,6-Dihydro-5,6-dihydroxy-7,12-dimethylbenz[a]anthracene |
|  |  |
| Prion diseases | H2O2 |
|  | Corticosterone |
|  |  |
| Aldosterone-regulated sodium reabsorption | Cortisol |
|  | Cortisone |
|  | Aldosterone |
|  |  |
| GnRH signaling pathway | Arachidonate |
| Nicotine addiction | Nicotine |
|  |  |
| ABC transporters | Heme |
|  | Manganese |
|  | Glycine |
|  | Zn2+ |
|  | L-Lysine |
|  | L-Arginine |
|  | L-Glutamine |
|  | Cu2+ |
|  | L-Ornithine |
|  | L-Phenylalanine |
|  | Nitrite |
|  | Sucrose |
|  | Oligopeptide |
|  | Dipeptide |
|  | Biotin |
|  | Putrescine |
|  | L-Histidine |
|  | myo-Inositol |
|  | N-Acetyl-D-glucosamine |
|  | L-Proline |
|  | L-Amino acid |
|  | Co2+ |
|  | L-Valine |
|  | Cellobiose |
|  | L-Threonine |
|  | Maltose |
|  | Adenosine |
|  | Lactose |
|  | Nitrate |
|  | Taurine |
|  | Riboflavin |
|  | L-Arabinose |
|  | HCO3- |
|  | Nickel |
|  | Inosine |
|  | Uridine |
|  | Spermidine |
|  | Thiosulfate |
|  | Deoxyguanosine |
|  | D-Galacturonate |
|  | Thiamine |
|  | Xylitol |
|  | Guanosine |
|  | 5-Aminolevulinate |
|  | Pectate |
|  | Cytidine |
|  | Carnitine |
|  | Raffinose |
|  | Erythritol |
|  | Deoxyuridine |
|  | Deoxyadenosine |
|  | Betaine |
|  | Tungsten |
|  | D-Methionine |
|  | Phospholipid |
|  | Deoxycytidine |
|  | Choline sulfate |
|  | Cyclomaltodextrin |
|  | alpha,alpha-Trehalose |
|  | Orthophosphoric monoester |
|  | Hydroxyproline |
|  | Inositol 1-phosphate |
|  | 4-Trimethylammoniobutanoate |
|  | 4-Amino-5-hydroxymethyl-2-methylpyrimidine |
|  | Cyanate |
|  | Phthalate |
|  | Xylobiose |
|  | Bacitracin |
|  | Chitobiose |
|  | Nopaline |
|  | D-Rhamnose |
|  | Xanthosine |
|  | Lipoprotein |
|  | Maltotriose |
|  | Maltodextrin |
|  | Oleandomycin |
|  | Isomaltotriose |
|  | Digalacturonate |
|  | 2-Aminoethylphosphonate |
|  | Galactose oligosaccharide |
|  | Methyl beta-D-galactoside |
|  | (E)-4-(Trimethylammonio)but-2-enoate |
|  | D-Octopine; N2-(D-1-Carboxyethyl)-L-arginine |
|  | Ciprofloxacin |
|  | Melibiose |
|  | Deoxyinosine |
|  | Vitamin B12 |
|  | Fe(III)hydroxamate |
|  | Fe(III)dicitrate |
|  | Fe-enterobactin |
|  | Molybdate |
|  | Norfloxacin |
|  | Capsular polysaccharide |
|  | Lipo-oligosaccharide |
|  | Teichoic acid |
|  | Hemine |
|  | Sparfloxacin |
|  | Moxifloxacin |
|  | Polymyxin B |
|  | Colistin sulfate |
|  | Fe3+ |
|  | Alkanesulfonate |
|  | AI-2 |
|  | Mannopine |
|  | Nickel(2+) |
|  | N-Formyl-4-amino-5-aminomethyl-2-methylpyrimidine |
|  | alpha-1,5-L-Arabinobiose |
|  | alpha-1,5-L-Arabinotriose |
|  | alpha-1,5-L-Arabinotetraose |
|  | Aldotetraouronic acid |
|  | D-Galactofuranose |
|  | S-Methyl-L-cysteine |
|  |  |
| Endocrine and other factor-regulated calcium reabsorption | Estradiol-17beta |
|  | Calcitriol |
| Taste transduction | H+ |
|  | Sucrose |
|  | Inosinic acid |
|  | D-Alanine |
|  | (S)-Malate |
|  | Citrate |
|  | Maltose |
|  | L-Noradrenaline |
|  | D-Serine |
|  | Serotonin |
|  | HCl |
|  | D-Phenylalanine |
|  | Quinine |
|  | Aspartame |
|  | Saccharin |
|  | Saccharin sodium anhydrous |
|  | Cyclamate |
|  |  |
| Progesterone-mediated oocyte maturation | Progesterone |
|  | 1-Phosphatidyl-1D-myo-inositol 3,4-bisphosphate |
|  |  |
| Glucagon signaling pathway | (S)-Malate |
|  | Citrate |
|  | Glycogen |
|  | Isocitrate |
|  | D-Fructose 1,6-bisphosphate |
|  |  |

**Table S17.** Exclusive pathways and their respective metabolites related to symptomatic HD.

| **Pathway** | **Metabolite** |
| --- | --- |
| Nicotinate and nicotinamide | Glycerone phosphate |
|  | Nicotinamide |
|  | Succinate semialdehyde |
|  | Nicotinate |
|  | Nicotinamide D-ribonucleotide |
|  | Deamino-NAD+ |
|  | 2,3-Dimethylmaleate |
|  | N-Methylnicotinate |
|  | 6-Hydroxynicotinate |
|  | (S)-6-Hydroxynicotine |
|  | 2,5-Dihydroxypyridine |
|  | Nicotinate D-ribonucleotide |
|  | 6-Hydroxypseudooxynicotine |
|  | Maleic acid |
|  | Maleamate |
|  | Methylitaconate |
|  | 1-Methylnicotinamide |
|  | 2-Methyleneglutarate |
|  | (R)-6-Hydroxynicotine |
|  | 2,6-Dihydroxypyridine |
|  | Nicotinamide-beta-riboside |
|  | 2,3,6-Trihydroxypyridine |
|  | (2R,3S)-2,3-Dimethylmalate |
|  | Quinolinate |
|  | 6-Oxo-1,4,5,6-tetrahydronicotinate |
|  | Nicotinurate |
|  | Iminoaspartate |
|  | Nicotinate D-ribonucleoside |
|  | N1-Methyl-2-pyridone-5-carboxamide |
|  | N1-Methyl-4-pyridone-5-carboxamide |
|  | 1-Methylpyrrolinium |
|  | 2,6-Dihydroxynicotinate |
|  | 2,6-Dihydroxypseudooxynicotine |
|  | 4-Methylaminobutyrate |
|  | (R,S)-Nicotine |
|  | 2,6-Dihydroxy-N-methylmyosmine |
|  | Nicotine blue |
|  | 2-Formylglutarate |
|  | (S)-2-(Hydroxymethyl)glutarate |
|  | N-Formylmaleamic acid |
|  | 6-Hydroxy-3-succinoylpyridine |
|  | Pseudooxynicotine |
|  | 3,3'-Bipyridine-2,2',5,5',6,6'-hexol |
|  | 5-(N-Methyl-4,5-dihydro-1H-pyrrol-2-yl)pyridin-2-ol |
|  | N-Methylmyosmine |
|  |  |
| Fructose and mannose metabolism | Glycerone phosphate |
|  | D-Glyceraldehyde 3-phosphate |
|  | L-Sorbose |
|  | D-Mannose 6-phosphate |
|  | GDP-L-fucose |
|  | (S)-Lactaldehyde |
|  | Mannan |
|  | L-Rhamnose |
|  | D-Glyceraldehyde |
|  | D-Mannose 1-phosphate |
|  | D-Mannitol 1-phosphate |
|  | L-Rhamnulose |
|  | GDP-D-mannuronate |
|  | 6-Deoxy-L-galactose |
|  | D-Fructose 1-phosphate |
|  | Sorbitol 6-phosphate |
|  | L-Fuculose 1-phosphate |
|  | L-Rhamnulose 1-phosphate |
|  | GDP-4-dehydro-6-deoxy-D-mannose |
|  | L-Fuconate |
|  | L-Fuculose |
|  | Alginic acid |
|  | L-Rhamnonate |
|  | L-Rhamnofuranose |
|  | 1,4-beta-D-Mannan |
|  | Sorbose 1-phosphate |
|  | D-Allose 6-phosphate |
|  | GDP-6-deoxy-D-talose |
|  | L-Fucose 1-phosphate |
|  | L-Rhamnono-1,4-lactone |
|  | GDP-6-deoxy-D-mannose |
|  | beta-D-Fructose 2-phosphate |
|  | 2-Dehydro-3-deoxy-L-fuconate |
|  | 2-Dehydro-3-deoxy-L-rhamnonate |
|  | Mannuronan |
|  | beta-D-Fructose 6-phosphate |
|  | beta-D-Fructose 1,6-bisphosphate |
|  | Oligouronide with 4-deoxy-alpha-L-erythro-hex-4-enopyranuronosyl group |
|  | ADP-mannose |
|  | 2-(alpha-D-Mannosyl)-3-phosphoglycerate |
|  | 2-O-(alpha-D-Mannosyl)-D-glycerate |
|  | L-Fucono-1,5-lactone |
|  | D-Allulose 6-phosphate |
|  | 2,4-Diketo-3-deoxy-L-fuconate |
|  |  |
| Fatty acid biosynthesis | Palmitoyl-CoA |
|  | Acyl-[acyl-carrier protein] |
|  | Acyl-carrier protein |
|  | Hexadecanoic acid |
|  | Malonate |
|  | Long-chain fatty acid |
|  | 3-Oxoacyl-[acyl-carrier protein] |
|  | trans-2,3-Dehydroacyl-[acyl-carrier protein] |
|  | (9Z)-Octadecenoic acid |
|  | Oleoyl-[acyl-carrier protein] |
|  | Malonyl-[acyl-carrier protein] |
|  | (3R)-3-Hydroxyacyl-[acyl-carrier protein] |
|  | Octadecanoic acid |
|  | Decanoic acid |
|  | Dodecanoic acid |
|  | Long-chain acyl-CoA |
|  | Acetyl-[acyl-carrier protein] |
|  | Octadecanoyl-[acyl-carrier protein] |
|  | cis-Dec-3-enoyl-[acp] |
|  | But-2-enoyl-[acyl-carrier protein] |
|  | (3R)-3-Hydroxybutanoyl-[acyl-carrier protein] |
|  | (3R)-3-Hydroxydecanoyl-[acyl-carrier protein] |
|  | (3R)-3-Hydroxyoctanoyl-[acyl-carrier protein] |
|  | (3R)-3-Hydroxypalmitoyl-[acyl-carrier protein |
|  | (3R)-3-Hydroxytetradecanoyl-[acyl-carrier protein] |
|  | Dodecanoyl-[acyl-carrier protein] |
|  | Acetoacetyl-[acp] |
|  | Butyryl-[acp] |
|  | 3-Oxohexanoyl-[acp] |
|  | (R)-3-Hydroxyhexanoyl-[acp] |
|  | trans-Hex-2-enoyl-[acp] |
|  | Hexanoyl-[acp] |
|  | 3-Oxooctanoyl-[acp] |
|  | trans-Oct-2-enoyl-[acp] |
|  | Octanoyl-[acp] |
|  | 3-Oxodecanoyl-[acp] |
|  | trans-Dec-2-enoyl-[acp] |
|  | Decanoyl-[acp] |
|  | 3-Oxododecanoyl-[acp] |
|  | trans-Dodec-2-enoyl-[acp] |
|  | (R)-3-Hydroxydodecanoyl-[acp] |
|  | 3-Oxotetradecanoyl-[acp] |
|  | trans-Tetradec-2-enoyl-[acp] |
|  | Tetradecanoyl-[acp]; |
|  | 3-Oxohexadecanoyl-[acp] |
|  | trans-Hexadec-2-enoyl-[acp] |
|  | Hexadecanoyl-[acp] |
|  | Octanoic acid |
|  | Tetradecanoic acid |
|  | (9Z)-Hexadecenoic acid |
|  | 3-Oxostearoyl-[acp] |
|  | (R)-3-Hydroxyoctadecanoyl-[acp] |
|  | (2E)-Octadecenoyl-[acp] |
|  | Hexadecenoyl-[acyl-carrier protein] |
|  | Long-chain acyl-[acyl-carrier protein] |
|  | n-7 Unsaturated acyl-[acyl-carrier protein] |
|  |  |
|  |  |
|  |  |
|  |  |
|  |  |
|  |  |
|  |  |
|  |  |
|  |  |
|  |  |
|  |  |
|  |  |
|  |  |
|  |  |
|  |  |
|  |  |
|  |  |
|  |  |
|  |  |
|  |  |
|  |  |
|  |  |
|  |  |
|  |  |
|  |  |
|  |  |
|  |  |
|  |  |
|  |  |
|  |  |
|  |  |
|  |  |
|  |  |
|  |  |
|  |  |
|  |  |
|  |  |
|  |  |
|  |  |
|  |  |
|  |  |
|  |  |
|  |  |
|  |  |
|  |  |
|  |  |
|  |  |
|  |  |
|  |  |
| Cysteine and methionine metabolism | S-Adenosyl-L-methionine |
|  | S-Adenosyl-L-homocysteine |
|  | Sulfite |
|  | L-Cysteine |
|  | 2-Oxobutanoate |
|  | L-Homocysteine |
|  | 5'-Methylthioadenosine |
|  | L-Homoserine |
|  | Hydrogen sulfide |
|  | Methanethiol |
|  | L-Aspartate 4-semialdehyde |
|  | L-Cysteate |
|  | 3-Sulfino-L-alanine |
|  | D-Cysteine |
|  | Mercaptopyruvate |
|  | O-Acetyl-L-serine |
|  | O-Phospho-L-serine |
|  | O-Acetyl-L-homoserine |
|  | O-Succinyl-L-homoserine |
|  | S-Adenosylmethioninamine |
|  | 4-Methylthio-2-oxobutanoic acid |
|  | 1-Aminocyclopropane-1-carboxylate |
|  | L-Homocystine |
|  | Thiocysteine |
|  | Dehydroalanine |
|  | L-Cystathionine |
|  | (S)-2-Aminobutanoate |
|  | L-Methionine S-oxide |
|  | 4-Phospho-L-aspartate |
|  | 5-Methylthio-D-ribose |
|  | N-Formylmethionine |
|  | 3-Phosphonooxypyruvate |
|  | S-Ribosyl-L-homocysteine |
|  | S-Methyl-5-thio-D-ribose 1-phosphate |
|  | S-Methyl-5-thio-D-ribulose 1-phosphate |
|  | Aminoacyl-L-methionine |
|  | S-Glutathionyl-L-cysteine |
|  | 3-Sulfinylpyruvate |
|  | 3-Sulfopyruvate |
|  | 3-Mercaptolactate |
|  | S-Sulfo-L-cysteine |
|  | Ethylene |
|  | 3-(Methylthio)propanoate |
|  | Sulfur dioxide |
|  | HSO3- |
|  | (S)-3-Sulfolactate |
|  | (2R)-3-Sulfolactate |
|  | 1,2-Dihydroxy-5-(methylthio)pent-1-en-3-one |
|  | 2,3-Diketo-5-methylthiopentyl-1-phosphate |
|  | 2-Hydroxy-3-keto-5-methylthiopentenyl-1-phosphate |
|  | N-Acyl-L-homoserine lactone |
|  | 5'-S-Methyl-5'-thioinosine gamma-L-Glutamyl-L-2-aminobutyrate |
|  | Ophthalmate |
|  |  |
| Biosynthesis of unsaturated fatty | Palmitoyl-CoA |
|  | Arachidonic acid |
|  | Hexadecanoic acid |
|  | Stearoyl-CoA |
|  | Oleoyl-CoA |
|  | (9Z)-Octadecenoic acid |
|  | Oleoyl-[acyl-carrier protein] |
|  | Octadecanoic acid |
|  | Linoleic acid |
|  | Eicosanoyl-CoA |
|  | Linoleoyl-CoA |
|  | Arachidonyl-CoA |
|  | gamma-Linolenoyl-CoA |
|  | Dihomo-gamma-linolenate |
|  | 8,11,14-Eicosatrienoyl-CoA |
|  | Octadecanoyl-[acyl-carrier protein] |
|  | Hexadecanoyl-[acp] |
|  | Eicosanoic acid |
|  | Octadecatrienoic acid |
|  | Octadecatrienoic acid |
|  | cosapentaenoic acid |
|  | Docosahexaenoic acid |
|  | Docosanoic acid |
|  | Erucic acid |
|  | Tetracosanoic acid |
|  | Tetracosenoic acid |
|  | Linoleoyl-[acyl-carrier protein] |
|  | alpha-Linolenoyl-ACP |
|  | Octadecatrienoyl-CoA |
|  | Icosatetraenoyl-CoA |
|  | Icosapentaenoyl-CoA |
|  | Docosapentaenoyl-CoA |
|  | Tetracosapentaenoyl-CoA |
|  | Tetracosahexaenoyl-CoA |
|  | Docosahexaenoyl-CoA |
|  | Docosatetraenoyl-CoA |
|  | Tetracosatetraenoyl-CoA |
|  | Tetracosapentaenoyl-CoA |
|  | Docosapentaenoyl-CoA |
|  | Icosatrienoyl-CoA |
|  | Icosadienoyl-CoA |
|  | Docosapentaenoic acid |
|  | Eicosatrienoic acid |
|  | Eicosadienoic acid |
|  | Eicosenoic acid |
|  | Adrenic acid |
|  | Docosanoyl-CoA |
|  | Tetracosanoyl-CoA |
|  | Eicosenoyl-CoA |
|  | Docosenoyl-CoA |
|  | Tetracosenoyl-CoA |
|  | Docosadienoic acid |
|  | Docosadienoyl-CoA |
|  | Ximenoyl-CoA |
|  | Palmitoleoyl-CoA |
|  | Cerotoyl-CoA |
|  | Montanoyl-CoA |
|  | Octadecadienoyl-CoA |
|  | Icosadienoyl-CoA; |
|  | Icosatrienoyl-CoA |
|  | Docosatrienoyl-CoA |
|  | Sapienoyl-CoA |
|  | cis-Vaccenoyl-CoA |
|  | Paullinoyl-CoA |
|  | Hexacosapentaenoyl-CoA |
|  | Octacosapentaenoyl-CoA |
|  | Hexacosahexaenoyl-CoA |
|  | Octacosahexaenoyl-CoA |
|  | Hexacosatetraenoyl-CoA |
|  | Octacosatetraenoyl-CoA |
|  | Hexacosapentaenoyl-CoA |
|  | Octacosapentaenoyl-CoA |
|  | Hexadecadienoyl-CoA |
|  |  |
| Renin-angiotensin system | Angiotensin I |
|  | Angiotensin II |
|  | Angiotensin III |
|  | Angiotensin IV |
|  | Angiotensin (1-7) |
|  | Angiotensin (1-9) |
|  | Angiotensin (1-5) |
|  | Angiotensin A |
|  | Alamandine |
|  | Angiotensin (5-8) |
|  | Angiotensin (5-7) |
|  |  |
| Glycerolipid metabolism | Glycerone phosphate |
|  | Acyl-[acyl-carrier protein] |
|  | Glycerone |
|  | D-Glycerate |
|  | Triacylglycerol |
|  | D-Glyceraldehyde |
|  | 1,2-Diacyl-sn-glycerol |
|  | 1-Acyl-sn-glycerol 3-phosphate |
|  | 3-Hydroxypropanal |
|  | 1-Acylglycerol |
|  | Acyl phosphate |
|  | Propane-1,3-diol |
|  | D-Galactosyldiacylglycerol |
|  | 3-D-Glucosyl-1,2-diacylglycerol |
|  | 2-Acyl-3-O-(beta-D-galactosyl)-sn-glycero |
|  | 1,2-Diacyl-3-[3-(alpha-D-N-acetylneuraminyl)-beta-D-galactosyl]-sn-glycerol |
|  | Galactosylglycerol |
|  | Digalactosyl-diacylglycerol |
|  | Acyl1-monogalactosyl-diacylglycerol |
|  | Diglucosyldiacylglycerol |
|  | Glycerophosphoglycoglycerolipid |
|  | Lipoteichoic acid |
|  | 1,2-Diacyl-3-alpha-D-glucosyl-sn-glycerol |
|  | alpha-Kojibiosyldiacylglycero |
|  | UDP-6-sulfoquinovose |
|  | Sulfoquinovosyldiacylglycerol |
|  | Glycerophosphoglycoglycerolipid |
|  | Lipoteichoic acid |
|  | 1,2-Diacyl-3-O-[beta-D-galactosyl-(1->6)-beta-D-galactosyl]-sn-glycerol |
|  |  |
| Fatty acid elongation | Butanoyl-CoA |
|  | Palmitoyl-CoA |
|  | Hexadecanoic acid |
|  | 3-Oxoacyl-CoA |
|  | 2,3-Dehydroacyl-CoA |
|  | Long-chain fatty acid |
|  | (3S)-3-Hydroxyacyl-CoA |
|  | trans-2,3-Dehydroacyl-CoA |
|  | Lauroyl-CoA; Lauroyl coenzyme A |
|  | Octanoyl-CoA |
|  | Tetradecanoyl-CoA |
|  | Long-chain acyl-CoA |
|  | 2-trans-Dodecenoyl-CoA |
|  | (S)-3-Hydroxyhexadecanoyl-CoA |
|  | 3-Oxopalmitoyl-CoA |
|  | (S)-3-Hydroxytetradecanoyl-CoA |
|  | 3-Oxotetradecanoyl-CoA |
|  | (S)-3-Hydroxydodecanoyl-CoA |
|  | 3-Oxododecanoyl-CoA |
|  | (S)-Hydroxydecanoyl-CoA |
|  | 3-Oxodecanoyl-CoA(S)-3-Hydroxyoctanoyl-CoA |
|  | 3-Oxooctanoyl-CoA |
|  | (S)-Hydroxyhexanoyl-CoA |
|  | 3-Oxohexanoyl-CoA |
|  | Hexanoyl-CoA |
|  | trans-Hex-2-enoyl-CoA |
|  | trans-Hexadec-2-enoyl-CoA |
|  | trans-Tetradec-2-enoyl-CoA |
|  | Decanoyl-CoA |
|  | trans-Dec-2-enoyl-CoA |
|  | trans-Oct-2-enoyl-CoA |
|  | Very-long-chain acyl-CoA |
|  | Very-long-chain 3-oxoacyl-CoA |
|  | Very-long-chain (3R)-3-hydroxyacyl-CoA |
|  | Very-long-chain trans-2,3-dehydroacyl-CoA |
| Shigellosis | 1-Phosphatidyl-1D-myo-inositol 3-phosphate |
|  | 1-Phosphatidyl-D-myo-inositol 4,5-bisphosphate; |
|  | alpha-D-Galactosyl-(1->4)-beta-D-galactosyl-(1->4)-beta-D-glucosyl-(1<->1)-ceramide |
|  | D-glycero-beta-D-manno-Heptose 1,7-bisphosphate |
|  | 1-Phosphatidyl-1D-myo-inositol 3,5-bisphosphate |
|  | 1-Phosphatidyl-1D-myo-inositol 5-phosphate |
|  |  |
| Epithelial cell signaling in Helicobacter pylori infection | Hydron |
|  | NH4+ |
| Adipocytokine signaling pathway | N-Acylsphingosine |
|  | Long-chain fatty acid |
|  |  |
| Pentose phosphate pathway | D-Glyceraldehyde 3-phosphate |
|  | 5-Phospho-alpha-D-ribose 1-diphosphate |
|  | 3-Phospho-D-glycerat |
|  | D-Glucono-1,5-lactone |
|  | D-Ribulose 5-phosphate |
|  | 2-Dehydro-3-deoxy-D-gluconate |
|  | beta-D-Glucose |
|  | D-Gluconic acid |
|  | D-Glycerate |
|  | D-Erythrose 4-phosphate |
|  | 6-Phospho-D-gluconate |
|  | D-Glyceraldehyde |
|  | alpha-D-Ribose 1-phosphate |
|  | 2-Deoxy-D-ribose 1-phosphate |
|  | 2-Deoxy-D-ribose 5-phosphate |
|  | D-Ribose 1,5-bisphosphate |
|  | 6-Phospho-2-dehydro-D-gluconate |
|  | D-Glucono-1,5-lactone 6-phosphate |
|  | Deoxyribose; 2-Deoxy-D-erythro-pentose |
|  | 2-Amino-2-deoxy-D-gluconate |
|  | beta-D-Fructose 6-phosphate |
|  | 2-Dehydro-3-deoxy-6-phospho-D-gluconate |
|  | beta-D-Fructose 1,6-bisphosphate |
|  | Sedoheptulose 7-phosphate; D-Sedoheptulose 7-phosphate |
|  | D-arabino-Hex-3-ulose 6-phosphate |
|  | 2-Keto-D-gluconic acid |
|  | D-Glucosaminate-6-phosphate |
|  |  |
| Pertussis | Nicotinate |
|  | Heparin |
|  | Glycolipid |
|  | Magnesium sulfate |
|  | LOS |
|  |  |
| Hepatitis C | 2',5'-Oligoadenylate 5'-triphosphate |
|  |  |
| Pathogenic Escherichia coli infection | Phosphatidylethanolamine |
|  | 1-Acyl-sn-glycerol 3-phosphate |
|  |  |
| Long-term depression | Arachidonic acid |
|  | 1,2-Diacyl-sn-glycerol |
|  |  |
| VEGF signaling pathway | Prostaglandin I2 |
|  |  |
| Arachidonic acid metabolism | Phosphatidylcholine |
|  | Arachidonic acid |
|  | Prostaglandin H2 |
|  | Prostaglandin F2alpha |
|  | Leukotriene A4 |
|  | Prostaglandin I2 |
|  | Leukotriene B4 |
|  | Leukotriene C4 |
|  | Thromboxane A2 |
|  | 20-Hydroxyleukotriene E4 |
|  | 15-OxoETE |
|  | (15S)-15-Hydroxy-5,8,11-cis-13-trans-eicosatetraenoate |
|  | 5(S)-HETE |
|  | 8(R)-HPETE |
|  | 20-OH-Leukotriene B4; |
|  | 5(S)-HPETE |
|  | 12-Keto-leukotriene B4 |
|  | 20-COOH-Leukotriene B4 |
|  | Leukotriene D4 |
|  | Leukotriene E4 |
|  | Prostaglandin A2 |
|  | Prostaglandin B2 |
|  | Prostaglandin C2 |
|  | Prostaglandin G2 |
|  | Prostaglandin J2 |
|  | Delta-12-Prostaglandin J2 |
|  | 11-epi-Prostaglandin F2alpha |
|  | 15-Keto-prostaglandin F2alpha |
|  | 6-Keto-prostaglandin F1alpha |
|  | 6-Keto-prostaglandin E1 |
|  | Thromboxane B2 |
|  | 11-Dehydro-thromboxane B2 |
|  | 12(S)-HPETE |
|  | 15(S)-HPETE |
|  | Lipoxin A4 |
|  | Lipoxin B4 |
|  | Leukotriene F4 |
|  | 9,11,15-Trihydroxy-prosta-5,13-dien-1-oic acid |
|  | 15-Deoxy-Delta12,14-PGJ2; 15-Deoxy-Delta12,14-prostaglandin J2 |
|  | 5-OxoETE |
|  | 20-HETE |
|  | 19(S)-HETE |
|  | 5,6-EET |
|  | 8,9-EET |
|  | 11,12-EET |
|  | 14,15-EET |
|  | 5,6-DHET |
|  | 8,9-DHET |
|  | 11,12-DHET |
|  | 14,15-DHET |
|  | 8(S)-HETE |
|  | 12(S)-HETE |
|  | 16(R)-HETE |
|  | 9(S)-HETE |
|  | 11(R)-HETE |
|  | 15H-11,12-EETA |
|  | 11,12,15-THETA |
|  | 2,3-Dinor-8-iso prostaglandin F2alpha |
|  | 2,3-Dinor-8-iso prostaglandin F1alpha |
|  | 12-OxoETE |
|  | Hepoxilin A3 |
|  | Trioxilin A3 |
|  | Hepoxilin B3 |
|  | Trioxilin B3 |
|  | 12(R)-HPETE |
|  | 11H-14,15-EETA |
|  | 11,14,15-THETA |
|  | 5,6-Epoxytetraene |
|  | 11(R)-HPETE |
|  | 9(S)-HPETE |
|  | 12(R)-HETE |
|  | 8(S)-HPETE |
|  | 8(R)-HETE |
|  |  |
| Renin secretion | Adenosin |
|  | Noradrenaline |
|  | Adrenaline |
|  | Angiotensin I |
|  | Angiotensin II |
|  |  |
| Glycolysis / Gluconeogenesis | Acetate |
|  | Acetaldehyde |
|  | Glycerone phosphate |
|  | D-Glyceraldehyde 3-phosphate |
|  | L-Lactic acid |
|  | beta-D-Glucose |
|  | 3-Phospho-D-glyceroyl phosphate |
|  | Ethanol |
|  | 2,3-Bisphospho-D-glycerate |
|  | Salicin |
|  | beta-D-Fructose 6-phosphate |
|  | beta-D-Fructose 1,6-bisphosphate |
|  | Arbutin |
|  | Arbutin 6-phosphate |
|  | Salicin 6-phosphate |
|  |  |
| Fc gamma R-mediated phagocytosis | Arachidonic acid |
|  | 1-Phosphatidyl-D-myo-inositol 4,5-bisphosphate |
|  | Sphingosine 1-phosphate |
|  |  |
| Glycerophospholipid metabolism | Acetaldehyde |
|  | Glycerone phosphate |
|  | Phosphatidylcholine |
|  | Ethanolamine |
|  | CDP-diacylglycerol |
|  | CDP-choline; Cytidine 5'-diphosphocholine |
|  | Phosphatidylglycerol |
|  | Ethanolamine phosphate |
|  | Phosphatidylethanolamine |
|  | CDP-glycerol |
|  | CDP-ethanolamine |
|  | Choline phosphate |
|  | L-Glycerol 1-phosphate |
|  | 1,2-Diacyl-sn-glycerol |
|  | Glycerophosphocholine |
|  | 1-Acyl-sn-glycerol 3-phosphate |
|  | 1-Phosphatidyl-D-myo-inositol |
|  | N-Methylethanolamine phosphate |
|  | Glycerophosphoethanolamine |
|  | Phosphatidyl-N-methylethanolamine |
|  | Phosphatidylserine |
|  | Acylglycerone phosphate |
|  | 1-Acylglycerophosphoinositol |
|  | L-Serine-phosphoethanolamine |
|  | Phosphatidylglycerophosphate |
|  | 2-Acyl-sn-glycerol 3-phosphate |
|  | 1-Acyl-sn-glycero-3-phosphocholine |
|  | 2-Acyl-sn-glycero-3-phosphocholine |
|  | Phosphatidyl-N-dimethylethanolamine |
|  | 1-Acyl-sn-glycero-3-phosphoethanolamine |
|  | 3-(O-Geranylgeranyl)-sn-glycerol 1-phosphate |
|  | 2,3-Bis-O-(geranylgeranyl)glycerol 1-phosphate |
|  | 2-Acyl-sn-glycero-3-phosphoethanolamine |
|  | 2-Acyl-sn-glycero-3-phosphoserine |
|  | Aminoacyl-phosphatidylglycerol |
|  | Cardiolipin |
|  | Triethanolamine |
|  | Diethanolamine |
|  | Phosphodimethylethanolamine |
|  | CDP-2,3-bis-O-(geranylgeranyl)-sn-glycero |
|  | 1-Acyl-sn-glycero-3-phosphoserine |
|  | 1-Acyl-sn-glycero-3-phosphoglycerol |
|  | Monolysocardiolipin |
|  | Diacylglycerylhomoserine |
|  | Diacylglyceryl-N,N,N-trimethylhomoserine |
|  | 2,3-Bis-O-(geranylgeranyl)-sn-glycero-1-phospho-L-serine |
|  | 2,3-Bis-(O-phytanyl)-sn-glycerol 1-phosphate |
|  |  |
| HIF-1 signaling pathway - Homo sapiens | Ascorbate |
|  | L-Lactic acid |
|  | O2.- |
|  |  |
| Insulin resistance - Homo sapiens | UDP-N-acetyl-alpha-D-glucosamine |
|  | N-Acylsphingosine |
|  | D-Glucosamine 6-phosphate |
|  | Triacylglycerol |
|  | Nitric oxide |
|  | O-Acetylcarnitine |
|  | Long-chain acyl-CoA |
|  | alpha-D-Glucosamine 1-phosphate |

**Table S18.** Common pathways between multi-source data integration approach and the pre-symptomatic and symptomatic HD

| **Pathways in multi-source data integration approach** | **Common pathways in Pre-symptomatic HD** | **Common pathways in symptomatic HD** |
| --- | --- | --- |
| Huntington’s disease |  | ✓ |
| Amyotrophic Lateral Sclerosis |  |  |
| Hepatitis B | ✓ | ✓ |
| AD |  | ✓ |
| Viral Carcinogenesis | ✓ |  |
| Herpes infection |  | ✓ |
| Apoptosis |  |  |
| Cocaine addiction | ✓ |  |
| Long term potentiation | ✓ |  |
| cAMP signaling | ✓ | ✓ |
| Tuberculosis |  |  |
| p53 signaling | ✓ |  |
| Circadian entrainment | ✓ | ✓ |
| HTLV-1 infection | ✓ | ✓ |
| Dopaminergic synapse | ✓ | ✓ |
| Pathways in Cancer | ✓ | ✓ |
| Alcoholism |  | ✓ |
| Legionellosis |  | ✓ |
| Colorectal cancer | ✓ | ✓ |
| Amphetamine addiction |  |  |
| MAPK signaling |  | ✓ |
| TGF-beta signaling | ✓ | ✓ |
| Wnt signaling | ✓ | ✓ |
| Cell cycle | ✓ | ✓ |
| Jak-STAT signaling | ✓ |  |
| PD |  | ✓ |
| Ca^2+^ signaling | ✓ |  |
| PI3K-Akt signaling | ✓ | ✓ |
| Glioma |  | ✓ |
| Non-small cell lung cancer | ✓ | ✓ |
| PPAR signaling |  | ✓ |
| VEGF signaling |  |  |
| Prolactin signaling pathway |  |  |

**Table S19.** Limma with adjusted p-value for pre-symptomatic HD

| **P value** | **LogFC** | **Adj p-value** | **Gene symbol** |
| --- | --- | --- | --- |
| 1.60E-06 | -2.86634 | 0.0278 | MIR1236 |
| 2.50E-06 | 2.16181 | 0.0078 | CCND1 |

**Table S20.** Limma with adjusted p-value for symptomatic HD

| **P value** | **LogFC** | **Adj p-value** | **Gene symbol** |
| --- | --- | --- | --- |
| 0.00167 | -2.20141 | 1.8E-02 | HLA-DQA1 |
| 0.00001 | -1.96714 | 4.4E-04 | C2CD2L |
| 0.00001 | -1.92138 | 4.4E-04 | UBE2O |
| 0.00254 | -1.88827 | 2.4E-02 | DKK2 |
| 0.00063 | -1.75275 | 8.4E-03 | GP1BB |
| 0.00001 | -1.70512 | 2.1E-04 | CNTN6 |
| 0.00001 | -1.68684 | 4.0E-04 | TECR |
| 0.00015 | -1.65747 | 2.8E-03 | FMO3 |
| 0.00023 | -1.65522 | 3.9E-03 | SEMA3E |
| 0.00002 | -1.63506 | 5.9E-04 | NENF |
| 0.00326 | -1.62883 | 2.9E-02 | AF198444 |
| 0.00032 | -1.59003 | 4.9E-03 | ADRA2A |
| 0.00521 | -1.58702 | 3.9E-02 | RFPL1 |
| 0.00007 | -1.57366 | 1.6E-03 | TCF3 |
| 0.00004 | -1.57188 | 1.0E-03 | ZNF652 |
| 0.00010 | -1.55836 | 2.0E-03 | DNAJB2 |
| 0.00678 | -1.55126 | 4.7E-02 | ANKRD7 |
| 0.00083 | -1.54836 | 1.0E-02 | SRPX2 |
| 0.00645 | -1.53960 | 4.6E-02 | RGS13 |
| 0.00001 | -1.53673 | 2.5E-04 | ZIC4 |
| 0.00096 | -1.52283 | 1.2E-02 | SLC4A8 |
| 0.00007 | -1.49093 | 1.6E-03 | APBA1 |
| 0.00512 | -1.42877 | 3.9E-02 | LPA |
| 0.00008 | -1.40677 | 1.7E-03 | TTC27 |
| 0.00028 | -1.40671 | 4.5E-03 | MAFK |
| 0.00013 | -1.40535 | 2.5E-03 | VPS39 |
| 0.00001 | -1.39853 | 4.3E-04 | LRRC17 |
| 0.00360 | -1.39536 | 3.0E-02 | PTOV1 |
| 0.00520 | -1.38726 | 3.9E-02 | OR7E156P |
| 0.00130 | -1.38049 | 1.5E-02 | HAPLN2 |
| 0.00406 | -1.37400 | 3.3E-02 | PDGFA |
| 0.00657 | -1.33105 | 4.6E-02 | RPGRIP1L |
| 0.00012 | -1.32955 | 2.4E-03 | ADORA2A |
| 0.00103 | -1.32883 | 1.2E-02 | CLU |
| 0.00100 | -1.32812 | 1.2E-02 | KCNJ5 |
| 0.00206 | -1.32103 | 2.0E-02 | PDZD2 |
| 0.00006 | -1.31968 | 1.4E-03 | GPD1 |
| 0.00113 | -1.31873 | 1.3E-02 | PBXIP1 |
| 0.00001 | -1.31286 | 2.4E-04 | RXRA |
| 0.00022 | -1.31177 | 3.8E-03 | BRPF1 |
| 0.00024 | -1.31164 | 4.1E-03 | CDKN1C |
| 0.00087 | -1.30804 | 1.1E-02 | AKR1B10 |
| 0.00007 | -1.30283 | 1.5E-03 | FKBP8 |
| 0.00194 | -1.28276 | 2.0E-02 | TCF7L2 |
| 0.00658 | -1.27767 | 4.6E-02 | LOC100506282 |
| 0.00310 | -1.27509 | 2.7E-02 | TMPRSS5 |
| 0.00001 | -1.26568 | 4.0E-04 | ASCC2 |
| 0.00406 | -1.26347 | 3.3E-02 | CTRB1 |
| 0.00083 | -1.25011 | 1.0E-02 | PP14571 |
| 0.00022 | -1.24580 | 3.7E-03 | PNPLA2 |
| 0.00025 | -1.24531 | 4.2E-03 | ANKZF1 |
| 0.00301 | -1.24478 | 2.7E-02 | CPS1-IT1 |
| 0.00126 | -1.24335 | 1.4E-02 | HSPB8 |
| 0.00206 | -1.23869 | 2.0E-02 | TPM2 |
| 0.00382 | -1.23541 | 3.2E-02 | LARGE |
| 0.00438 | -1.23476 | 3.5E-02 | PVALB |
| 0.00021 | -1.22599 | 3.6E-03 | F5 |
| 0.00725 | -1.22559 | 5.0E-02 | RP11-15P13.1 |
| 0.00182 | -1.22481 | 1.9E-02 | SHARPIN |
| 0.00139 | -1.21937 | 1.5E-02 | MAT1A |
| 0.00008 | -1.21908 | 1.7E-03 | PARD3 |
| 0.00000 | -1.21067 | 5.7E-05 | FOXO3 |
| 0.00505 | -1.20808 | 3.9E-02 | TMSB4Y |
| 0.00554 | -1.20053 | 4.1E-02 | POU3F2 |
| 0.00019 | -1.19922 | 3.4E-03 | CACNA1E |
| 0.00372 | -1.19807 | 3.1E-02 | MYOD1 |
| 0.00002 | -1.19802 | 6.4E-04 | NRGN |
| 0.00045 | -1.19801 | 6.5E-03 | TSSC4 |
| 0.00236 | -1.18858 | 2.3E-02 | WISP1 |
| 0.00436 | -1.18267 | 3.5E-02 | HYAL1 |
| 0.00614 | -1.18196 | 4.4E-02 | GPR22 |
| 0.00155 | -1.18180 | 1.6E-02 | SF3A2 |
| 0.00000 | -1.17924 | 9.1E-05 | RPLP2 |
| 0.00127 | -1.16658 | 1.4E-02 | CHERP |
| 0.00514 | -1.16504 | 3.9E-02 | ADIRF |
| 0.00215 | -1.16418 | 2.1E-02 | F2RL3 |
| 0.00477 | -1.16142 | 3.7E-02 | CYP2B7P |
| 0.00656 | -1.15840 | 4.6E-02 | TSGA10 |
| 0.00519 | -1.15643 | 3.9E-02 | HOXA11 |
| 0.00467 | -1.15223 | 3.6E-02 | CTDSPL |
| 0.00006 | -1.14423 | 1.3E-03 | FARSA |
| 0.00381 | -1.13863 | 3.2E-02 | MEA1 |
| 0.00420 | -1.13564 | 3.4E-02 | CCNT1 |
| 0.00017 | -1.13484 | 3.0E-03 | BRD4 |
| 0.00286 | -1.12826 | 2.6E-02 | STAG3L3 |
| 0.00157 | -1.12645 | 1.7E-02 | SPACA1 |
| 0.00027 | -1.12125 | 4.4E-03 | NEUROD6 |
| 0.00306 | -1.11853 | 2.7E-02 | ARPC4 |
| 0.00209 | -1.11756 | 2.1E-02 | SLC6A9 |
| 0.00060 | -1.11562 | 8.1E-03 | SIT1 |
| 0.00024 | -1.11010 | 4.0E-03 | CLPB |
| 0.00519 | -1.10483 | 3.9E-02 | SCGB2A1 |
| 0.00504 | -1.10354 | 3.9E-02 | SHCBP1L |
| 0.00099 | -1.10339 | 1.2E-02 | ACKR1 |
| 0.00315 | -1.10331 | 2.8E-02 | LOC101060747 |
| 0.00059 | -1.10046 | 8.0E-03 | AQP3 |
| 0.00492 | -1.09794 | 3.8E-02 | DDX49 |
| 0.00701 | -1.09775 | 4.8E-02 | DHRS2 |
| 0.00452 | -1.08650 | 3.6E-02 | ANGPT2 |
| 0.00215 | -1.08345 | 2.1E-02 | CELA3B |
| 0.00291 | -1.08137 | 2.6E-02 | SLC48A1 |
| 0.00084 | -1.07693 | 1.1E-02 | KDM2A |
| 0.00585 | -1.07609 | 4.3E-02 | GUCY1A2 |
| 0.00050 | -1.07157 | 7.0E-03 | SEMA6C |
| 0.00004 | -1.06859 | 9.7E-04 | MAP4K1 |
| 0.00456 | -1.06682 | 3.6E-02 | RUNX1-IT1 |
| 0.00718 | -1.06508 | 4.9E-02 | PRPF6 |
| 0.00003 | -1.06155 | 8.0E-04 | RPL27A |
| 0.00172 | -1.05959 | 1.8E-02 | DNAJC12 |
| 0.00289 | -1.05681 | 2.6E-02 | CLDN17 |
| 0.00126 | -1.05308 | 1.4E-02 | SGCB |
| 0.00390 | -1.04939 | 3.2E-02 | C14orf79 |
| 0.00655 | -1.04585 | 4.6E-02 | MATN1 |
| 0.00014 | -1.03625 | 2.6E-03 | CD248 |
| 0.00088 | -1.03617 | 1.1E-02 | PRDX6 |
| 0.00450 | -1.02865 | 3.6E-02 | SLC25A23 |
| 0.00255 | -1.02824 | 2.4E-02 | DSPP |
| 0.00002 | -1.02736 | 4.8E-04 | BAG6 |
| 0.00519 | -1.02613 | 3.9E-02 | SLC39A8 |
| 0.00116 | -1.02408 | 1.3E-02 | MLANA |
| 0.00687 | -1.02162 | 4.8E-02 | EHMT2 |
| 0.00445 | -1.01862 | 3.5E-02 | ECSIT |
| 0.00105 | -1.01473 | 1.2E-02 | ITFG2 |
| 0.00073 | -1.01401 | 9.4E-03 | CD5 |
| 0.00022 | -1.01307 | 3.7E-03 | SLC5A1 |
| 0.00476 | -1.01199 | 3.7E-02 | ZNF460 |
| 0.00000 | -1.00959 | 2.2E-05 | ACTN4 |
| 0.00063 | -1.00926 | 8.4E-03 | PF4 |
| 0.00317 | -1.00797 | 2.8E-02 | PRR34 |
| 0.00200 | -1.00775 | 2.0E-02 | ZNF235 |
| 0.00338 | -1.00528 | 2.9E-02 | TNXB |
| 0.00207 | -1.00111 | 2.1E-02 | PACS2 |
| 0.00103 | -1.00063 | 1.2E-02 | ZNF135 |
| 0.00616 | -0.99843 | 4.4E-02 | PICK1 |
| 0.00219 | -0.99797 | 2.1E-02 | PRKCE |
| 0.00544 | -0.99422 | 4.1E-02 | SLC35D1 |
| 0.00277 | -0.99123 | 2.5E-02 | RBMXL2 |
| 0.00002 | -0.99080 | 4.9E-04 | NCF1 |
| 0.00243 | -0.98880 | 2.3E-02 | TRIM62 |
| 0.00682 | -0.98409 | 4.8E-02 | HR |
| 0.00076 | -0.98361 | 9.7E-03 | SLC17A9 |
| 0.00437 | -0.97953 | 3.5E-02 | HMBOX1 |
| 0.00725 | -0.97842 | 5.0E-02 | CES1P1 |
| 0.00285 | -0.97475 | 2.6E-02 | CLUH |
| 0.00153 | -0.97084 | 1.6E-02 | RPL10 |
| 0.00437 | -0.96270 | 3.5E-02 | MBD3 |
| 0.00000 | -0.96268 | 1.4E-04 | FKSG49 |
| 0.00000 | -0.96170 | 8.0E-05 | LYL1 |
| 0.00189 | -0.95758 | 1.9E-02 | RIMS3 |
| 0.00157 | -0.95754 | 1.7E-02 | GRIA3 |
| 0.00556 | -0.95305 | 4.1E-02 | DZIP1 |
| 0.00708 | -0.95195 | 4.9E-02 | YPEL1 |
| 0.00163 | -0.95045 | 1.7E-02 | TNNC1 |
| 0.00015 | -0.94588 | 2.8E-03 | CDH11 |
| 0.00224 | -0.94530 | 2.2E-02 | DPH2 |
| 0.00534 | -0.94513 | 4.0E-02 | APOL1 |
| 0.00338 | -0.93473 | 2.9E-02 | H6PD |
| 0.00533 | -0.93208 | 4.0E-02 | CAMK1G |
| 0.00209 | -0.93117 | 2.1E-02 | RBM38 |
| 0.00054 | -0.92815 | 7.4E-03 | CARM1 |
| 0.00317 | -0.92788 | 2.8E-02 | NEU3 |
| 0.00567 | -0.92711 | 4.2E-02 | DNM1 |
| 0.00059 | -0.92495 | 8.0E-03 | ARHGAP44 |
| 0.00613 | -0.92472 | 4.4E-02 | CDKL5 |
| 0.00146 | -0.92201 | 1.6E-02 | FZD8 |
| 0.00005 | -0.92004 | 1.1E-03 | PRR5 |
| 0.00665 | -0.91984 | 4.7E-02 | UCHL1 |
| 0.00269 | -0.91467 | 2.5E-02 | COPZ2 |
| 0.00036 | -0.91402 | 5.4E-03 | PHACTR4 |
| 0.00268 | -0.90981 | 2.5E-02 | HLA-DPA2 |
| 0.00000 | -0.90971 | 8.1E-06 | ALDOA |
| 0.00697 | -0.90454 | 4.8E-02 | KRT84 |
| 0.00222 | -0.90433 | 2.2E-02 | SLC19A1 |
| 0.00019 | -0.90377 | 3.3E-03 | ATP6V0C |
| 0.00696 | -0.90338 | 4.8E-02 | OR1F2P |
| 0.00004 | -0.89730 | 1.0E-03 | MBOAT7 |
| 0.00311 | -0.89065 | 2.7E-02 | LMNA |
| 0.00164 | -0.89005 | 1.7E-02 | CELSR3 |
| 0.00333 | -0.88841 | 2.9E-02 | DBN1 |
| 0.00329 | -0.88562 | 2.9E-02 | ALX4 |
| 0.00163 | -0.88238 | 1.7E-02 | PSMD11 |
| 0.00226 | -0.88148 | 2.2E-02 | ARHGEF17 |
| 0.00346 | -0.87944 | 3.0E-02 | CEP250 |
| 0.00714 | -0.87794 | 4.9E-02 | LZTS1 |
| 0.00204 | -0.87694 | 2.0E-02 | OPHN1 |
| 0.00000 | -0.87504 | 2.0E-04 | CD74 |
| 0.00001 | -0.87225 | 2.5E-04 | KPNA6 |
| 0.00247 | -0.87196 | 2.3E-02 | GLG1 |
| 0.00115 | -0.87095 | 1.3E-02 | CABP1 |
| 0.00110 | -0.86947 | 1.3E-02 | MCAM |
| 0.00088 | -0.86895 | 1.1E-02 | LOC732360 |
| 0.00228 | -0.86527 | 2.2E-02 | TNPO2 |
| 0.00027 | -0.86325 | 4.4E-03 | TCF25 |
| 0.00232 | -0.86276 | 2.2E-02 | RENBP |
| 0.00022 | -0.86251 | 3.7E-03 | FARP2 |
| 0.00711 | -0.86242 | 4.9E-02 | RPS6KB2 |
| 0.00222 | -0.86229 | 2.2E-02 | CHIT1 |
| 0.00564 | -0.86216 | 4.2E-02 | PTDSS2 |
| 0.00046 | -0.85869 | 6.6E-03 | LOC101060275 |
| 0.00135 | -0.85432 | 1.5E-02 | RPS11 |
| 0.00030 | -0.85383 | 4.8E-03 | CD79A |
| 0.00018 | -0.85375 | 3.1E-03 | SORL1 |
| 0.00700 | -0.85258 | 4.8E-02 | MZB1 |
| 0.00294 | -0.85239 | 2.6E-02 | PSCA |
| 0.00529 | -0.85219 | 4.0E-02 | LRP1 |
| 0.00021 | -0.85071 | 3.6E-03 | NLRX1 |
| 0.00296 | -0.84744 | 2.7E-02 | CHAC1 |
| 0.00000 | -0.84692 | 9.9E-05 | ENO1 |
| 0.00138 | -0.84526 | 1.5E-02 | MED22 |
| 0.00495 | -0.84494 | 3.8E-02 | RUVBL2 |
| 0.00338 | -0.84329 | 2.9E-02 | RHOT2 |
| 0.00039 | -0.84223 | 5.8E-03 | ADAM11 |
| 0.00726 | -0.84213 | 5.0E-02 | ALDH1A2 |
| 0.00062 | -0.83321 | 8.3E-03 | THEG |
| 0.00484 | -0.83260 | 3.7E-02 | ESRP2 |
| 0.00005 | -0.83255 | 1.3E-03 | NCF1 |
| 0.00192 | -0.83079 | 1.9E-02 | PRRC2A |
| 0.00683 | -0.82867 | 4.8E-02 | UQCRC1 |
| 0.00301 | -0.82682 | 2.7E-02 | CEP104 |
| 0.00164 | -0.82672 | 1.7E-02 | EMID1 |
| 0.00106 | -0.82359 | 1.2E-02 | RPH3A |
| 0.00058 | -0.82351 | 7.9E-03 | GAST |
| 0.00000 | -0.82315 | 6.8E-05 | RHOG |
| 0.00244 | -0.82094 | 2.3E-02 | CDKN3 |
| 0.00004 | -0.81988 | 9.9E-04 | RP11-403P17.4 |
| 0.00182 | -0.81632 | 1.9E-02 | MAGEA8 |
| 0.00030 | -0.81607 | 4.7E-03 | SH3BGRL3 |
| 0.00002 | -0.81452 | 5.5E-04 | CCND3 |
| 0.00663 | -0.81401 | 4.7E-02 | ROBO3 |
| 0.00250 | -0.81362 | 2.3E-02 | ENPP1 |
| 0.00010 | -0.81327 | 2.1E-03 | HK1 |
| 0.00000 | -0.80795 | 3.9E-05 | PFN1 |
| 0.00053 | -0.80756 | 7.4E-03 | PRF1 |
| 0.00031 | -0.80495 | 4.9E-03 | RPL38 |
| 0.00034 | -0.80311 | 5.2E-03 | RALY |
| 0.00609 | -0.80242 | 4.4E-02 | LOC100506699 |
| 0.00466 | -0.80099 | 3.6E-02 | SPINK2 |
| 0.00558 | -0.79967 | 4.1E-02 | RP11-665C16.8 |
| 0.00398 | -0.79767 | 3.3E-02 | SPINT1 |
| 0.00019 | -0.79762 | 3.3E-03 | LOC100996792 |
| 0.00019 | -0.79756 | 3.3E-03 | SMARCB1 |
| 0.00196 | -0.79723 | 2.0E-02 | WDR18 |
| 0.00279 | -0.79691 | 2.5E-02 | CUEDC1 |
| 0.00141 | -0.79275 | 1.5E-02 | CD5L |
| 0.00020 | -0.79177 | 3.5E-03 | FMNL1 |
| 0.00046 | -0.79122 | 6.6E-03 | WDR6 |
| 0.00003 | -0.78946 | 7.6E-04 | JARID2 |
| 0.00032 | -0.78761 | 5.0E-03 | PPDPF |
| 0.00128 | -0.78584 | 1.4E-02 | RP4-781L3.1 |
| 0.00095 | -0.78382 | 1.1E-02 | BAG1 |
| 0.00002 | -0.78001 | 5.5E-04 | TEC |
| 0.00239 | -0.77545 | 2.3E-02 | TESC |
| 0.00493 | -0.77369 | 3.8E-02 | ELN |
| 0.00700 | -0.77113 | 4.8E-02 | GRP |
| 0.00146 | -0.77000 | 1.6E-02 | PIGQ |
| 0.00000 | -0.76851 | 1.6E-04 | EMP3 |
| 0.00105 | -0.76762 | 1.2E-02 | SLC16A8 |
| 0.00114 | -0.76731 | 1.3E-02 | MAP2K2 |
| 0.00071 | -0.76690 | 9.2E-03 | DHDDS |
| 0.00345 | -0.75981 | 3.0E-02 | BCR |
| 0.00640 | -0.75944 | 4.6E-02 | FADS3 |
| 0.00002 | -0.75897 | 5.9E-04 | NFE2 |
| 0.00349 | -0.75594 | 3.0E-02 | DCHS1 |
| 0.00115 | -0.75499 | 1.3E-02 | SAMD4B |
| 0.00033 | -0.75436 | 5.0E-03 | PROZ |
| 0.00221 | -0.75326 | 2.1E-02 | ADAM5 |
| 0.00025 | -0.75227 | 4.2E-03 | FXR2 |
| 0.00049 | -0.75084 | 6.9E-03 | EXOSC4 |
| 0.00002 | -0.74955 | 5.6E-04 | FLNA |
| 0.00032 | -0.74952 | 4.9E-03 | NFAT5 |
| 0.00613 | -0.74779 | 4.4E-02 | SARM1 |
| 0.00055 | -0.74742 | 7.6E-03 | NOTCH2NL |
| 0.00056 | -0.74733 | 7.6E-03 | PDE6G |
| 0.00132 | -0.74729 | 1.5E-02 | PIWIL2 |
| 0.00539 | -0.74727 | 4.0E-02 | PSMF1 |
| 0.00451 | -0.74418 | 3.6E-02 | CLCN7 |
| 0.00001 | -0.74361 | 2.7E-04 | CSK |
| 0.00156 | -0.74297 | 1.7E-02 | ESPN |
| 0.00074 | -0.74207 | 9.5E-03 | AP2S1 |
| 0.00539 | -0.74079 | 4.0E-02 | GPN2 |
| 0.00061 | -0.73983 | 8.2E-03 | SERPINA1 |
| 0.00108 | -0.73849 | 1.3E-02 | CHPF |
| 0.00017 | -0.73835 | 3.0E-03 | WWOX |
| 0.00028 | -0.73542 | 4.6E-03 | LOC101060373 |
| 0.00559 | -0.73526 | 4.1E-02 | VPS51 |
| 0.00080 | -0.73307 | 1.0E-02 | SLC26A10 |
| 0.00128 | -0.73276 | 1.4E-02 | GPR98 |
| 0.00047 | -0.73275 | 6.6E-03 | ABHD4 |
| 0.00182 | -0.73234 | 1.9E-02 | ROM1 |
| 0.00586 | -0.72987 | 4.3E-02 | DKK4 |
| 0.00007 | -0.72895 | 1.6E-03 | DIAPH1 |
| 0.00164 | -0.72823 | 1.7E-02 | GYPC |
| 0.00036 | -0.72783 | 5.4E-03 | SH3TC1 |
| 0.00000 | -0.72622 | 8.5E-05 | KAT6A |
| 0.00072 | -0.72296 | 9.3E-03 | UCP2 |
| 0.00009 | -0.72278 | 1.8E-03 | CST3 |
| 0.00604 | -0.72165 | 4.4E-02 | SNCAIP |
| 0.00326 | -0.72133 | 2.8E-02 | SPATA20 |
| 0.00510 | -0.72032 | 3.9E-02 | PRRX2 |
| 0.00342 | -0.71840 | 2.9E-02 | TMEM151B |
| 0.00383 | -0.71795 | 3.2E-02 | DIP2A |
| 0.00589 | -0.71713 | 4.3E-02 | PRSS3P3 |
| 0.00000 | -0.71598 | 6.6E-05 | FXYD5 |
| 0.00001 | -0.71503 | 2.3E-04 | AARS |
| 0.00007 | -0.71297 | 1.5E-03 | UBA1 |
| 0.00193 | -0.71154 | 2.0E-02 | LSP1 |
| 0.00084 | -0.70735 | 1.1E-02 | ARID3A |
| 0.00035 | -0.70706 | 5.3E-03 | MYH9 |
| 0.00276 | -0.70654 | 2.5E-02 | HBQ1 |
| 0.00355 | -0.70616 | 3.0E-02 | SEC31B |
| 0.00400 | -0.70547 | 3.3E-02 | MID1IP1 |
| 0.00014 | -0.70515 | 2.7E-03 | PLCG1 |
| 0.00023 | -0.70322 | 3.8E-03 | ARHGEF4 |
| 0.00037 | -0.70214 | 5.5E-03 | AP2M1 |
| 0.00124 | -0.70169 | 1.4E-02 | GATAD2A |
| 0.00276 | -0.70141 | 2.5E-02 | AF007147 |
| 0.00687 | -0.70126 | 4.8E-02 | H3F3A |
| 0.00341 | -0.69855 | 2.9E-02 | LGI1 |
| 0.00106 | -0.69819 | 1.2E-02 | ECE1 |
| 0.00376 | -0.69638 | 3.1E-02 | DPM2 |
| 0.00063 | -0.69351 | 8.4E-03 | SH2D2A |
| 0.00153 | -0.69256 | 1.6E-02 | SUPT6H |
| 0.00551 | -0.69106 | 4.1E-02 | OR51E2 |
| 0.00168 | -0.69079 | 1.8E-02 | ZMYM3 |
| 0.00051 | -0.68931 | 7.1E-03 | NCR2 |
| 0.00237 | -0.68888 | 2.3E-02 | INSL3 |
| 0.00001 | -0.68564 | 3.8E-04 | TNFRSF1B |
| 0.00037 | -0.68401 | 5.5E-03 | NDST1 |
| 0.00140 | -0.68389 | 1.5E-02 | RAPGEF2 |
| 0.00012 | -0.68134 | 2.3E-03 | GPSM3 |
| 0.00604 | -0.67869 | 4.4E-02 | CTSV |
| 0.00006 | -0.67798 | 1.4E-03 | GSTM2 |
| 0.00499 | -0.67705 | 3.8E-02 | FAM124B |
| 0.00455 | -0.67628 | 3.6E-02 | VIPR2 |
| 0.00017 | -0.67462 | 3.0E-03 | MAP3K11 |
| 0.00024 | -0.67306 | 3.9E-03 | TLN1 |
| 0.00525 | -0.67296 | 4.0E-02 | GPR135 |
| 0.00063 | -0.67192 | 8.4E-03 | FAM193B |
| 0.00647 | -0.67156 | 4.6E-02 | HOXD13 |
| 0.00018 | -0.67137 | 3.2E-03 | AAK1 |
| 0.00008 | -0.66952 | 1.7E-03 | RAB8A |
| 0.00513 | -0.66947 | 3.9E-02 | IFITM3 |
| 0.00015 | -0.66749 | 2.8E-03 | GTPBP6 |
| 0.00178 | -0.66744 | 1.8E-02 | PNPO |
| 0.00466 | -0.66734 | 3.6E-02 | CXCR1 |
| 0.00628 | -0.66727 | 4.5E-02 | MAP1S |
| 0.00061 | -0.66696 | 8.2E-03 | H2AFB1 |
| 0.00280 | -0.66683 | 2.5E-02 | BLMH |
| 0.00598 | -0.66609 | 4.4E-02 | NPHS1 |
| 0.00249 | -0.66607 | 2.3E-02 | PLA2G6 |
| 0.00086 | -0.66427 | 1.1E-02 | CYP1B1 |
| 0.00227 | -0.66414 | 2.2E-02 | RNH1 |
| 0.00377 | -0.66356 | 3.1E-02 | PAK4 |
| 0.00133 | -0.66318 | 1.5E-02 | PDLIM1 |
| 0.00055 | -0.66298 | 7.6E-03 | FKBP5 |
| 0.00025 | -0.66207 | 4.1E-03 | MAN2C1 |
| 0.00469 | -0.66111 | 3.7E-02 | CALB2 |
| 0.00231 | -0.66090 | 2.2E-02 | VAMP2 |
| 0.00033 | -0.66056 | 5.0E-03 | CACNB2 |
| 0.00423 | -0.65732 | 3.4E-02 | HAND2 |
| 0.00004 | -0.65446 | 1.0E-03 | CDC25B |
| 0.00718 | -0.65333 | 4.9E-02 | MUC5B |
| 0.00218 | -0.65245 | 2.1E-02 | SGTA |
| 0.00105 | -0.65151 | 1.2E-02 | STAT5A |
| 0.00005 | -0.65144 | 1.1E-03 | SLC7A7 |
| 0.00235 | -0.65059 | 2.2E-02 | PAX4 |
| 0.00432 | -0.65049 | 3.5E-02 | RBM12B-AS1 |
| 0.00004 | -0.65028 | 9.8E-04 | HDAC5 |
| 0.00210 | -0.64665 | 2.1E-02 | CABIN1 |
| 0.00328 | -0.64485 | 2.9E-02 | B3GNT4 |
| 0.00043 | -0.64297 | 6.3E-03 | CPNE1 |
| 0.00485 | -0.64213 | 3.8E-02 | SRRM1 |
| 0.00014 | -0.64053 | 2.6E-03 | PTPN6 |
| 0.00013 | -0.63679 | 2.5E-03 | LARP1 |
| 0.00290 | -0.63356 | 2.6E-02 | CAPNS1 |
| 0.00087 | -0.63291 | 1.1E-02 | CELSR2 |
| 0.00268 | -0.63205 | 2.5E-02 | PPP2R1A |
| 0.00676 | -0.63193 | 4.7E-02 | TSPAN9 |
| 0.00032 | -0.63073 | 5.0E-03 | FAM134A |
| 0.00022 | -0.62904 | 3.7E-03 | OS9 |
| 0.00045 | -0.62844 | 6.4E-03 | C15orf39 |
| 0.00711 | -0.62800 | 4.9E-02 | GP1BA |
| 0.00039 | -0.62789 | 5.8E-03 | ANXA11 |
| 0.00168 | -0.62702 | 1.8E-02 | HDAC6 |
| 0.00609 | -0.62645 | 4.4E-02 | PRR11 |
| 0.00150 | -0.62473 | 1.6E-02 | STRA6 |
| 0.00515 | -0.62463 | 3.9E-02 | CBX8 |
| 0.00425 | -0.62354 | 3.4E-02 | CYFIP2 |
| 0.00614 | -0.62343 | 4.4E-02 | EFCAB1 |
| 0.00454 | -0.62317 | 3.6E-02 | INTS5 |
| 0.00561 | -0.62292 | 4.2E-02 | TEAD4 |
| 0.00011 | -0.62128 | 2.3E-03 | UBA52 |
| 0.00062 | -0.62054 | 8.3E-03 | TAPBP |
| 0.00183 | -0.61972 | 1.9E-02 | SNTA1 |
| 0.00358 | -0.61816 | 3.0E-02 | TMEM59L |
| 0.00721 | -0.61715 | 4.9E-02 | LHX3 |
| 0.00419 | -0.61528 | 3.4E-02 | WBP2 |
| 0.00026 | -0.61438 | 4.3E-03 | SLC35G2 |
| 0.00629 | -0.61427 | 4.5E-02 | OBSCN |
| 0.00044 | -0.60976 | 6.4E-03 | SMARCD2 |
| 0.00030 | -0.60641 | 4.7E-03 | STAT6 |
| 0.00012 | -0.60539 | 2.3E-03 | PTPN1 |
| 0.00452 | -0.60358 | 3.6E-02 | CD97 |
| 0.00049 | -0.60351 | 6.9E-03 | CSRP3 |
| 0.00625 | -0.60347 | 4.5E-02 | ALPP |
| 0.00524 | -0.60279 | 4.0E-02 | FOXO1 |
| 0.00019 | -0.60270 | 3.4E-03 | FLOT2 |
| 0.00610 | -0.60158 | 4.4E-02 | COL11A2 |
| 0.00657 | -0.59907 | 4.6E-02 | LINC00483 |
| 0.00055 | -0.59896 | 7.6E-03 | FOXN3 |
| 0.00120 | -0.59813 | 1.4E-02 | PITPNM3 |
| 0.00328 | -0.59762 | 2.9E-02 | NASP |
| 0.00132 | -0.59614 | 1.5E-02 | KLF1 |
| 0.00478 | -0.59366 | 3.7E-02 | RARG |
| 0.00031 | -0.59302 | 4.9E-03 | SF1 |
| 0.00044 | -0.59291 | 6.4E-03 | MSN |
| 0.00461 | -0.59159 | 3.6E-02 | VRK3 |
| 0.00048 | -0.59093 | 6.8E-03 | PKM |
| 0.00021 | -0.58838 | 3.6E-03 | WIPF2 |
| 0.00260 | -0.58548 | 2.4E-02 | DGKQ |
| 0.00018 | -0.58541 | 3.2E-03 | SASH3 |
| 0.00031 | -0.58477 | 4.9E-03 | TAF10 |
| 0.00025 | -0.58396 | 4.2E-03 | ZYX |
| 0.00209 | -0.58339 | 2.1E-02 | SNORA5B |
| 0.00040 | -0.58295 | 5.9E-03 | ANKHD1 |
| 0.00040 | -0.58097 | 5.9E-03 | IMPDH1 |
| 0.00615 | -0.58081 | 4.4E-02 | DUSP26 |
| 0.00666 | -0.58052 | 4.7E-02 | PMEL |
| 0.00428 | -0.57947 | 3.4E-02 | COPG1 |
| 0.00096 | -0.57796 | 1.2E-02 | PLOD1 |
| 0.00491 | -0.57652 | 3.8E-02 | CCR7 |
| 0.00016 | -0.57633 | 2.9E-03 | EIF3C |
| 0.00014 | -0.57578 | 2.6E-03 | ARAF |
| 0.00226 | -0.57546 | 2.2E-02 | MKRN4P |
| 0.00383 | -0.57420 | 3.2E-02 | TFF1 |
| 0.00392 | -0.57346 | 3.2E-02 | MUC8 |
| 0.00097 | -0.57336 | 1.2E-02 | HILPDA |
| 0.00025 | -0.57264 | 4.1E-03 | NDRG1 |
| 0.00091 | -0.57037 | 1.1E-02 | ATP6V0D1 |
| 0.00459 | -0.57011 | 3.6E-02 | ARHGEF15 |
| 0.00013 | -0.56966 | 2.5E-03 | CFL1 |
| 0.00345 | -0.56851 | 3.0E-02 | CDC42EP4 |
| 0.00357 | -0.56840 | 3.0E-02 | NMB |
| 0.00403 | -0.56664 | 3.3E-02 | CDK5RAP3 |
| 0.00405 | -0.56601 | 3.3E-02 | FASN |
| 0.00015 | -0.56557 | 2.9E-03 | HLA-J |
| 0.00317 | -0.56498 | 2.8E-02 | FXYD3 |
| 0.00108 | -0.56412 | 1.3E-02 | RHOF |
| 0.00616 | -0.56369 | 4.4E-02 | MEPCE |
| 0.00331 | -0.56335 | 2.9E-02 | HBE1 |
| 0.00060 | -0.56250 | 8.1E-03 | SEC16A |
| 0.00073 | -0.56190 | 9.3E-03 | PUF60 |
| 0.00424 | -0.56093 | 3.4E-02 | ACRV1 |
| 0.00224 | -0.56031 | 2.2E-02 | HGH1 |
| 0.00059 | -0.56000 | 8.0E-03 | PIM1 |
| 0.00419 | -0.55984 | 3.4E-02 | RBM6 |
| 0.00492 | -0.55913 | 3.8E-02 | SPTBN2 |
| 0.00193 | -0.55894 | 2.0E-02 | GNB3 |
| 0.00201 | -0.55884 | 2.0E-02 | GSN |
| 0.00205 | -0.55883 | 2.0E-02 | IL2RG |
| 0.00068 | -0.55718 | 8.9E-03 | ADD1 |
| 0.00502 | -0.55687 | 3.8E-02 | HAS1 |
| 0.00204 | -0.55151 | 2.0E-02 | PVRL2 |
| 0.00029 | -0.55131 | 4.6E-03 | HSPA1A |
| 0.00415 | -0.55129 | 3.4E-02 | KIF3C |
| 0.00725 | -0.55101 | 5.0E-02 | BRD7P3 |
| 0.00090 | -0.54965 | 1.1E-02 | NDUFB11 |
| 0.00028 | -0.54933 | 4.5E-03 | PABPN1 |
| 0.00014 | -0.54919 | 2.7E-03 | C9orf16 |
| 0.00089 | -0.54841 | 1.1E-02 | SELPLG |
| 0.00031 | -0.54803 | 4.8E-03 | CSNK2B |
| 0.00101 | -0.54781 | 1.2E-02 | EIF5A |
| 0.00379 | -0.54762 | 3.2E-02 | NAT8 |
| 0.00017 | -0.54738 | 3.0E-03 | MKNK2 |
| 0.00056 | -0.54672 | 7.7E-03 | GRN |
| 0.00102 | -0.54663 | 1.2E-02 | BRE |
| 0.00429 | -0.54581 | 3.4E-02 | MBP |
| 0.00667 | -0.54574 | 4.7E-02 | SCNN1B |
| 0.00078 | -0.54504 | 9.8E-03 | SPG21 |
| 0.00297 | -0.54435 | 2.7E-02 | NCKAP1L |
| 0.00342 | -0.54423 | 2.9E-02 | ABI2 |
| 0.00227 | -0.54337 | 2.2E-02 | CLN3 |
| 0.00108 | -0.54317 | 1.3E-02 | CD3E |
| 0.00019 | -0.54248 | 3.3E-03 | CSNK1G2 |
| 0.00130 | -0.54130 | 1.5E-02 | FAM102A |
| 0.00299 | -0.54051 | 2.7E-02 | CTTN |
| 0.00577 | -0.53986 | 4.2E-02 | PPP3R1 |
| 0.00456 | -0.53852 | 3.6E-02 | MED25 |
| 0.00419 | -0.53610 | 3.4E-02 | TPP1 |
| 0.00317 | -0.53555 | 2.8E-02 | DDX24 |
| 0.00172 | -0.53497 | 1.8E-02 | TBC1D9B |
| 0.00272 | -0.53175 | 2.5E-02 | ABLIM1 |
| 0.00044 | -0.53018 | 6.4E-03 | KLF2 |
| 0.00025 | -0.53010 | 4.1E-03 | POM121 |
| 0.00180 | -0.52864 | 1.9E-02 | SLC5A5 |
| 0.00552 | -0.52693 | 4.1E-02 | CRYBB3 |
| 0.00427 | -0.52575 | 3.4E-02 | EVL |
| 0.00108 | -0.52413 | 1.3E-02 | ADRM1 |
| 0.00324 | -0.52359 | 2.8E-02 | FSHR |
| 0.00596 | -0.52304 | 4.3E-02 | SND1 |
| 0.00363 | -0.52289 | 3.1E-02 | E2F1 |
| 0.00373 | -0.52243 | 3.1E-02 | KDM4A |
| 0.00266 | -0.52233 | 2.5E-02 | LRP10 |
| 0.00487 | -0.52193 | 3.8E-02 | DRD2 |
| 0.00165 | -0.51655 | 1.7E-02 | TNPO1 |
| 0.00632 | -0.51655 | 4.5E-02 | ATG2A |
| 0.00202 | -0.51597 | 2.0E-02 | ADAM15 |
| 0.00270 | -0.51581 | 2.5E-02 | GPI |
| 0.00175 | -0.51562 | 1.8E-02 | ADAR |
| 0.00141 | -0.51505 | 1.5E-02 | MAPKAPK2 |
| 0.00466 | -0.51342 | 3.6E-02 | PDE4A |
| 0.00168 | -0.51319 | 1.8E-02 | TNIP1 |
| 0.00217 | -0.51280 | 2.1E-02 | FIS1 |
| 0.00456 | -0.51034 | 3.6E-02 | SLC12A7 |
| 0.00180 | -0.51001 | 1.9E-02 | CUTA |
| 0.00388 | -0.50843 | 3.2E-02 | MICAL1 |
| 0.00575 | -0.50614 | 4.2E-02 | PIGL |
| 0.00283 | -0.50590 | 2.6E-02 | GGT1 |
| 0.00272 | -0.50557 | 2.5E-02 | IFT122 |
| 0.00479 | -0.50526 | 3.7E-02 | HTR3A |
| 0.00096 | -0.50466 | 1.2E-02 | BCL2L2 |
| 0.00035 | -0.50343 | 5.4E-03 | PPP1R11 |
| 0.00335 | -0.50202 | 2.9E-02 | MED1 |
| 0.00356 | -0.50202 | 3.0E-02 | SSBP3 |
| 0.00271 | -0.49870 | 2.5E-02 | BCORL1 |
| 0.00216 | -0.49818 | 2.1E-02 | SLC12A9 |
| 0.00309 | -0.49726 | 2.7E-02 | FNBP1 |
| 0.00547 | -0.49700 | 4.1E-02 | PINK1 |
| 0.00102 | -0.49387 | 1.2E-02 | MAGED1 |
| 0.00653 | -0.49240 | 4.6E-02 | GLTSCR2 |
| 0.00343 | -0.48989 | 2.9E-02 | HLA-DQB1 |
| 0.00238 | -0.48972 | 2.3E-02 | MFSD7 |
| 0.00509 | -0.48838 | 3.9E-02 | ADAMTS12 |
| 0.00468 | -0.48833 | 3.6E-02 | GRAMD1B |
| 0.00115 | -0.48833 | 1.3E-02 | GNB1 |
| 0.00671 | -0.48783 | 4.7E-02 | ANPEP |
| 0.00368 | -0.48718 | 3.1E-02 | TTLL12 |
| 0.00331 | -0.48552 | 2.9E-02 | CAMK1 |
| 0.00577 | -0.48495 | 4.2E-02 | PFDN4 |
| 0.00397 | -0.48482 | 3.3E-02 | TRBC1 |
| 0.00428 | -0.48404 | 3.4E-02 | PRH1 |
| 0.00112 | -0.48351 | 1.3E-02 | ANXA6 |
| 0.00612 | -0.48001 | 4.4E-02 | CKAP4 |
| 0.00383 | -0.47928 | 3.2E-02 | ITGB2 |
| 0.00170 | -0.47893 | 1.8E-02 | IL10RA |
| 0.00071 | -0.47854 | 9.2E-03 | RPS6KA1 |
| 0.00699 | -0.47728 | 4.8E-02 | CDC34 |
| 0.00375 | -0.47383 | 3.1E-02 | POP7 |
| 0.00571 | -0.47277 | 4.2E-02 | KIF2C |
| 0.00327 | -0.47193 | 2.9E-02 | MGLL |
| 0.00281 | -0.46981 | 2.6E-02 | MAP3K3 |
| 0.00225 | -0.46904 | 2.2E-02 | USP20 |
| 0.00150 | -0.46800 | 1.6E-02 | FKBP1A |
| 0.00353 | -0.46783 | 3.0E-02 | NOTCH1 |
| 0.00517 | -0.46732 | 3.9E-02 | GNAS |
| 0.00086 | -0.46727 | 1.1E-02 | ICAM2 |
| 0.00366 | -0.46447 | 3.1E-02 | CSF1R |
| 0.00241 | -0.46398 | 2.3E-02 | GID8 |
| 0.00571 | -0.46332 | 4.2E-02 | LOC100506504 |
| 0.00230 | -0.46228 | 2.2E-02 | AFTPH |
| 0.00673 | -0.46074 | 4.7E-02 | VCP |
| 0.00532 | -0.45908 | 4.0E-02 | SLN |
| 0.00475 | -0.45874 | 3.7E-02 | NUDC |
| 0.00135 | -0.45868 | 1.5E-02 | MEN1 |
| 0.00350 | -0.45590 | 3.0E-02 | INTS3 |
| 0.00478 | -0.45321 | 3.7E-02 | TUBA4A |
| 0.00369 | -0.45286 | 3.1E-02 | DUS1L |
| 0.00386 | -0.45193 | 3.2E-02 | TRIP10 |
| 0.00204 | -0.45133 | 2.0E-02 | GABARAP |
| 0.00160 | -0.45025 | 1.7E-02 | RBM42 |
| 0.00215 | -0.44966 | 2.1E-02 | SIRPG |
| 0.00674 | -0.44798 | 4.7E-02 | TMC6 |
| 0.00272 | -0.44631 | 2.5E-02 | ZBTB16 |
| 0.00132 | -0.44392 | 1.5E-02 | EEF2 |
| 0.00325 | -0.44332 | 2.8E-02 | SARS |
| 0.00242 | -0.44206 | 2.3E-02 | SQSTM1 |
| 0.00555 | -0.44156 | 4.1E-02 | WNT5B |
| 0.00287 | -0.44133 | 2.6E-02 | NCOR2 |
| 0.00154 | -0.43895 | 1.6E-02 | UBXN1 |
| 0.00222 | -0.43670 | 2.2E-02 | HSP90AB1 |
| 0.00492 | -0.43634 | 3.8E-02 | SLC25A6 |
| 0.00359 | -0.43479 | 3.0E-02 | PNPLA6 |
| 0.00576 | -0.43471 | 4.2E-02 | OR7E37P |
| 0.00143 | -0.43465 | 1.5E-02 | MCM3 |
| 0.00686 | -0.42847 | 4.8E-02 | FAM160B2 |
| 0.00106 | -0.42829 | 1.2E-02 | IL16 |
| 0.00424 | -0.42690 | 3.4E-02 | IMP3 |
| 0.00535 | -0.42433 | 4.0E-02 | ZNF721 |
| 0.00367 | -0.42266 | 3.1E-02 | NISCH |
| 0.00213 | -0.42102 | 2.1E-02 | KLF3 |
| 0.00249 | -0.41513 | 2.3E-02 | HCLS1 |
| 0.00143 | -0.41505 | 1.5E-02 | NSFL1C |
| 0.00502 | -0.41369 | 3.8E-02 | HYOU1 |
| 0.00248 | -0.41312 | 2.3E-02 | DYNLRB1 |
| 0.00689 | -0.41297 | 4.8E-02 | COL9A2 |
| 0.00218 | -0.41286 | 2.1E-02 | ARFGAP2 |
| 0.00573 | -0.40919 | 4.2E-02 | BCS1L |
| 0.00249 | -0.40799 | 2.3E-02 | LSS |
| 0.00338 | -0.40545 | 2.9E-02 | EIF4B |
| 0.00506 | -0.40415 | 3.9E-02 | CD99 |
| 0.00279 | -0.39946 | 2.5E-02 | COA3 |
| 0.00472 | -0.39944 | 3.7E-02 | FICD |
| 0.00374 | -0.39928 | 3.1E-02 | AP2A2 |
| 0.00606 | -0.39695 | 4.4E-02 | ARHGEF1 |
| 0.00372 | -0.39618 | 3.1E-02 | ASXL2 |
| 0.00327 | -0.39106 | 2.9E-02 | ARHGAP1 |
| 0.00716 | -0.37901 | 4.9E-02 | GALT |
| 0.00435 | -0.37606 | 3.5E-02 | EIF6 |
| 0.00225 | -0.37413 | 2.2E-02 | GCC1 |
| 0.00629 | 0.33803 | 4.5E-02 | VAPA |
| 0.00694 | 0.35321 | 4.8E-02 | CYTIP |
| 0.00471 | 0.35397 | 3.7E-02 | HMGN2 |
| 0.00462 | 0.38368 | 3.6E-02 | TXNL1 |
| 0.00442 | 0.38986 | 3.5E-02 | CTSO |
| 0.00648 | 0.39472 | 4.6E-02 | MPC1 |
| 0.00610 | 0.40181 | 4.4E-02 | DAZAP2 |
| 0.00528 | 0.40186 | 4.0E-02 | MOB4 |
| 0.00528 | 0.40255 | 4.0E-02 | HLA-DRA |
| 0.00679 | 0.40658 | 4.7E-02 | METTL7A |
| 0.00557 | 0.40982 | 4.1E-02 | PPP2R2A |
| 0.00111 | 0.41740 | 1.3E-02 | SH3GLB1 |
| 0.00225 | 0.41975 | 2.2E-02 | OGT |
| 0.00617 | 0.42206 | 4.4E-02 | UQCRC2 |
| 0.00323 | 0.43301 | 2.8E-02 | LOC101928676 |
| 0.00407 | 0.43386 | 3.3E-02 | CANX |
| 0.00609 | 0.43388 | 4.4E-02 | SEPHS1 |
| 0.00701 | 0.43445 | 4.8E-02 | NUP37 |
| 0.00572 | 0.43768 | 4.2E-02 | RALGAPB |
| 0.00367 | 0.43883 | 3.1E-02 | ZCCHC11 |
| 0.00358 | 0.44531 | 3.0E-02 | CASP4 |
| 0.00317 | 0.44796 | 2.8E-02 | GDI2 |
| 0.00220 | 0.45061 | 2.1E-02 | KRT10 |
| 0.00637 | 0.45062 | 4.6E-02 | CCNH |
| 0.00250 | 0.45299 | 2.3E-02 | PTMA |
| 0.00156 | 0.45412 | 1.7E-02 | GGNBP2 |
| 0.00440 | 0.45526 | 3.5E-02 | NFIL3 |
| 0.00619 | 0.45562 | 4.5E-02 | PRDX4 |
| 0.00505 | 0.45592 | 3.9E-02 | HNRNPA0 |
| 0.00084 | 0.45944 | 1.1E-02 | GTF2E1 |
| 0.00225 | 0.45979 | 2.2E-02 | B2M |
| 0.00703 | 0.46165 | 4.9E-02 | SRSF3 |
| 0.00483 | 0.46456 | 3.7E-02 | CPVL |
| 0.00145 | 0.46606 | 1.6E-02 | ALG5 |
| 0.00268 | 0.46616 | 2.5E-02 | CCT4 |
| 0.00382 | 0.46694 | 3.2E-02 | PHIP |
| 0.00377 | 0.46725 | 3.1E-02 | RPL6 |
| 0.00130 | 0.46896 | 1.5E-02 | CLIC1 |
| 0.00714 | 0.46907 | 4.9E-02 | VWA8 |
| 0.00641 | 0.47019 | 4.6E-02 | AMIGO2 |
| 0.00627 | 0.47032 | 4.5E-02 | S100PBP |
| 0.00494 | 0.47063 | 3.8E-02 | ANP32A |
| 0.00652 | 0.47187 | 4.6E-02 | DNAJC13 |
| 0.00616 | 0.47211 | 4.4E-02 | CAPRIN2 |
| 0.00140 | 0.47245 | 1.5E-02 | ADPGK |
| 0.00252 | 0.47298 | 2.4E-02 | RBM5 |
| 0.00130 | 0.47476 | 1.5E-02 | CMAHP |
| 0.00282 | 0.47821 | 2.6E-02 | DHRS7 |
| 0.00158 | 0.47906 | 1.7E-02 | CDK14 |
| 0.00141 | 0.48009 | 1.5E-02 | CBX1 |
| 0.00229 | 0.48074 | 2.2E-02 | ACVR1 |
| 0.00221 | 0.48342 | 2.1E-02 | TAF1D |
| 0.00641 | 0.48593 | 4.6E-02 | RHOQ |
| 0.00107 | 0.48666 | 1.2E-02 | LDHA |
| 0.00397 | 0.48725 | 3.3E-02 | TGS1 |
| 0.00059 | 0.48878 | 8.0E-03 | TMEM41B |
| 0.00301 | 0.49196 | 2.7E-02 | ANAPC13 |
| 0.00301 | 0.49201 | 2.7E-02 | TTC19 |
| 0.00239 | 0.49319 | 2.3E-02 | APAF1 |
| 0.00602 | 0.49635 | 4.4E-02 | HHEX |
| 0.00550 | 0.49689 | 4.1E-02 | SPG11 |
| 0.00238 | 0.49934 | 2.3E-02 | RPL4 |
| 0.00047 | 0.50498 | 6.7E-03 | ATP6V0E1 |
| 0.00045 | 0.50612 | 6.4E-03 | PTPRE |
| 0.00237 | 0.50738 | 2.3E-02 | CORO1C |
| 0.00418 | 0.50851 | 3.4E-02 | MNDA |
| 0.00301 | 0.51011 | 2.7E-02 | RPLP0 |
| 0.00406 | 0.51052 | 3.3E-02 | OPA1 |
| 0.00203 | 0.51140 | 2.0E-02 | DYNLT1 |
| 0.00663 | 0.51298 | 4.7E-02 | PIP5K1B |
| 0.00065 | 0.51304 | 8.6E-03 | ARMC1 |
| 0.00644 | 0.51311 | 4.6E-02 | LMO2 |
| 0.00426 | 0.51417 | 3.4E-02 | RGPD3 |
| 0.00639 | 0.51433 | 4.6E-02 | SECISBP2 |
| 0.00068 | 0.51520 | 8.9E-03 | CD53 |
| 0.00607 | 0.51795 | 4.4E-02 | SMARCE1 |
| 0.00663 | 0.51884 | 4.7E-02 | SERPINB1 |
| 0.00486 | 0.51961 | 3.8E-02 | EIF3M |
| 0.00090 | 0.52024 | 1.1E-02 | MYL12B |
| 0.00374 | 0.52294 | 3.1E-02 | SKP1 |
| 0.00482 | 0.52298 | 3.7E-02 | KHDRBS1 |
| 0.00288 | 0.52496 | 2.6E-02 | ARPC3 |
| 0.00091 | 0.52585 | 1.1E-02 | CCDC47 |
| 0.00510 | 0.52650 | 3.9E-02 | DYNC1I2 |
| 0.00615 | 0.52694 | 4.4E-02 | TXNDC15 |
| 0.00124 | 0.52791 | 1.4E-02 | GNL3 |
| 0.00304 | 0.52919 | 2.7E-02 | STOM |
| 0.00119 | 0.53076 | 1.4E-02 | MS4A6A |
| 0.00251 | 0.53254 | 2.4E-02 | MPC2 |
| 0.00492 | 0.53387 | 3.8E-02 | NAT1 |
| 0.00517 | 0.53449 | 3.9E-02 | MACF1 |
| 0.00642 | 0.53702 | 4.6E-02 | COQ10B |
| 0.00397 | 0.53705 | 3.3E-02 | ME2 |
| 0.00112 | 0.53808 | 1.3E-02 | HMGN1 |
| 0.00062 | 0.53931 | 8.3E-03 | TAX1BP1 |
| 0.00077 | 0.54269 | 9.8E-03 | USP3 |
| 0.00065 | 0.54500 | 8.6E-03 | SEC11A |
| 0.00627 | 0.54581 | 4.5E-02 | MORF4L1 |
| 0.00212 | 0.54641 | 2.1E-02 | GNPTAB |
| 0.00046 | 0.54684 | 6.6E-03 | CCR2 |
| 0.00588 | 0.54743 | 4.3E-02 | UTP6 |
| 0.00443 | 0.54786 | 3.5E-02 | FBXW2 |
| 0.00664 | 0.54836 | 4.7E-02 | CAPN2 |
| 0.00013 | 0.54972 | 2.5E-03 | WDR26 |
| 0.00030 | 0.55013 | 4.7E-03 | SAR1A |
| 0.00384 | 0.55047 | 3.2E-02 | RAB22A |
| 0.00687 | 0.55204 | 4.8E-02 | TFRC |
| 0.00531 | 0.55208 | 4.0E-02 | FRMD4B |
| 0.00392 | 0.55220 | 3.2E-02 | UBQLN2 |
| 0.00063 | 0.55348 | 8.4E-03 | PTPN4 |
| 0.00275 | 0.55687 | 2.5E-02 | RPL5 |
| 0.00353 | 0.56015 | 3.0E-02 | YTHDF2 |
| 0.00411 | 0.56260 | 3.3E-02 | CDV3 |
| 0.00291 | 0.56306 | 2.6E-02 | AKAP2 |
| 0.00651 | 0.56679 | 4.6E-02 | SLC2A14 |
| 0.00102 | 0.56774 | 1.2E-02 | PSMD12 |
| 0.00360 | 0.56800 | 3.0E-02 | ZCCHC8 |
| 0.00085 | 0.57073 | 1.1E-02 | DSTN |
| 0.00018 | 0.57346 | 3.2E-03 | YTHDC1 |
| 0.00663 | 0.57495 | 4.7E-02 | MANSC1 |
| 0.00415 | 0.57509 | 3.4E-02 | FAM136A |
| 0.00520 | 0.57590 | 3.9E-02 | FAM153A |
| 0.00015 | 0.57909 | 2.8E-03 | HEXB |
| 0.00035 | 0.57948 | 5.3E-03 | ERLIN1 |
| 0.00521 | 0.58053 | 3.9E-02 | TRIB2 |
| 0.00107 | 0.58099 | 1.2E-02 | UBE2E3 |
| 0.00628 | 0.58163 | 4.5E-02 | CHCHD2 |
| 0.00172 | 0.58221 | 1.8E-02 | G3BP1 |
| 0.00043 | 0.58321 | 6.2E-03 | MOSPD1 |
| 0.00248 | 0.58514 | 2.3E-02 | ARFGAP3 |
| 0.00109 | 0.58613 | 1.3E-02 | ZMYND11 |
| 0.00003 | 0.58633 | 8.1E-04 | MAT2B |
| 0.00660 | 0.58643 | 4.7E-02 | TXN |
| 0.00377 | 0.58742 | 3.1E-02 | SNX13 |
| 0.00015 | 0.58779 | 2.8E-03 | BTN2A1 |
| 0.00680 | 0.58833 | 4.7E-02 | LARP7 |
| 0.00079 | 0.58903 | 1.0E-02 | SH3BGRL |
| 0.00007 | 0.58916 | 1.6E-03 | EIF4G2 |
| 0.00569 | 0.59012 | 4.2E-02 | CEPT1 |
| 0.00469 | 0.59069 | 3.7E-02 | EIF2S1 |
| 0.00471 | 0.59112 | 3.7E-02 | HSPE1 |
| 0.00634 | 0.59134 | 4.5E-02 | HMG20A |
| 0.00123 | 0.59177 | 1.4E-02 | UBE2K |
| 0.00162 | 0.59194 | 1.7E-02 | WTAP |
| 0.00040 | 0.59222 | 5.9E-03 | KDELR2 |
| 0.00039 | 0.59228 | 5.8E-03 | CD48 |
| 0.00241 | 0.59283 | 2.3E-02 | MKKS |
| 0.00039 | 0.59331 | 5.8E-03 | ALG11 |
| 0.00082 | 0.59426 | 1.0E-02 | NBPF10 |
| 0.00109 | 0.59426 | 1.3E-02 | NPM1 |
| 0.00012 | 0.59493 | 2.4E-03 | TFB2M |
| 0.00084 | 0.59584 | 1.1E-02 | POLE3 |
| 0.00014 | 0.60098 | 2.6E-03 | MSL3 |
| 0.00586 | 0.60260 | 4.3E-02 | PPT1 |
| 0.00263 | 0.60338 | 2.4E-02 | OLA1 |
| 0.00205 | 0.60382 | 2.0E-02 | SGPP1 |
| 0.00218 | 0.60448 | 2.1E-02 | SESN1 |
| 0.00480 | 0.60467 | 3.7E-02 | LRPPRC |
| 0.00052 | 0.60566 | 7.2E-03 | TLR4 |
| 0.00017 | 0.60569 | 3.0E-03 | KDM7A |
| 0.00118 | 0.60599 | 1.3E-02 | RPL31 |
| 0.00074 | 0.61089 | 9.5E-03 | SLK |
| 0.00028 | 0.61189 | 4.5E-03 | CD47 |
| 0.00526 | 0.61268 | 4.0E-02 | HSPBAP1 |
| 0.00086 | 0.61282 | 1.1E-02 | TXNRD1 |
| 0.00027 | 0.61306 | 4.3E-03 | TMEM2 |
| 0.00366 | 0.61312 | 3.1E-02 | NEDD9 |
| 0.00005 | 0.61426 | 1.2E-03 | IDH1 |
| 0.00070 | 0.61745 | 9.1E-03 | ARHGEF3 |
| 0.00605 | 0.61943 | 4.4E-02 | SS18L2 |
| 0.00019 | 0.62214 | 3.3E-03 | LIMA1 |
| 0.00014 | 0.62277 | 2.7E-03 | PURA |
| 0.00034 | 0.62400 | 5.2E-03 | NDUFB5 |
| 0.00242 | 0.62434 | 2.3E-02 | PNRC1 |
| 0.00235 | 0.62475 | 2.2E-02 | HNRNPDL |
| 0.00192 | 0.62504 | 1.9E-02 | COPS5 |
| 0.00092 | 0.62538 | 1.1E-02 | PPP2R5C |
| 0.00574 | 0.62609 | 4.2E-02 | GTF3A |
| 0.00058 | 0.62626 | 7.9E-03 | GYG1 |
| 0.00055 | 0.62773 | 7.6E-03 | GLUD1 |
| 0.00081 | 0.62774 | 1.0E-02 | GJA9-MYCBP |
| 0.00003 | 0.62811 | 7.7E-04 | CNOT2 |
| 0.00232 | 0.62858 | 2.2E-02 | FRY |
| 0.00026 | 0.62912 | 4.2E-03 | HMGN4 |
| 0.00007 | 0.63002 | 1.5E-03 | TM9SF2 |
| 0.00033 | 0.63054 | 5.0E-03 | SDCBP |
| 0.00657 | 0.63079 | 4.6E-02 | FBXW11 |
| 0.00052 | 0.63323 | 7.3E-03 | SCAF8 |
| 0.00230 | 0.63516 | 2.2E-02 | MRS2 |
| 0.00144 | 0.63923 | 1.6E-02 | SRP14 |
| 0.00019 | 0.64121 | 3.4E-03 | CCBL2 |
| 0.00398 | 0.64152 | 3.3E-02 | GCOM1 |
| 0.00022 | 0.64290 | 3.8E-03 | HPRT1 |
| 0.00140 | 0.64374 | 1.5E-02 | VPS13B |
| 0.00110 | 0.64660 | 1.3E-02 | SLBP |
| 0.00098 | 0.64939 | 1.2E-02 | COPS3 |
| 0.00714 | 0.64983 | 4.9E-02 | ISCA1 |
| 0.00191 | 0.65049 | 1.9E-02 | TIPARP |
| 0.00664 | 0.65061 | 4.7E-02 | FPR2 |
| 0.00012 | 0.65556 | 2.3E-03 | PPP2CA |
| 0.00024 | 0.65706 | 3.9E-03 | HNRNPK |
| 0.00014 | 0.65913 | 2.6E-03 | LAPTM4A |
| 0.00446 | 0.65951 | 3.5E-02 | EGLN1 |
| 0.00002 | 0.65993 | 6.5E-04 | EVI2B |
| 0.00002 | 0.66134 | 5.5E-04 | CASP1 |
| 0.00115 | 0.66249 | 1.3E-02 | ISOC1 |
| 0.00029 | 0.66401 | 4.6E-03 | HSPH1 |
| 0.00295 | 0.66416 | 2.6E-02 | SLC20A1 |
| 0.00002 | 0.66425 | 5.6E-04 | DENR |
| 0.00049 | 0.66821 | 6.9E-03 | ERH |
| 0.00022 | 0.67012 | 3.8E-03 | ADSS |
| 0.00604 | 0.67020 | 4.4E-02 | PLCL2 |
| 0.00358 | 0.67143 | 3.0E-02 | PSMD7 |
| 0.00027 | 0.67210 | 4.4E-03 | FRYL |
| 0.00506 | 0.67243 | 3.9E-02 | CLNS1A |
| 0.00099 | 0.67266 | 1.2E-02 | SH3YL1 |
| 0.00020 | 0.67530 | 3.5E-03 | LRMP |
| 0.00458 | 0.67648 | 3.6E-02 | FBXO34 |
| 0.00012 | 0.67834 | 2.3E-03 | HNRNPA1 |
| 0.00054 | 0.67879 | 7.5E-03 | RGS2 |
| 0.00137 | 0.67940 | 1.5E-02 | FNBP4 |
| 0.00056 | 0.68096 | 7.7E-03 | CDC23 |
| 0.00016 | 0.68103 | 3.0E-03 | COPB1 |
| 0.00210 | 0.68157 | 2.1E-02 | HSD17B4 |
| 0.00026 | 0.68178 | 4.3E-03 | ANXA7 |
| 0.00019 | 0.68349 | 3.4E-03 | ZDHHC6 |
| 0.00333 | 0.68489 | 2.9E-02 | BAZ1A |
| 0.00044 | 0.68496 | 6.3E-03 | DCUN1D1 |
| 0.00016 | 0.68651 | 3.0E-03 | AFF1 |
| 0.00506 | 0.68857 | 3.9E-02 | DDAH2 |
| 0.00029 | 0.69084 | 4.6E-03 | ACTR10 |
| 0.00147 | 0.69221 | 1.6E-02 | BANK1 |
| 0.00021 | 0.69305 | 3.6E-03 | ETFA |
| 0.00098 | 0.69515 | 1.2E-02 | IFI16 |
| 0.00003 | 0.69654 | 8.0E-04 | CHMP3 |
| 0.00004 | 0.69673 | 9.8E-04 | ZFC3H1 |
| 0.00132 | 0.69814 | 1.5E-02 | SMCO4 |
| 0.00051 | 0.69905 | 7.2E-03 | TM9SF3 |
| 0.00444 | 0.69937 | 3.5E-02 | EML4 |
| 0.00007 | 0.70276 | 1.6E-03 | ARL5A |
| 0.00399 | 0.70278 | 3.3E-02 | CDC40 |
| 0.00475 | 0.70370 | 3.7E-02 | STK39 |
| 0.00022 | 0.70413 | 3.7E-03 | GOLPH3 |
| 0.00000 | 0.70578 | 1.7E-04 | UGP2 |
| 0.00044 | 0.70680 | 6.4E-03 | DAPP1 |
| 0.00001 | 0.70708 | 3.4E-04 | USP9X |
| 0.00053 | 0.70739 | 7.4E-03 | UBE2B |
| 0.00005 | 0.70841 | 1.1E-03 | HNRNPC |
| 0.00635 | 0.70859 | 4.5E-02 | FCGR1B |
| 0.00046 | 0.70951 | 6.6E-03 | ARID5B |
| 0.00094 | 0.70992 | 1.1E-02 | CAT |
| 0.00108 | 0.71044 | 1.3E-02 | GPBP1L1 |
| 0.00001 | 0.71052 | 4.4E-04 | WAC |
| 0.00056 | 0.71280 | 7.7E-03 | UBE2D2 |
| 0.00462 | 0.71779 | 3.6E-02 | PRKX |
| 0.00217 | 0.72035 | 2.1E-02 | SERINC5 |
| 0.00115 | 0.72069 | 1.3E-02 | FCGR2C |
| 0.00667 | 0.72382 | 4.7E-02 | REEP5 |
| 0.00155 | 0.72478 | 1.7E-02 | SUMO1 |
| 0.00634 | 0.72508 | 4.5E-02 | TPP2 |
| 0.00007 | 0.72528 | 1.5E-03 | YWHAQ |
| 0.00143 | 0.72622 | 1.5E-02 | CCDC90B |
| 0.00001 | 0.72642 | 3.3E-04 | ORC4 |
| 0.00034 | 0.72655 | 5.2E-03 | NCOA4 |
| 0.00390 | 0.73050 | 3.2E-02 | SMPDL3A |
| 0.00310 | 0.73137 | 2.7E-02 | EAPP |
| 0.00003 | 0.73231 | 7.6E-04 | KIAA0907 |
| 0.00025 | 0.73238 | 4.2E-03 | GLRX |
| 0.00303 | 0.73360 | 2.7E-02 | SNRPA1 |
| 0.00038 | 0.73443 | 5.6E-03 | CCDC109B |
| 0.00145 | 0.73500 | 1.6E-02 | CAND1 |
| 0.00245 | 0.73531 | 2.3E-02 | RNF13 |
| 0.00078 | 0.73811 | 9.9E-03 | DHX40 |
| 0.00564 | 0.73833 | 4.2E-02 | AXIN1 |
| 0.00001 | 0.73894 | 4.5E-04 | ARPC5 |
| 0.00001 | 0.74020 | 2.1E-04 | UBR5 |
| 0.00177 | 0.74077 | 1.8E-02 | ACTL6A |
| 0.00138 | 0.74338 | 1.5E-02 | MIS12 |
| 0.00190 | 0.74558 | 1.9E-02 | PRKAG1 |
| 0.00028 | 0.74596 | 4.5E-03 | ZNF146 |
| 0.00036 | 0.74671 | 5.5E-03 | GMFG |
| 0.00068 | 0.75456 | 8.9E-03 | MYL6 |
| 0.00004 | 0.75468 | 9.5E-04 | THOC7 |
| 0.00295 | 0.75480 | 2.6E-02 | CCDC59 |
| 0.00135 | 0.75827 | 1.5E-02 | MDH1 |
| 0.00025 | 0.75883 | 4.1E-03 | CTR9 |
| 0.00060 | 0.76099 | 8.1E-03 | CAAP1 |
| 0.00113 | 0.76319 | 1.3E-02 | TRIP12 |
| 0.00105 | 0.76328 | 1.2E-02 | PRKCB |
| 0.00078 | 0.76544 | 9.9E-03 | ELF2 |
| 0.00002 | 0.76587 | 6.5E-04 | YWHAZ |
| 0.00208 | 0.76590 | 2.1E-02 | SCARB2 |
| 0.00029 | 0.76650 | 4.7E-03 | ZC3H7A |
| 0.00333 | 0.76886 | 2.9E-02 | FAM63B |
| 0.00550 | 0.76984 | 4.1E-02 | ACAT1 |
| 0.00006 | 0.77118 | 1.5E-03 | MIR636 |
| 0.00036 | 0.77359 | 5.4E-03 | FAM60A |
| 0.00037 | 0.77811 | 5.6E-03 | EPM2AIP1 |
| 0.00009 | 0.77840 | 1.9E-03 | CD28 |
| 0.00349 | 0.77854 | 3.0E-02 | ITGA2 |
| 0.00006 | 0.77879 | 1.3E-03 | KIAA0232 |
| 0.00356 | 0.77953 | 3.0E-02 | FCER1A |
| 0.00001 | 0.78047 | 4.1E-04 | STRAP |
| 0.00004 | 0.78080 | 9.9E-04 | TIMM8B |
| 0.00030 | 0.78102 | 4.7E-03 | NFU1 |
| 0.00439 | 0.78128 | 3.5E-02 | TIA1 |
| 0.00015 | 0.78160 | 2.8E-03 | SPTSSA |
| 0.00046 | 0.78246 | 6.6E-03 | HOPX |
| 0.00395 | 0.78275 | 3.2E-02 | ANXA3 |
| 0.00019 | 0.78453 | 3.4E-03 | ZNF143 |
| 0.00005 | 0.78529 | 1.2E-03 | TANK |
| 0.00013 | 0.78548 | 2.5E-03 | RPL30 |
| 0.00013 | 0.78552 | 2.5E-03 | TOP2B |
| 0.00020 | 0.78602 | 3.4E-03 | DESI2 |
| 0.00001 | 0.78616 | 4.1E-04 | PARP8 |
| 0.00152 | 0.78738 | 1.6E-02 | RYK |
| 0.00003 | 0.78825 | 8.1E-04 | USP16 |
| 0.00000 | 0.78888 | 1.0E-04 | MRFAP1L1 |
| 0.00127 | 0.78918 | 1.4E-02 | CPNE3 |
| 0.00185 | 0.79124 | 1.9E-02 | RBX1 |
| 0.00258 | 0.79150 | 2.4E-02 | RNF7 |
| 0.00017 | 0.79203 | 3.0E-03 | STX3 |
| 0.00008 | 0.79219 | 1.7E-03 | YME1L1 |
| 0.00099 | 0.79428 | 1.2E-02 | PRKAR2B |
| 0.00021 | 0.79522 | 3.6E-03 | GLO1 |
| 0.00000 | 0.79633 | 1.4E-04 | UBE4A |
| 0.00001 | 0.80027 | 3.6E-04 | GHITM |
| 0.00008 | 0.80049 | 1.7E-03 | TRA2B |
| 0.00117 | 0.80159 | 1.3E-02 | TMPO |
| 0.00015 | 0.80174 | 2.8E-03 | HIST1H2AC |
| 0.00044 | 0.80312 | 6.4E-03 | SEL1L |
| 0.00179 | 0.80332 | 1.8E-02 | RPS21 |
| 0.00292 | 0.80362 | 2.6E-02 | LOC101928625 |
| 0.00106 | 0.80393 | 1.2E-02 | RNF219 |
| 0.00588 | 0.80560 | 4.3E-02 | PRKRA |
| 0.00264 | 0.80571 | 2.4E-02 | TLR6 |
| 0.00211 | 0.80912 | 2.1E-02 | ZNF281 |
| 0.00030 | 0.81180 | 4.8E-03 | HMGN3 |
| 0.00000 | 0.81309 | 1.1E-04 | PTPLB |
| 0.00004 | 0.81355 | 9.2E-04 | DNTTIP2 |
| 0.00004 | 0.81440 | 9.9E-04 | PIGB |
| 0.00670 | 0.81443 | 4.7E-02 | FOXA1 |
| 0.00005 | 0.81473 | 1.2E-03 | LYRM1 |
| 0.00026 | 0.81516 | 4.3E-03 | ATP5F1 |
| 0.00241 | 0.81579 | 2.3E-02 | PPP4R1 |
| 0.00048 | 0.81769 | 6.8E-03 | AZIN1 |
| 0.00038 | 0.81827 | 5.7E-03 | FKBP3 |
| 0.00001 | 0.81846 | 4.4E-04 | ITK |
| 0.00008 | 0.81852 | 1.8E-03 | GIMAP4 |
| 0.00059 | 0.82229 | 8.0E-03 | MGEA5 |
| 0.00045 | 0.82279 | 6.4E-03 | MFF |
| 0.00008 | 0.82449 | 1.7E-03 | LEPROT |
| 0.00080 | 0.82509 | 1.0E-02 | TBCC |
| 0.00383 | 0.82657 | 3.2E-02 | CHMP1B |
| 0.00001 | 0.82692 | 2.3E-04 | C5orf22 |
| 0.00102 | 0.82704 | 1.2E-02 | POLB |
| 0.00003 | 0.82720 | 7.6E-04 | CNIH1 |
| 0.00571 | 0.82735 | 4.2E-02 | RP3-334F4.1 |
| 0.00327 | 0.82788 | 2.9E-02 | CKAP2 |
| 0.00648 | 0.82823 | 4.6E-02 | FAM206A |
| 0.00441 | 0.82840 | 3.5E-02 | TOPBP1 |
| 0.00001 | 0.83031 | 2.5E-04 | DDX5 |
| 0.00001 | 0.83285 | 3.7E-04 | CYLD |
| 0.00313 | 0.83331 | 2.8E-02 | ACOT9 |
| 0.00064 | 0.83337 | 8.5E-03 | ASNSD1 |
| 0.00002 | 0.83394 | 5.5E-04 | USP8 |
| 0.00008 | 0.83396 | 1.8E-03 | LOC101928826 |
| 0.00006 | 0.83465 | 1.3E-03 | NUP50 |
| 0.00018 | 0.83527 | 3.2E-03 | AP5M1 |
| 0.00005 | 0.83562 | 1.1E-03 | ADCY7 |
| 0.00002 | 0.83570 | 5.9E-04 | PCF11 |
| 0.00011 | 0.83624 | 2.3E-03 | CSF2RB |
| 0.00001 | 0.83625 | 4.0E-04 | SNAP23 |
| 0.00347 | 0.83783 | 3.0E-02 | FCGR1A |
| 0.00015 | 0.83922 | 2.7E-03 | CSRNP2 |
| 0.00000 | 0.84052 | 1.5E-04 | SLC5A3 |
| 0.00000 | 0.84096 | 9.9E-05 | TMEM59 |
| 0.00506 | 0.84383 | 3.9E-02 | MIR6132 |
| 0.00133 | 0.84477 | 1.5E-02 | KPNA2 |
| 0.00000 | 0.84582 | 1.8E-04 | BZW1 |
| 0.00618 | 0.84702 | 4.5E-02 | HMGCR |
| 0.00006 | 0.84983 | 1.4E-03 | ZNF22 |
| 0.00003 | 0.85145 | 8.1E-04 | DNAJB9 |
| 0.00001 | 0.85284 | 4.0E-04 | ADNP |
| 0.00466 | 0.85314 | 3.6E-02 | IL15 |
| 0.00355 | 0.85363 | 3.0E-02 | ZNF654 |
| 0.00003 | 0.85623 | 8.5E-04 | KRAS |
| 0.00019 | 0.85755 | 3.4E-03 | ADM |
| 0.00011 | 0.85899 | 2.2E-03 | RAB6A |
| 0.00062 | 0.85950 | 8.2E-03 | EIF5 |
| 0.00343 | 0.86058 | 2.9E-02 | TIMM17A |
| 0.00001 | 0.86059 | 2.3E-04 | TMEM243 |
| 0.00001 | 0.86096 | 3.7E-04 | PRPS2 |
| 0.00418 | 0.86139 | 3.4E-02 | GPRASP1 |
| 0.00110 | 0.86315 | 1.3E-02 | AHCYL1 |
| 0.00013 | 0.86757 | 2.5E-03 | DUSP6 |
| 0.00000 | 0.86869 | 1.9E-04 | UBE2G1 |
| 0.00023 | 0.86871 | 3.9E-03 | HDAC2 |
| 0.00018 | 0.86920 | 3.3E-03 | STAM |
| 0.00002 | 0.86953 | 5.2E-04 | SOAT1 |
| 0.00024 | 0.87086 | 3.9E-03 | MOAP1 |
| 0.00035 | 0.87200 | 5.3E-03 | LOC101928061 |
| 0.00167 | 0.87211 | 1.8E-02 | SNIP1 |
| 0.00003 | 0.87325 | 8.0E-04 | RPF1 |
| 0.00002 | 0.87510 | 6.0E-04 | AP3S1 |
| 0.00003 | 0.87616 | 8.8E-04 | ATP6V1G1 |
| 0.00001 | 0.87710 | 2.1E-04 | GMCL1 |
| 0.00695 | 0.87743 | 4.8E-02 | ZC3H14 |
| 0.00009 | 0.87835 | 1.8E-03 | MIR21 |
| 0.00003 | 0.87918 | 8.9E-04 | METTL9 |
| 0.00001 | 0.87984 | 2.4E-04 | KLHDC2 |
| 0.00008 | 0.88010 | 1.7E-03 | NUP153 |
| 0.00648 | 0.88079 | 4.6E-02 | ZCCHC10 |
| 0.00084 | 0.88106 | 1.0E-02 | GOLGA4 |
| 0.00000 | 0.88529 | 1.9E-04 | NSMAF |
| 0.00228 | 0.88805 | 2.2E-02 | CLPX |
| 0.00720 | 0.88940 | 4.9E-02 | CHUK |
| 0.00002 | 0.88995 | 6.3E-04 | PDS5A |
| 0.00402 | 0.89065 | 3.3E-02 | ING1 |
| 0.00015 | 0.89077 | 2.7E-03 | RASGRP1 |
| 0.00109 | 0.89340 | 1.3E-02 | CASP5 |
| 0.00100 | 0.89440 | 1.2E-02 | CNTRL |
| 0.00635 | 0.89713 | 4.5E-02 | RAP1GDS1 |
| 0.00006 | 0.89726 | 1.4E-03 | RP6-11O7.2 |
| 0.00538 | 0.89856 | 4.0E-02 | DDX46 |
| 0.00007 | 0.89893 | 1.5E-03 | LCP2 |
| 0.00000 | 0.90181 | 1.2E-04 | EIF3A |
| 0.00313 | 0.90200 | 2.8E-02 | MAP3K8 |
| 0.00004 | 0.90229 | 1.1E-03 | MFSD1 |
| 0.00002 | 0.90238 | 5.3E-04 | TCEA1 |
| 0.00637 | 0.90403 | 4.6E-02 | NUP107 |
| 0.00099 | 0.90461 | 1.2E-02 | CDK7 |
| 0.00040 | 0.90461 | 6.0E-03 | HDAC9 |
| 0.00148 | 0.90479 | 1.6E-02 | U2SURP |
| 0.00002 | 0.90834 | 5.0E-04 | MMADHC |
| 0.00036 | 0.91073 | 5.4E-03 | PPA1 |
| 0.00135 | 0.91098 | 1.5E-02 | EXOC5 |
| 0.00000 | 0.91324 | 4.2E-05 | DYNLT3 |
| 0.00000 | 0.91329 | 7.6E-05 | LEPROTL1 |
| 0.00052 | 0.91536 | 7.3E-03 | CLEC4A |
| 0.00003 | 0.91612 | 7.2E-04 | CLEC7A |
| 0.00005 | 0.91700 | 1.2E-03 | DERA |
| 0.00002 | 0.91740 | 6.8E-04 | PPP2R3C |
| 0.00004 | 0.91747 | 9.5E-04 | WSB2 |
| 0.00000 | 0.91807 | 1.4E-04 | RAB21 |
| 0.00002 | 0.91974 | 5.1E-04 | LSM14A |
| 0.00044 | 0.91996 | 6.4E-03 | COPS4 |
| 0.00054 | 0.92048 | 7.4E-03 | ENTPD4 |
| 0.00340 | 0.92070 | 2.9E-02 | PPM1D |
| 0.00490 | 0.92149 | 3.8E-02 | PTCD3 |
| 0.00278 | 0.92292 | 2.5E-02 | AGK |
| 0.00000 | 0.92442 | 8.4E-05 | KDM3A |
| 0.00062 | 0.92510 | 8.3E-03 | OPTN |
| 0.00035 | 0.92598 | 5.3E-03 | GZMA |
| 0.00001 | 0.92736 | 3.2E-04 | NMRK1 |
| 0.00004 | 0.92803 | 1.0E-03 | ZFP36L1 |
| 0.00656 | 0.92876 | 4.6E-02 | GADD45A |
| 0.00002 | 0.92931 | 5.1E-04 | PPP1CC |
| 0.00042 | 0.93126 | 6.1E-03 | APC |
| 0.00060 | 0.93294 | 8.1E-03 | CTC-428G20.3 |
| 0.00004 | 0.93298 | 1.0E-03 | GNAI3 |
| 0.00492 | 0.93362 | 3.8E-02 | ARID5A |
| 0.00003 | 0.93449 | 8.4E-04 | FNTA |
| 0.00000 | 0.93578 | 1.1E-04 | ARL6IP5 |
| 0.00324 | 0.93614 | 2.8E-02 | POLR2B |
| 0.00072 | 0.93751 | 9.3E-03 | ICE1 |
| 0.00206 | 0.93752 | 2.0E-02 | PPARA |
| 0.00336 | 0.93821 | 2.9E-02 | ADO |
| 0.00592 | 0.93849 | 4.3E-02 | MTR |
| 0.00277 | 0.93863 | 2.5E-02 | COA4 |
| 0.00001 | 0.93885 | 2.9E-04 | AQP9 |
| 0.00298 | 0.93909 | 2.7E-02 | S100A12 |
| 0.00001 | 0.93955 | 2.3E-04 | TM2D3 |
| 0.00000 | 0.94376 | 2.1E-05 | HECA |
| 0.00548 | 0.94416 | 4.1E-02 | STX7 |
| 0.00222 | 0.94436 | 2.2E-02 | PPP1R3D |
| 0.00671 | 0.94582 | 4.7E-02 | FUT4 |
| 0.00002 | 0.94703 | 6.9E-04 | EIF3E |
| 0.00065 | 0.94815 | 8.6E-03 | ACP1 |
| 0.00002 | 0.94910 | 6.1E-04 | MEGF9 |
| 0.00075 | 0.95028 | 9.6E-03 | IL18RAP |
| 0.00000 | 0.95075 | 2.0E-05 | RANBP9 |
| 0.00030 | 0.95131 | 4.7E-03 | NPAT |
| 0.00029 | 0.95131 | 4.6E-03 | C1GALT1C1 |
| 0.00001 | 0.95140 | 3.7E-04 | PTEN |
| 0.00002 | 0.95146 | 6.3E-04 | TLR1 |
| 0.00479 | 0.95192 | 3.7E-02 | GOLGA8CP |
| 0.00000 | 0.95193 | 2.0E-05 | CNBP |
| 0.00000 | 0.95237 | 9.0E-05 | ITM2B |
| 0.00416 | 0.95463 | 3.4E-02 | NDUFA9 |
| 0.00000 | 0.95528 | 8.7E-05 | PTGES3 |
| 0.00004 | 0.95600 | 9.3E-04 | ENOPH1 |
| 0.00390 | 0.95669 | 3.2E-02 | ORC2 |
| 0.00006 | 0.95914 | 1.4E-03 | SRP19 |
| 0.00001 | 0.95939 | 2.0E-04 | TRAPPC8 |
| 0.00125 | 0.95980 | 1.4E-02 | MAP3K7 |
| 0.00001 | 0.96210 | 2.4E-04 | TOMM20 |
| 0.00113 | 0.96291 | 1.3E-02 | MAOB |
| 0.00006 | 0.96320 | 1.3E-03 | HSP90AA1 |
| 0.00426 | 0.96416 | 3.4E-02 | RARS |
| 0.00482 | 0.96586 | 3.7E-02 | LOC100129361 |
| 0.00000 | 0.96614 | 7.5E-05 | ZCCHC6 |
| 0.00490 | 0.96684 | 3.8E-02 | RAB28 |
| 0.00000 | 0.96743 | 4.3E-05 | CCZ1 |
| 0.00313 | 0.96890 | 2.8E-02 | ZNF184 |
| 0.00029 | 0.97012 | 4.6E-03 | STAT1 |
| 0.00573 | 0.97013 | 4.2E-02 | FUBP3 |
| 0.00000 | 0.97111 | 1.5E-04 | SCP2 |
| 0.00000 | 0.97142 | 5.5E-05 | YY1 |
| 0.00107 | 0.97177 | 1.2E-02 | PPA2 |
| 0.00001 | 0.97232 | 2.7E-04 | PTEN |
| 0.00014 | 0.97296 | 2.7E-03 | RPAP3 |
| 0.00112 | 0.97337 | 1.3E-02 | WDR7 |
| 0.00026 | 0.97339 | 4.3E-03 | DMTF1 |
| 0.00000 | 0.97480 | 1.7E-04 | LYN |
| 0.00000 | 0.97523 | 1.5E-04 | DEGS1 |
| 0.00004 | 0.97570 | 1.1E-03 | ACSL1 |
| 0.00141 | 0.97608 | 1.5E-02 | DHX15 |
| 0.00212 | 0.97810 | 2.1E-02 | HLTF |
| 0.00006 | 0.97836 | 1.3E-03 | TOB1 |
| 0.00005 | 0.97863 | 1.1E-03 | TLE4 |
| 0.00014 | 0.97936 | 2.6E-03 | USP32 |
| 0.00005 | 0.97987 | 1.1E-03 | COX7A2 |
| 0.00093 | 0.98167 | 1.1E-02 | BCLAF1 |
| 0.00276 | 0.98179 | 2.5E-02 | ECHDC1 |
| 0.00000 | 0.98232 | 4.5E-05 | IQGAP2 |
| 0.00038 | 0.98259 | 5.7E-03 | DBI |
| 0.00001 | 0.98268 | 2.3E-04 | CHSY1 |
| 0.00000 | 0.98275 | 1.5E-05 | KCNJ2 |
| 0.00000 | 0.98543 | 8.5E-06 | SDHD |
| 0.00000 | 0.98644 | 3.3E-05 | LMBRD1 |
| 0.00644 | 0.98730 | 4.6E-02 | OSBPL8 |
| 0.00000 | 0.98820 | 1.8E-04 | CYB5R4 |
| 0.00000 | 0.98998 | 1.6E-05 | CRLF3 |
| 0.00441 | 0.99080 | 3.5E-02 | CUL4A |
| 0.00088 | 0.99095 | 1.1E-02 | NXT2 |
| 0.00000 | 0.99117 | 3.1E-05 | TCF12 |
| 0.00000 | 0.99154 | 1.0E-04 | SARAF |
| 0.00021 | 0.99324 | 3.7E-03 | ABHD5 |
| 0.00000 | 0.99425 | 1.6E-05 | STK17A |
| 0.00491 | 0.99576 | 3.8E-02 | RFX5 |
| 0.00046 | 0.99972 | 6.6E-03 | CDKN2AIP |
| 0.00598 | 1.00174 | 4.4E-02 | DDX1 |
| 0.00711 | 1.00241 | 4.9E-02 | PLEKHA2 |
| 0.00008 | 1.00365 | 1.8E-03 | IQGAP1 |
| 0.00004 | 1.00494 | 9.6E-04 | WDR47 |
| 0.00000 | 1.00822 | 4.7E-05 | SLC9A6 |
| 0.00000 | 1.01347 | 1.3E-04 | AK6 |
| 0.00000 | 1.01422 | 2.0E-04 | HSD17B11 |
| 0.00020 | 1.01431 | 3.5E-03 | SNX5 |
| 0.00246 | 1.01470 | 2.3E-02 | MCFD2 |
| 0.00369 | 1.01475 | 3.1E-02 | NEDD4 |
| 0.00151 | 1.01477 | 1.6E-02 | SRI |
| 0.00224 | 1.01675 | 2.2E-02 | PSMA2 |
| 0.00706 | 1.01720 | 4.9E-02 | RWDD1 |
| 0.00000 | 1.01741 | 6.7E-05 | ZFAND6 |
| 0.00306 | 1.01745 | 2.7E-02 | DIMT1 |
| 0.00059 | 1.01786 | 8.0E-03 | ATP13A3 |
| 0.00100 | 1.01788 | 1.2E-02 | SUCLA2 |
| 0.00001 | 1.01863 | 4.5E-04 | ANKRA2 |
| 0.00002 | 1.01880 | 5.2E-04 | ACTR3 |
| 0.00133 | 1.01900 | 1.5E-02 | C14orf2 |
| 0.00006 | 1.01992 | 1.3E-03 | MCMBP |
| 0.00019 | 1.02040 | 3.3E-03 | EIF4E |
| 0.00730 | 1.02199 | 5.0E-02 | LDLRAD4 |
| 0.00015 | 1.02372 | 2.8E-03 | C6orf120 |
| 0.00415 | 1.02493 | 3.4E-02 | LOC101928198 |
| 0.00091 | 1.02684 | 1.1E-02 | C1RL |
| 0.00010 | 1.02822 | 2.0E-03 | NAA16 |
| 0.00183 | 1.02893 | 1.9E-02 | TMEM50B |
| 0.00255 | 1.02920 | 2.4E-02 | HERC2P2 |
| 0.00003 | 1.03139 | 8.1E-04 | PPID |
| 0.00000 | 1.03491 | 1.1E-04 | RABGAP1L |
| 0.00000 | 1.03589 | 4.0E-05 | EXOC1 |
| 0.00001 | 1.03594 | 4.4E-04 | CHORDC1 |
| 0.00137 | 1.03787 | 1.5E-02 | TMEM33 |
| 0.00525 | 1.03955 | 4.0E-02 | DNAJC15 |
| 0.00085 | 1.03980 | 1.1E-02 | NAE1 |
| 0.00205 | 1.04043 | 2.0E-02 | ITGA6 |
| 0.00536 | 1.04157 | 4.0E-02 | XPA |
| 0.00000 | 1.04312 | 1.0E-06 | GOLGA7 |
| 0.00076 | 1.04364 | 9.7E-03 | GTF3C3 |
| 0.00260 | 1.04470 | 2.4E-02 | SHQ1 |
| 0.00000 | 1.04566 | 4.3E-05 | VPS26A |
| 0.00000 | 1.04817 | 5.7E-05 | MRPL3 |
| 0.00084 | 1.04945 | 1.0E-02 | UBP1 |
| 0.00036 | 1.04958 | 5.4E-03 | RBM7 |
| 0.00000 | 1.05031 | 5.3E-05 | CLK4 |
| 0.00002 | 1.05240 | 5.5E-04 | ZNF638 |
| 0.00092 | 1.05259 | 1.1E-02 | SLC33A1 |
| 0.00039 | 1.05512 | 5.8E-03 | TNFRSF10B |
| 0.00000 | 1.05669 | 7.3E-06 | TNFAIP8 |
| 0.00001 | 1.05808 | 2.2E-04 | CD36 |
| 0.00006 | 1.05985 | 1.4E-03 | ASCC3 |
| 0.00000 | 1.05995 | 1.7E-04 | SAT1 |
| 0.00033 | 1.06000 | 5.1E-03 | RAB1A |
| 0.00708 | 1.06315 | 4.9E-02 | PIK3R4 |
| 0.00000 | 1.06683 | 9.7E-05 | HIGD1A |
| 0.00156 | 1.06684 | 1.7E-02 | RMI1 |
| 0.00000 | 1.06738 | 1.8E-04 | TMED2 |
| 0.00146 | 1.06781 | 1.6E-02 | HEXIM1 |
| 0.00004 | 1.06921 | 9.9E-04 | CKLF |
| 0.00004 | 1.07045 | 1.0E-03 | PDE4B |
| 0.00001 | 1.07218 | 3.7E-04 | GPR183 |
| 0.00069 | 1.07398 | 9.0E-03 | AURKAPS1 |
| 0.00001 | 1.07412 | 4.3E-04 | FAM208A |
| 0.00002 | 1.07480 | 6.3E-04 | SS18L1 |
| 0.00224 | 1.07524 | 2.2E-02 | TNFAIP1 |
| 0.00000 | 1.07756 | 7.0E-05 | YPEL5 |
| 0.00064 | 1.07890 | 8.5E-03 | MON2 |
| 0.00381 | 1.07899 | 3.2E-02 | USP47 |
| 0.00000 | 1.08209 | 1.8E-04 | CD46 |
| 0.00009 | 1.08219 | 1.9E-03 | TBC1D15 |
| 0.00376 | 1.08571 | 3.1E-02 | FASTKD1 |
| 0.00382 | 1.08609 | 3.2E-02 | FAM172A |
| 0.00000 | 1.08628 | 6.8E-05 | FBXL5 |
| 0.00006 | 1.08681 | 1.3E-03 | SLTM |
| 0.00138 | 1.08694 | 1.5E-02 | IPO7 |
| 0.00010 | 1.08793 | 2.1E-03 | RPL23 |
| 0.00017 | 1.08852 | 3.0E-03 | MDM1 |
| 0.00241 | 1.08972 | 2.3E-02 | PSMD10 |
| 0.00016 | 1.09029 | 2.9E-03 | C12orf5 |
| 0.00000 | 1.09168 | 8.5E-06 | 09-Jan |
| 0.00440 | 1.09223 | 3.5E-02 | EEF1E1 |
| 0.00128 | 1.09263 | 1.4E-02 | RAB9A |
| 0.00001 | 1.09267 | 4.4E-04 | RAP1A |
| 0.00354 | 1.09308 | 3.0E-02 | EIF3J |
| 0.00215 | 1.09353 | 2.1E-02 | FAM208B |
| 0.00042 | 1.09435 | 6.2E-03 | RPS27 |
| 0.00000 | 1.09579 | 1.8E-04 | HNRNPH2 |
| 0.00000 | 1.09581 | 1.8E-04 | CLIP1 |
| 0.00000 | 1.09786 | 7.2E-05 | RAB14 |
| 0.00018 | 1.09832 | 3.2E-03 | UBXN2B |
| 0.00000 | 1.09902 | 1.8E-04 | PRPF39 |
| 0.00369 | 1.10112 | 3.1E-02 | GIMAP6 |
| 0.00000 | 1.10126 | 7.9E-05 | IVNS1ABP |
| 0.00001 | 1.10152 | 3.7E-04 | NRBF2 |
| 0.00000 | 1.10221 | 9.3E-05 | DPY30 |
| 0.00189 | 1.10246 | 1.9E-02 | NOC3L |
| 0.00286 | 1.10371 | 2.6E-02 | KCTD5 |
| 0.00000 | 1.10454 | 3.1E-05 | UBE2J1 |
| 0.00002 | 1.10722 | 5.9E-04 | RPL27 |
| 0.00630 | 1.10886 | 4.5E-02 | MSH2 |
| 0.00370 | 1.11008 | 3.1E-02 | UCHL5 |
| 0.00001 | 1.11045 | 2.4E-04 | TRIM22 |
| 0.00000 | 1.11118 | 1.6E-04 | CYCS |
| 0.00518 | 1.11190 | 3.9E-02 | MRE11A |
| 0.00000 | 1.11281 | 3.3E-06 | SIRT1 |
| 0.00001 | 1.11330 | 2.6E-04 | USP32P2 |
| 0.00001 | 1.11492 | 4.0E-04 | DLD |
| 0.00384 | 1.11509 | 3.2E-02 | LSM8 |
| 0.00031 | 1.11528 | 4.9E-03 | ARNTL |
| 0.00006 | 1.11581 | 1.4E-03 | TNFSF10 |
| 0.00378 | 1.11876 | 3.1E-02 | RCOR3 |
| 0.00002 | 1.12345 | 6.7E-04 | DNAJB6 |
| 0.00203 | 1.12345 | 2.0E-02 | PCNA |
| 0.00001 | 1.12974 | 4.3E-04 | N4BP2L2 |
| 0.00057 | 1.13222 | 7.8E-03 | PAXBP1 |
| 0.00000 | 1.13774 | 5.3E-07 | CEP170 |
| 0.00001 | 1.13839 | 2.7E-04 | STXBP3 |
| 0.00000 | 1.13866 | 8.7E-06 | CBFB |
| 0.00002 | 1.14049 | 5.9E-04 | ARL6IP1 |
| 0.00002 | 1.14086 | 6.9E-04 | RBM47 |
| 0.00091 | 1.14104 | 1.1E-02 | OGFRL1 |
| 0.00506 | 1.14140 | 3.9E-02 | ZNF432 |
| 0.00003 | 1.14189 | 8.4E-04 | PPP1R2 |
| 0.00040 | 1.14497 | 5.9E-03 | PGRMC1 |
| 0.00188 | 1.14579 | 1.9E-02 | CKLF |
| 0.00723 | 1.14669 | 5.0E-02 | TRAF5 |
| 0.00558 | 1.15023 | 4.1E-02 | FPGT |
| 0.00000 | 1.15025 | 1.9E-06 | NARS |
| 0.00000 | 1.15086 | 7.2E-06 | SRSF7 |
| 0.00000 | 1.15207 | 4.0E-05 | TBPL1 |
| 0.00151 | 1.15236 | 1.6E-02 | CLEC2D |
| 0.00140 | 1.15321 | 1.5E-02 | ATP10D |
| 0.00393 | 1.15371 | 3.2E-02 | CDR2 |
| 0.00000 | 1.15532 | 1.1E-05 | VEZF1 |
| 0.00088 | 1.15577 | 1.1E-02 | RPS17 |
| 0.00255 | 1.15591 | 2.4E-02 | WDR41 |
| 0.00000 | 1.15928 | 1.1E-05 | CCNC |
| 0.00026 | 1.16198 | 4.3E-03 | PDE4D |
| 0.00000 | 1.16297 | 3.4E-05 | ARL8B |
| 0.00001 | 1.16438 | 3.3E-04 | RNF11 |
| 0.00101 | 1.16447 | 1.2E-02 | CD52 |
| 0.00000 | 1.16507 | 5.3E-05 | PTPRC |
| 0.00000 | 1.16508 | 8.9E-05 | XPO1 |
| 0.00541 | 1.16536 | 4.1E-02 | EXOSC8 |
| 0.00018 | 1.16692 | 3.1E-03 | OSGIN2 |
| 0.00000 | 1.17020 | 2.3E-05 | RCBTB2 |
| 0.00186 | 1.17069 | 1.9E-02 | AHSA2 |
| 0.00004 | 1.17444 | 9.5E-04 | DHX29 |
| 0.00094 | 1.17618 | 1.1E-02 | DCUN1D4 |
| 0.00311 | 1.17791 | 2.8E-02 | HMGB1 |
| 0.00255 | 1.17823 | 2.4E-02 | NUP160 |
| 0.00067 | 1.17865 | 8.9E-03 | KIAA1598 |
| 0.00574 | 1.18158 | 4.2E-02 | RAD50 |
| 0.00398 | 1.18168 | 3.3E-02 | LINC00667 |
| 0.00723 | 1.18312 | 5.0E-02 | FAM184A |
| 0.00000 | 1.18471 | 7.6E-06 | ZNF638 |
| 0.00000 | 1.18646 | 5.3E-05 | MORF4L2 |
| 0.00000 | 1.19054 | 9.9E-06 | EIF4A2 |
| 0.00000 | 1.19110 | 6.7E-05 | NDFIP1 |
| 0.00041 | 1.19118 | 6.0E-03 | ABCC4 |
| 0.00037 | 1.19134 | 5.6E-03 | SLC30A5 |
| 0.00529 | 1.19274 | 4.0E-02 | VAV3 |
| 0.00684 | 1.19343 | 4.8E-02 | MFAP3 |
| 0.00410 | 1.19374 | 3.3E-02 | CROCCP2 |
| 0.00042 | 1.19379 | 6.2E-03 | ZNF302 |
| 0.00115 | 1.19586 | 1.3E-02 | AIDA |
| 0.00017 | 1.19646 | 3.0E-03 | RNF139 |
| 0.00083 | 1.19776 | 1.0E-02 | MBD2 |
|  |  |  |  |
| 0.00340 | 1.19969 | 2.9E-02 | NHLRC2 |
| 0.00007 | 1.19997 | 1.5E-03 | WDR11 |
| 0.00000 | 1.20038 | 5.3E-05 | C11orf58 |
| 0.00134 | 1.20119 | 1.5E-02 | MED17 |
| 0.00000 | 1.20182 | 2.5E-05 | C6orf62 |
| 0.00127 | 1.20361 | 1.4E-02 | LRIG1 |
| 0.00028 | 1.20823 | 4.5E-03 | PPP3CB |
| 0.00000 | 1.20844 | 6.2E-07 | VAMP7 |
| 0.00001 | 1.21068 | 2.8E-04 | BTBD1 |
| 0.00336 | 1.21170 | 2.9E-02 | RIN2 |
| 0.00371 | 1.21174 | 3.1E-02 | DLEU2 |
| 0.00196 | 1.21272 | 2.0E-02 | MRPS31 |
| 0.00014 | 1.21392 | 2.6E-03 | UBL5 |
| 0.00100 | 1.21553 | 1.2E-02 | CEP63 |
| 0.00081 | 1.21835 | 1.0E-02 | TTC13 |
| 0.00555 | 1.21836 | 4.1E-02 | PIGA |
| 0.00000 | 1.21935 | 4.6E-07 | TBK1 |
| 0.00015 | 1.22052 | 2.7E-03 | CRYZ |
| 0.00004 | 1.22137 | 9.8E-04 | RPL7 |
| 0.00001 | 1.22582 | 4.4E-04 | TSNAX |
| 0.00000 | 1.23166 | 2.4E-05 | NPTN |
| 0.00000 | 1.23174 | 1.7E-04 | ZMPSTE24 |
| 0.00045 | 1.23495 | 6.5E-03 | ALG13 |
| 0.00091 | 1.23604 | 1.1E-02 | ETF1 |
| 0.00000 | 1.23614 | 1.6E-05 | RNF111 |
| 0.00000 | 1.23827 | 6.1E-06 | RASA1 |
| 0.00267 | 1.23945 | 2.5E-02 | GPR171 |
| 0.00000 | 1.24019 | 1.2E-05 | GCC2 |
| 0.00061 | 1.24032 | 8.2E-03 | UNC50 |
| 0.00000 | 1.24108 | 1.7E-05 | ANKRD10 |
| 0.00025 | 1.24385 | 4.2E-03 | CCNT2 |
| 0.00023 | 1.24479 | 3.8E-03 | SAMD9 |
| 0.00036 | 1.24576 | 5.4E-03 | ICE2 |
| 0.00057 | 1.24710 | 7.9E-03 | PTGER2 |
| 0.00016 | 1.24776 | 3.0E-03 | SELT |
| 0.00004 | 1.25008 | 1.0E-03 | BCL10 |
| 0.00037 | 1.25047 | 5.5E-03 | COX7C |
| 0.00000 | 1.25093 | 2.7E-06 | NFE2L2 |
| 0.00006 | 1.25121 | 1.3E-03 | KPNA3 |
| 0.00000 | 1.25327 | 6.7E-06 | ITGB1 |
| 0.00000 | 1.25397 | 2.1E-06 | PPM1B |
| 0.00014 | 1.25426 | 2.7E-03 | SUCLG2 |
| 0.00000 | 1.25538 | 1.3E-05 | UBE2E1 |
| 0.00089 | 1.25611 | 1.1E-02 | DERL1 |
| 0.00012 | 1.25629 | 2.4E-03 | HSPA13 |
| 0.00001 | 1.25673 | 3.9E-04 | SLC39A6 |
| 0.00001 | 1.25892 | 3.2E-04 | SSFA2 |
| 0.00000 | 1.25989 | 1.7E-05 | TOR1AIP1 |
| 0.00067 | 1.26001 | 8.9E-03 | LOC100506639 |
| 0.00029 | 1.26065 | 4.6E-03 | RPL15 |
| 0.00490 | 1.26397 | 3.8E-02 | UFSP2 |
| 0.00000 | 1.26441 | 2.4E-05 | PRNP |
| 0.00154 | 1.26472 | 1.6E-02 | CEP162 |
| 0.00107 | 1.26513 | 1.2E-02 | PICALM |
| 0.00000 | 1.26726 | 9.9E-06 | CTSC |
| 0.00214 | 1.26794 | 2.1E-02 | NDUFB7 |
| 0.00016 | 1.26889 | 3.0E-03 | ITPR1 |
| 0.00053 | 1.26919 | 7.3E-03 | PRPF38B |
| 0.00132 | 1.26932 | 1.5E-02 | CAPN7 |
| 0.00450 | 1.26933 | 3.6E-02 | EPS8 |
| 0.00352 | 1.26986 | 3.0E-02 | MILR1 |
| 0.00000 | 1.27043 | 3.8E-05 | ANXA1 |
| 0.00089 | 1.27095 | 1.1E-02 | ZEB2 |
| 0.00006 | 1.27155 | 1.5E-03 | CSNK1G3 |
| 0.00466 | 1.27254 | 3.6E-02 | PDHB |
| 0.00036 | 1.27574 | 5.5E-03 | MATR3 |
| 0.00003 | 1.27583 | 9.0E-04 | ENPP4 |
| 0.00120 | 1.27628 | 1.4E-02 | PREPL |
| 0.00000 | 1.27699 | 9.8E-06 | GSAP |
| 0.00049 | 1.27797 | 6.9E-03 | DNAJA2 |
| 0.00000 | 1.27874 | 6.8E-05 | BIRC2 |
| 0.00009 | 1.28022 | 1.9E-03 | TOPORS |
| 0.00001 | 1.28071 | 2.2E-04 | ITM2A |
| 0.00351 | 1.28602 | 3.0E-02 | ROCK2 |
| 0.00496 | 1.28688 | 3.8E-02 | PSMD5 |
| 0.00000 | 1.28717 | 3.3E-05 | SHOC2 |
| 0.00233 | 1.28835 | 2.2E-02 | APPBP2 |
| 0.00000 | 1.28933 | 2.4E-06 | TCERG1 |
| 0.00000 | 1.28952 | 1.3E-06 | PNRC2 |
| 0.00000 | 1.29166 | 4.9E-05 | NMI |
| 0.00000 | 1.29230 | 4.1E-06 | SNX2 |
| 0.00322 | 1.29239 | 2.8E-02 | PMPCB |
| 0.00000 | 1.29308 | 2.8E-05 | LAMP2 |
| 0.00000 | 1.29450 | 3.8E-06 | HNRNPA3 |
| 0.00000 | 1.29537 | 1.7E-06 | DYRK1A |
| 0.00000 | 1.29963 | 1.3E-06 | FAM8A1 |
| 0.00157 | 1.30009 | 1.7E-02 | SKP2 |
| 0.00001 | 1.30095 | 2.3E-04 | FMR1 |
| 0.00330 | 1.30267 | 2.9E-02 | ZNF322 |
| 0.00318 | 1.30302 | 2.8E-02 | KPNA1 |
| 0.00001 | 1.30416 | 4.1E-04 | CTSS |
| 0.00000 | 1.30542 | 9.7E-07 | CDKN1B |
| 0.00299 | 1.30758 | 2.7E-02 | KRBOX4 |
| 0.00000 | 1.30884 | 5.5E-05 | PRKRIR |
| 0.00358 | 1.31033 | 3.0E-02 | CDC37L1 |
| 0.00155 | 1.31081 | 1.6E-02 | CSF2RA |
| 0.00269 | 1.31239 | 2.5E-02 | SMURF2 |
| 0.00043 | 1.31373 | 6.3E-03 | SLC2A3 |
| 0.00001 | 1.31668 | 3.4E-04 | EED |
| 0.00053 | 1.31882 | 7.3E-03 | RPL39 |
| 0.00003 | 1.31913 | 7.7E-04 | NECAP1 |
| 0.00000 | 1.31945 | 4.6E-05 | SEC24B |
| 0.00000 | 1.32127 | 1.9E-04 | FLI1 |
| 0.00087 | 1.32266 | 1.1E-02 | PLAGL1 |
| 0.00000 | 1.32429 | 6.4E-06 | PRKACB |
| 0.00000 | 1.32502 | 1.6E-05 | SRGN |
| 0.00149 | 1.32510 | 1.6E-02 | GBE1 |
| 0.00000 | 1.32614 | 3.8E-06 | PCMT1 |
| 0.00154 | 1.32673 | 1.6E-02 | AASDHPPT |
| 0.00000 | 1.32810 | 3.9E-06 | C5orf15 |
| 0.00231 | 1.33273 | 2.2E-02 | TMF1 |
| 0.00142 | 1.33340 | 1.5E-02 | ITGAV |
| 0.00165 | 1.33408 | 1.7E-02 | FCAR |
| 0.00000 | 1.33416 | 8.5E-06 | ZNF217 |
| 0.00008 | 1.33529 | 1.7E-03 | GALNT1 |
| 0.00000 | 1.33681 | 1.4E-04 | RBPJ |
| 0.00009 | 1.33712 | 1.9E-03 | DBF4 |
| 0.00031 | 1.33726 | 4.8E-03 | TMEM156 |
| 0.00000 | 1.33979 | 9.8E-06 | DMXL2 |
| 0.00022 | 1.34147 | 3.7E-03 | AGPAT5 |
| 0.00001 | 1.34218 | 4.5E-04 | RPS17P5 |
| 0.00000 | 1.34326 | 1.0E-05 | MED23 |
| 0.00510 | 1.34623 | 3.9E-02 | MRPS28 |
| 0.00000 | 1.34883 | 1.4E-06 | VPS4B |
| 0.00123 | 1.34887 | 1.4E-02 | TIMP4 |
| 0.00492 | 1.34930 | 3.8E-02 | NNT |
| 0.00002 | 1.35008 | 6.2E-04 | PSIP1 |
| 0.00004 | 1.35173 | 9.6E-04 | FAM35A |
| 0.00093 | 1.35215 | 1.1E-02 | DPY19L1 |
| 0.00190 | 1.35278 | 1.9E-02 | SLC16A1 |
| 0.00173 | 1.35611 | 1.8E-02 | CRIPT |
| 0.00000 | 1.35644 | 1.6E-05 | GCH1 |
| 0.00190 | 1.35823 | 1.9E-02 | ZNF451 |
| 0.00153 | 1.35981 | 1.6E-02 | FAM224A |
| 0.00000 | 1.36377 | 1.0E-04 | NAA50 |
| 0.00000 | 1.36530 | 5.2E-05 | DCK |
| 0.00005 | 1.36629 | 1.3E-03 | CDYL |
| 0.00000 | 1.36765 | 8.7E-05 | PEX2 |
| 0.00000 | 1.36879 | 1.4E-06 | OSBPL11 |
| 0.00137 | 1.37022 | 1.5E-02 | UBA2 |
| 0.00005 | 1.37054 | 1.2E-03 | TAF2 |
| 0.00000 | 1.37069 | 1.4E-04 | CREG1 |
| 0.00000 | 1.37105 | 1.2E-04 | ARAP2 |
| 0.00395 | 1.37219 | 3.2E-02 | KLHL20 |
| 0.00090 | 1.37252 | 1.1E-02 | CATSPERB |
| 0.00000 | 1.37412 | 7.5E-06 | DNAJA1 |
| 0.00103 | 1.37474 | 1.2E-02 | TMEM106B |
| 0.00369 | 1.37837 | 3.1E-02 | SEC23IP |
| 0.00000 | 1.37999 | 3.8E-06 | ADAM9 |
| 0.00072 | 1.38129 | 9.3E-03 | RNF170 |
| 0.00007 | 1.38239 | 1.6E-03 | RPS24 |
| 0.00001 | 1.38296 | 3.3E-04 | CD58 |
| 0.00036 | 1.38459 | 5.4E-03 | COPS8 |
| 0.00098 | 1.38510 | 1.2E-02 | PIK3CA |
| 0.00011 | 1.38944 | 2.3E-03 | ZZZ3 |
| 0.00000 | 1.39050 | 3.7E-05 | RSBN1 |
| 0.00214 | 1.39314 | 2.1E-02 | CHD9 |
| 0.00078 | 1.39315 | 9.9E-03 | CBWD1 |
| 0.00373 | 1.39591 | 3.1E-02 | ZFAND1 |
| 0.00000 | 1.39602 | 4.9E-05 | UBE2D3 |
| 0.00424 | 1.39731 | 3.4E-02 | CAMP |
| 0.00041 | 1.39732 | 6.0E-03 | RPS29 |
| 0.00025 | 1.39781 | 4.2E-03 | TXNDC9 |
| 0.00003 | 1.40109 | 8.9E-04 | UQCRQ |
| 0.00406 | 1.40659 | 3.3E-02 | NABP1 |
| 0.00022 | 1.40719 | 3.7E-03 | RABGGTB |
| 0.00003 | 1.40815 | 7.1E-04 | WDR44 |
| 0.00007 | 1.40877 | 1.5E-03 | TMX1 |
| 0.00000 | 1.41267 | 5.3E-07 | TLK1 |
| 0.00017 | 1.41280 | 3.0E-03 | CDADC1 |
| 0.00093 | 1.41400 | 1.1E-02 | GMFB |
| 0.00105 | 1.41565 | 1.2E-02 | MCL1 |
| 0.00207 | 1.41640 | 2.1E-02 | PTBP2 |
| 0.00000 | 1.41689 | 1.1E-04 | VNN2 |
| 0.00187 | 1.41846 | 1.9E-02 | DPY19L2P2 |
| 0.00012 | 1.41892 | 2.3E-03 | TDG |
| 0.00003 | 1.42034 | 7.6E-04 | KATNBL1 |
| 0.00000 | 1.42568 | 3.6E-06 | PNISR |
| 0.00010 | 1.42610 | 2.0E-03 | WAPAL |
| 0.00006 | 1.42734 | 1.4E-03 | LIN7C |
| 0.00001 | 1.42946 | 3.6E-04 | RHOT1 |
| 0.00000 | 1.43016 | 1.6E-04 | MTMR6 |
| 0.00029 | 1.43048 | 4.7E-03 | LOC101928189 |
| 0.00017 | 1.43052 | 3.0E-03 | NR2C1 |
| 0.00002 | 1.43188 | 5.0E-04 | ZNF200 |
| 0.00283 | 1.43290 | 2.6E-02 | FASTKD2 |
| 0.00017 | 1.43408 | 3.1E-03 | KANSL2 |
| 0.00073 | 1.43595 | 9.3E-03 | USP33 |
| 0.00285 | 1.43737 | 2.6E-02 | LAMP1 |
| 0.00057 | 1.43802 | 7.8E-03 | ARHGAP12 |
| 0.00281 | 1.44054 | 2.6E-02 | MALT1 |
| 0.00000 | 1.44128 | 1.3E-06 | CUL5 |
| 0.00000 | 1.44201 | 1.4E-06 | RAP1B |
| 0.00000 | 1.44521 | 2.1E-06 | TDP2 |
| 0.00002 | 1.44626 | 6.2E-04 | SERP1 |
| 0.00000 | 1.44630 | 2.7E-05 | LY75 |
| 0.00000 | 1.44954 | 2.5E-05 | RBL2 |
| 0.00006 | 1.44996 | 1.3E-03 | RPS7 |
| 0.00066 | 1.45013 | 8.7E-03 | RBL1 |
| 0.00000 | 1.45084 | 1.6E-04 | RSL24D1 |
| 0.00319 | 1.45388 | 2.8E-02 | TRMT11 |
| 0.00001 | 1.45440 | 3.4E-04 | STK17B |
| 0.00038 | 1.45457 | 5.7E-03 | DYNC1LI1 |
| 0.00391 | 1.45534 | 3.2E-02 | FDX1 |
| 0.00007 | 1.45543 | 1.5E-03 | COMMD10 |
| 0.00002 | 1.45554 | 5.6E-04 | UBE2N |
| 0.00433 | 1.45614 | 3.5E-02 | TTC33 |
| 0.00253 | 1.45889 | 2.4E-02 | HSPA14 |
| 0.00037 | 1.46004 | 5.5E-03 | AP1S2 |
| 0.00086 | 1.46050 | 1.1E-02 | ZNF354A |
| 0.00005 | 1.46126 | 1.2E-03 | CPD |
| 0.00073 | 1.46175 | 9.4E-03 | MAPK6 |
| 0.00030 | 1.46235 | 4.8E-03 | MRPL42 |
| 0.00506 | 1.46259 | 3.9E-02 | MAPK9 |
| 0.00028 | 1.46267 | 4.5E-03 | TBC1D4 |
| 0.00384 | 1.46349 | 3.2E-02 | HEATR3 |
| 0.00001 | 1.46407 | 3.0E-04 | SUZ12 |
| 0.00156 | 1.46445 | 1.7E-02 | ZNHIT6 |
| 0.00000 | 1.46474 | 3.7E-05 | BIRC3 |
| 0.00272 | 1.46571 | 2.5E-02 | ABCD3 |
| 0.00004 | 1.46871 | 1.0E-03 | TMCO1 |
| 0.00268 | 1.46878 | 2.5E-02 | IFT57 |
| 0.00551 | 1.46901 | 4.1E-02 | BCAP29 |
| 0.00000 | 1.47220 | 6.1E-06 | LPGAT1 |
| 0.00007 | 1.47744 | 1.5E-03 | UPF3A |
| 0.00101 | 1.47860 | 1.2E-02 | ANK3 |
| 0.00009 | 1.47999 | 1.9E-03 | RPS15A |
| 0.00096 | 1.48009 | 1.2E-02 | CCDC91 |
| 0.00000 | 1.48125 | 2.5E-07 | GALC |
| 0.00000 | 1.48185 | 7.5E-06 | CD55 |
| 0.00000 | 1.48309 | 4.5E-07 | CBX3 |
| 0.00029 | 1.48596 | 4.7E-03 | PRDX3 |
| 0.00000 | 1.48633 | 1.8E-05 | CD302 |
| 0.00000 | 1.48648 | 3.8E-06 | DDX3X |
| 0.00012 | 1.49166 | 2.4E-03 | UPF3B |
| 0.00034 | 1.49307 | 5.2E-03 | CLASP2 |
| 0.00000 | 1.49653 | 5.1E-07 | DCP2 |
| 0.00000 | 1.50040 | 2.1E-06 | FAS |
| 0.00001 | 1.50991 | 2.4E-04 | SLC25A46 |
| 0.00015 | 1.51023 | 2.8E-03 | SLC25A32 |
| 0.00099 | 1.51035 | 1.2E-02 | SCML1 |
| 0.00011 | 1.51298 | 2.2E-03 | RIOK3 |
| 0.00330 | 1.51455 | 2.9E-02 | NEK4 |
| 0.00023 | 1.51851 | 3.9E-03 | RSF1 |
| 0.00001 | 1.52192 | 2.9E-04 | NEMF |
| 0.00000 | 1.52202 | 2.1E-06 | AKAP11 |
| 0.00068 | 1.52261 | 8.9E-03 | CD69 |
| 0.00001 | 1.52391 | 3.3E-04 | XPOT |
| 0.00000 | 1.52598 | 7.2E-06 | ELOVL5 |
| 0.00217 | 1.52649 | 2.1E-02 | KLRAP1 |
| 0.00070 | 1.52800 | 9.1E-03 | CEBPZ |
| 0.00017 | 1.53185 | 3.1E-03 | ERV3-2 |
| 0.00006 | 1.53217 | 1.3E-03 | VCPKMT |
| 0.00031 | 1.53227 | 4.8E-03 | COX7BP1 |
| 0.00000 | 1.53419 | 1.3E-05 | GCA |
| 0.00093 | 1.53507 | 1.1E-02 | PSMA4 |
| 0.00010 | 1.53620 | 2.0E-03 | ZNF230 |
| 0.00026 | 1.53731 | 4.3E-03 | RPL26L1 |
| 0.00047 | 1.53886 | 6.7E-03 | SEC24A |
| 0.00050 | 1.54090 | 6.9E-03 | RPL34 |
| 0.00211 | 1.54213 | 2.1E-02 | GTF2H1 |
| 0.00000 | 1.54333 | 4.7E-06 | BACH1 |
| 0.00000 | 1.54450 | 1.2E-06 | PDCD10 |
| 0.00077 | 1.55319 | 9.8E-03 | BST1 |
| 0.00609 | 1.55396 | 4.4E-02 | ICK |
| 0.00146 | 1.55473 | 1.6E-02 | CCPG1 |
| 0.00000 | 1.55732 | 1.2E-05 | CCNG2 |
| 0.00000 | 1.55919 | 1.1E-06 | MIR6125 |
| 0.00006 | 1.55935 | 1.4E-03 | TAF5 |
| 0.00029 | 1.56136 | 4.6E-03 | RPS10P2 |
| 0.00034 | 1.56302 | 5.2E-03 | VNN1 |
| 0.00030 | 1.56382 | 4.7E-03 | THAP9-AS1 |
| 0.00002 | 1.56441 | 4.9E-04 | TBC1D9 |
| 0.00122 | 1.56697 | 1.4E-02 | PIGK |
| 0.00019 | 1.56936 | 3.4E-03 | RPS27L |
| 0.00000 | 1.56982 | 1.4E-04 | RFK |
| 0.00092 | 1.57007 | 1.1E-02 | PELI2 |
| 0.00001 | 1.57518 | 4.3E-04 | RAN |
| 0.00000 | 1.57729 | 1.6E-05 | IBTK |
| 0.00222 | 1.57889 | 2.2E-02 | CMAS |
| 0.00351 | 1.58184 | 3.0E-02 | RTCA |
| 0.00000 | 1.58395 | 1.4E-04 | LAMTOR3 |
| 0.00000 | 1.58610 | 2.0E-06 | CTBS |
| 0.00048 | 1.58641 | 6.8E-03 | EIF2S3 |
| 0.00000 | 1.59144 | 5.1E-06 | TAF7 |
| 0.00022 | 1.59342 | 3.7E-03 | ASUN |
| 0.00292 | 1.59552 | 2.6E-02 | F2R |
| 0.00050 | 1.59644 | 7.0E-03 | SGMS1 |
| 0.00390 | 1.59654 | 3.2E-02 | NDUFA5 |
| 0.00130 | 1.59699 | 1.5E-02 | FOS |
| 0.00099 | 1.59768 | 1.2E-02 | MAP4K5 |
| 0.00040 | 1.59847 | 5.9E-03 | RINT1 |
| 0.00096 | 1.59863 | 1.2E-02 | DOCK4 |
| 0.00000 | 1.60285 | 1.2E-06 | MDFIC |
| 0.00008 | 1.60341 | 1.6E-03 | CEP192 |
| 0.00044 | 1.60623 | 6.4E-03 | MYB |
| 0.00002 | 1.60639 | 4.8E-04 | ANKRD46 |
| 0.00402 | 1.60667 | 3.3E-02 | GTF2H2 |
| 0.00000 | 1.60776 | 4.7E-07 | HIF1A |
| 0.00183 | 1.60819 | 1.9E-02 | TMEM38B |
| 0.00378 | 1.60851 | 3.1E-02 | NAIP |
| 0.00014 | 1.60986 | 2.6E-03 | CPOX |
| 0.00002 | 1.61437 | 4.7E-04 | TCFL5 |
| 0.00000 | 1.61445 | 3.1E-06 | CD164 |
| 0.00011 | 1.61483 | 2.3E-03 | LRIF1 |
| 0.00015 | 1.61549 | 2.8E-03 | VPS54 |
| 0.00000 | 1.61620 | 1.9E-04 | FBXO28 |
| 0.00080 | 1.61671 | 1.0E-02 | AZI2 |
| 0.00000 | 1.61675 | 2.1E-06 | TRAM1 |
| 0.00000 | 1.61785 | 3.7E-05 | SAMSN1 |
| 0.00055 | 1.61787 | 7.6E-03 | FAM3C |
| 0.00058 | 1.62136 | 7.9E-03 | TRAT1 |
| 0.00000 | 1.62387 | 8.1E-05 | BNIP3L |
| 0.00004 | 1.62796 | 9.9E-04 | PTPN22 |
| 0.00018 | 1.63171 | 3.2E-03 | DLG1 |
| 0.00000 | 1.63206 | 9.0E-07 | PRKAR1A |
| 0.00000 | 1.63304 | 1.7E-05 | SPTLC1 |
| 0.00114 | 1.63318 | 1.3E-02 | RRN3 |
| 0.00000 | 1.63502 | 1.2E-04 | DSE |
| 0.00001 | 1.63586 | 4.3E-04 | BCL2A1 |
| 0.00000 | 1.63864 | 2.6E-05 | LY96 |
| 0.00006 | 1.64065 | 1.3E-03 | LOC102724364 |
| 0.00000 | 1.64223 | 4.7E-07 | DPYD |
| 0.00036 | 1.64511 | 5.4E-03 | ZNHIT3 |
| 0.00003 | 1.64677 | 8.5E-04 | GNPAT |
| 0.00000 | 1.64677 | 7.3E-08 | EFR3A |
| 0.00265 | 1.64969 | 2.4E-02 | ALDH5A1 |
| 0.00073 | 1.65158 | 9.4E-03 | ACADM |
| 0.00000 | 1.65217 | 6.1E-07 | CAB39 |
| 0.00004 | 1.65494 | 1.0E-03 | PYROXD1 |
| 0.00000 | 1.65562 | 1.4E-06 | MORC3 |
| 0.00010 | 1.65595 | 2.1E-03 | IFIT5 |
| 0.00000 | 1.65797 | 6.7E-05 | S100A8 |
| 0.00000 | 1.66053 | 1.4E-04 | OSTM1 |
| 0.00000 | 1.66086 | 1.7E-07 | VPS13C |
| 0.00085 | 1.66298 | 1.1E-02 | GK3P |
| 0.00010 | 1.66690 | 2.1E-03 | GRAMD1C |
| 0.00000 | 1.66876 | 1.3E-06 | GPR65 |
| 0.00034 | 1.66881 | 5.2E-03 | STAG1 |
| 0.00001 | 1.67067 | 4.4E-04 | ABI1 |
| 0.00000 | 1.67215 | 7.6E-05 | ZBED5 |
| 0.00040 | 1.67283 | 6.0E-03 | EIF1AX |
| 0.00188 | 1.67285 | 1.9E-02 | MS4A3 |
| 0.00000 | 1.67438 | 7.6E-06 | TMED5 |
| 0.00000 | 1.67447 | 2.7E-05 | PTPN12 |
| 0.00005 | 1.67456 | 1.2E-03 | FAM13B |
| 0.00030 | 1.67661 | 4.7E-03 | ST3GAL6 |
| 0.00494 | 1.67676 | 3.8E-02 | BLZF1 |
| 0.00000 | 1.67814 | 4.7E-07 | IFNGR1 |
| 0.00001 | 1.68134 | 4.6E-04 | RBM3 |
| 0.00000 | 1.68257 | 6.9E-06 | UBE2D1 |
| 0.00025 | 1.68523 | 4.1E-03 | GBAS |
| 0.00010 | 1.68624 | 2.0E-03 | SLC19A2 |
| 0.00001 | 1.68650 | 3.1E-04 | AK022030 |
| 0.00067 | 1.69562 | 8.9E-03 | YAF2 |
| 0.00002 | 1.69890 | 5.3E-04 | RCN2 |
| 0.00000 | 1.70403 | 6.2E-07 | PUM2 |
| 0.00000 | 1.70447 | 6.2E-07 | RBM39 |
| 0.00012 | 1.70605 | 2.3E-03 | PKD2 |
| 0.00014 | 1.70967 | 2.7E-03 | CCNG1 |
| 0.00008 | 1.70975 | 1.7E-03 | SMC4 |
| 0.00012 | 1.71171 | 2.4E-03 | DNAJB4 |
| 0.00273 | 1.71191 | 2.5E-02 | GOLGA6L4 |
| 0.00007 | 1.71587 | 1.6E-03 | UBL3 |
| 0.00071 | 1.71793 | 9.2E-03 | PPWD1 |
| 0.00003 | 1.72300 | 7.7E-04 | VBP1 |
| 0.00000 | 1.72899 | 9.9E-07 | TMEM30A |
| 0.00000 | 1.72936 | 1.8E-04 | EPS15 |
| 0.00028 | 1.73018 | 4.5E-03 | BOLA2 |
| 0.00004 | 1.73075 | 9.9E-04 | MRPL13 |
| 0.00001 | 1.73187 | 3.1E-04 | SMC6 |
| 0.00000 | 1.73627 | 2.1E-06 | CAPZA1 |
| 0.00016 | 1.73841 | 3.0E-03 | NIF3L1 |
| 0.00000 | 1.74082 | 6.8E-06 | KLRF1 |
| 0.00217 | 1.74187 | 2.1E-02 | HGF |
| 0.00000 | 1.74380 | 9.0E-07 | RB1CC1 |
| 0.00000 | 1.74533 | 2.5E-05 | INTS8 |
| 0.00000 | 1.74677 | 6.1E-07 | ZFAND5 |
| 0.00013 | 1.74849 | 2.5E-03 | DNAJC10 |
| 0.00042 | 1.74936 | 6.1E-03 | GOLT1B |
| 0.00000 | 1.75805 | 1.5E-04 | SMNDC1 |
| 0.00000 | 1.76322 | 9.0E-05 | ZNF292 |
| 0.00006 | 1.76629 | 1.4E-03 | HSDL2 |
| 0.00000 | 1.76711 | 6.6E-05 | ELF1 |
| 0.00000 | 1.76787 | 4.7E-07 | LUC7L3 |
| 0.00011 | 1.76895 | 2.1E-03 | CNIH4 |
| 0.00000 | 1.77297 | 6.1E-07 | LPAR6 |
| 0.00001 | 1.79295 | 4.1E-04 | UBA6 |
| 0.00000 | 1.79307 | 4.7E-07 | KIAA1551 |
| 0.00006 | 1.79543 | 1.4E-03 | SAR1B |
| 0.00157 | 1.80010 | 1.7E-02 | POLI |
| 0.00002 | 1.80113 | 5.3E-04 | ATP11B |
| 0.00000 | 1.80204 | 1.9E-04 | PDS5B |
| 0.00003 | 1.80255 | 8.7E-04 | PJA2 |
| 0.00003 | 1.80506 | 8.8E-04 | GALNT7 |
| 0.00000 | 1.80659 | 4.0E-05 | TRIM37 |
| 0.00000 | 1.81196 | 1.9E-04 | CAPZA2 |
| 0.00001 | 1.81508 | 3.3E-04 | BTAF1 |
| 0.00008 | 1.81729 | 1.7E-03 | LEMD3 |
| 0.00000 | 1.81769 | 1.2E-04 | KLHL2 |
| 0.00000 | 1.82025 | 5.8E-06 | HMGB2 |
| 0.00000 | 1.83158 | 5.7E-05 | SUB1 |
| 0.00001 | 1.83162 | 3.6E-04 | PIK3C2A |
| 0.00016 | 1.83769 | 2.9E-03 | IMPACT |
| 0.00000 | 1.83995 | 1.1E-06 | JMJD1C |
| 0.00000 | 1.84115 | 1.7E-04 | TRIM52 |
| 0.00000 | 1.84157 | 6.4E-06 | SERINC1 |
| 0.00001 | 1.84764 | 3.1E-04 | CHD1 |
| 0.00017 | 1.84776 | 3.1E-03 | GALNT3 |
| 0.00001 | 1.84896 | 4.4E-04 | IDI1 |
| 0.00033 | 1.85345 | 5.0E-03 | EBAG9 |
| 0.00000 | 1.86121 | 2.1E-06 | ZBTB11 |
| 0.00005 | 1.86377 | 1.3E-03 | RANBP6 |
| 0.00001 | 1.86850 | 2.4E-04 | PRPF4B |
| 0.00015 | 1.87238 | 2.7E-03 | SLC30A1 |
| 0.00001 | 1.87829 | 4.4E-04 | ITPR2 |
| 0.00011 | 1.88658 | 2.3E-03 | TFEC |
| 0.00000 | 1.88754 | 4.5E-06 | LYST |
| 0.00005 | 1.89077 | 1.3E-03 | MFN1 |
| 0.00000 | 1.89146 | 1.8E-07 | SRP9 |
| 0.00000 | 1.89164 | 2.8E-07 | ADAM10 |
| 0.00049 | 1.90315 | 6.9E-03 | MSMO1 |
| 0.00000 | 1.90576 | 8.6E-05 | YTHDF3 |
| 0.00000 | 1.90617 | 3.3E-08 | ADD3 |
| 0.00001 | 1.90909 | 2.4E-04 | RCBTB1 |
| 0.00016 | 1.91106 | 3.0E-03 | MTERF3 |
| 0.00003 | 1.91118 | 7.9E-04 | GK |
| 0.00009 | 1.91392 | 1.9E-03 | CSGALNACT1 |
| 0.00000 | 1.91580 | 1.8E-05 | UBXN4 |
| 0.00004 | 1.91690 | 9.2E-04 | SENP6 |
| 0.00000 | 1.92087 | 5.8E-06 | RAB33B |
| 0.00010 | 1.92179 | 2.1E-03 | FBXO3 |
| 0.00146 | 1.92547 | 1.6E-02 | FASTKD3 |
| 0.00001 | 1.92583 | 3.0E-04 | RP2 |
| 0.00000 | 1.92681 | 1.6E-04 | INSIG2 |
| 0.00012 | 1.92884 | 2.4E-03 | ZNF140 |
| 0.00000 | 1.93090 | 7.0E-05 | DPM1 |
| 0.00006 | 1.93090 | 1.3E-03 | KAT2B |
| 0.00000 | 1.93210 | 2.0E-05 | TBL1XR1 |
| 0.00008 | 1.93336 | 1.7E-03 | ZMYM2 |
| 0.00000 | 1.93702 | 3.2E-05 | C18orf32 |
| 0.00000 | 1.93890 | 2.6E-06 | ROCK1 |
| 0.00000 | 1.94179 | 9.9E-05 | BNIP2 |
| 0.00001 | 1.94239 | 3.9E-04 | REV3L |
| 0.00001 | 1.95293 | 2.6E-04 | SREK1 |
| 0.00005 | 1.95475 | 1.3E-03 | AKAP7 |
| 0.00008 | 1.95643 | 1.7E-03 | IL1R1 |
| 0.00000 | 1.95667 | 3.1E-05 | IMPA1 |
| 0.00000 | 1.95703 | 5.2E-08 | PLEKHF2 |
| 0.00000 | 1.96056 | 7.0E-08 | SLC38A2 |
| 0.00009 | 1.96393 | 1.8E-03 | CTBP2 |
| 0.00000 | 1.96527 | 5.6E-05 | AGL |
| 0.00029 | 1.96757 | 4.6E-03 | PSMA3 |
| 0.00000 | 1.97249 | 3.6E-06 | TMEM123 |
| 0.00070 | 1.97278 | 9.1E-03 | PTX3 |
| 0.00000 | 1.97538 | 2.0E-05 | CSGALNACT2 |
| 0.00000 | 1.97685 | 2.8E-06 | ITSN2 |
| 0.00006 | 1.98011 | 1.3E-03 | SECISBP2L |
| 0.00006 | 1.98290 | 1.4E-03 | MME |
| 0.00000 | 1.99740 | 1.0E-04 | P2RY14 |
| 0.00003 | 1.99839 | 7.1E-04 | PTGS2 |
| 0.00001 | 1.99878 | 2.4E-04 | APIP |
| 0.00000 | 1.99939 | 1.0E-04 | IGJ |
| 0.00007 | 2.00846 | 1.6E-03 | APPL1 |
| 0.00042 | 2.01271 | 6.1E-03 | CASP8AP2 |
| 0.00004 | 2.01364 | 9.9E-04 | PDE6D |
| 0.00002 | 2.01930 | 5.6E-04 | EMC2 |
| 0.00011 | 2.02187 | 2.2E-03 | CHMP5 |
| 0.00000 | 2.02374 | 2.7E-07 | LBR |
| 0.00000 | 2.02420 | 1.4E-04 | MEX3C |
| 0.00000 | 2.02842 | 4.9E-05 | VCAN |
| 0.00005 | 2.02875 | 1.1E-03 | LOC145783 |
| 0.00000 | 2.03367 | 2.7E-06 | KIF2A |
| 0.00000 | 2.03374 | 3.1E-06 | CBR4 |
| 0.00007 | 2.03420 | 1.6E-03 | CKS2 |
| 0.00003 | 2.03445 | 7.6E-04 | PCMTD2 |
| 0.00000 | 2.04417 | 5.8E-05 | RALGAPA1 |
| 0.00007 | 2.04627 | 1.5E-03 | LOC100996668 |
| 0.00000 | 2.05224 | 6.8E-05 | RPS3A |
| 0.00000 | 2.05967 | 2.1E-06 | PELI1 |
| 0.00000 | 2.06085 | 7.7E-06 | KRCC1 |
| 0.00000 | 2.06146 | 5.6E-05 | ACAP2 |
| 0.00010 | 2.06373 | 2.1E-03 | ITGA4 |
| 0.00000 | 2.06884 | 1.1E-04 | PDZD8 |
| 0.00007 | 2.07019 | 1.5E-03 | DENND4A |
| 0.00000 | 2.07110 | 9.3E-06 | UBA3 |
| 0.00015 | 2.07273 | 2.9E-03 | NUPL1 |
| 0.00038 | 2.07993 | 5.7E-03 | ACN9 |
| 0.00000 | 2.09120 | 9.6E-06 | PPP1CB |
| 0.00000 | 2.09381 | 1.9E-05 | GNG10 |
| 0.00077 | 2.09401 | 9.8E-03 | YIPF4 |
| 0.00003 | 2.09464 | 8.2E-04 | SLMO2 |
| 0.00000 | 2.09606 | 1.6E-05 | MST4 |
| 0.00037 | 2.09917 | 5.5E-03 | ACTR6 |
| 0.00046 | 2.10527 | 6.5E-03 | BBS10 |
| 0.00000 | 2.11743 | 4.7E-08 | SMCHD1 |
| 0.00001 | 2.11747 | 3.0E-04 | DDX50 |
| 0.00000 | 2.11902 | 1.9E-06 | FNDC3A |
| 0.00001 | 2.12141 | 4.3E-04 | CD2AP |
| 0.00015 | 2.12309 | 2.7E-03 | C1D |
| 0.00000 | 2.13814 | 1.7E-04 | BUB3 |
| 0.00000 | 2.15143 | 4.1E-05 | IFRD1 |
| 0.00001 | 2.15658 | 2.4E-04 | ERBB2IP |
| 0.00009 | 2.16396 | 1.9E-03 | STK3 |
| 0.00032 | 2.16403 | 4.9E-03 | CDC73 |
| 0.00001 | 2.16739 | 4.0E-04 | ERGIC2 |
| 0.00012 | 2.17011 | 2.4E-03 | CDC14A |
| 0.00000 | 2.17164 | 9.7E-08 | NAMPT |
| 0.00001 | 2.18231 | 2.5E-04 | MIR1304 |
| 0.00000 | 2.18474 | 6.7E-05 | NRIP1 |
| 0.00000 | 2.18688 | 7.2E-05 | RNF138 |
| 0.00000 | 2.18962 | 2.6E-05 | RPL9 |
| 0.00000 | 2.19134 | 3.7E-05 | SRSF11 |
| 0.00002 | 2.19202 | 4.8E-04 | CXCL8 |
| 0.00001 | 2.19724 | 3.6E-04 | SLC35A5 |
| 0.00000 | 2.19799 | 4.9E-05 | MBNL1 |
| 0.00001 | 2.20650 | 4.0E-04 | TWF1 |
| 0.00000 | 2.20677 | 1.6E-04 | AIMP1 |
| 0.00006 | 2.21160 | 1.3E-03 | ACTG1P4 |
| 0.00015 | 2.21804 | 2.9E-03 | SRSF10 |
| 0.00006 | 2.21830 | 1.3E-03 | HAT1 |
| 0.00000 | 2.22323 | 6.8E-05 | CLDND1 |
| 0.00000 | 2.22324 | 1.6E-04 | CCP110 |
| 0.00000 | 2.22587 | 3.0E-07 | SLC35A1 |
| 0.00000 | 2.23221 | 2.3E-06 | MICU2 |
| 0.00000 | 2.23841 | 5.0E-07 | ANKRD49 |
| 0.00018 | 2.24062 | 3.2E-03 | ZNF518A |
| 0.00002 | 2.25228 | 5.6E-04 | COMMD8 |
| 0.00000 | 2.25449 | 1.5E-04 | EID1 |
| 0.00001 | 2.25843 | 2.2E-04 | SNX10 |
| 0.00000 | 2.26295 | 2.2E-05 | KIAA1109 |
| 0.00006 | 2.27094 | 1.3E-03 | NOL8 |
| 0.00000 | 2.27703 | 1.1E-05 | CMPK1 |
| 0.00001 | 2.29407 | 3.6E-04 | RNF6 |
| 0.00000 | 2.29623 | 1.6E-04 | CLEC2B |
| 0.00001 | 2.30053 | 4.5E-04 | MTHFD2 |
| 0.00000 | 2.30224 | 3.9E-06 | SACM1L |
| 0.00000 | 2.30593 | 1.9E-04 | TMEM165 |
| 0.00000 | 2.31155 | 9.1E-06 | COPS2 |
| 0.00008 | 2.32122 | 1.7E-03 | OXR1 |
| 0.00018 | 2.32871 | 3.2E-03 | IRAK3 |
| 0.00000 | 2.34565 | 1.1E-04 | ZFYVE16 |
| 0.00001 | 2.35776 | 3.0E-04 | SLC25A24 |
| 0.00016 | 2.35777 | 2.9E-03 | NDUFA4 |
| 0.00000 | 2.35991 | 3.7E-05 | DEK |
| 0.00000 | 2.38226 | 9.1E-05 | RBM15 |
| 0.00003 | 2.38532 | 7.4E-04 | TVP23B |
| 0.00000 | 2.38724 | 4.2E-05 | CEP135 |
| 0.00000 | 2.39889 | 1.6E-06 | PCNP |
| 0.00000 | 2.39969 | 9.7E-08 | NEK7 |
| 0.00001 | 2.40580 | 3.2E-04 | BMI1 |
| 0.00000 | 2.40806 | 1.0E-04 | HINT1 |
| 0.00000 | 2.41253 | 3.6E-06 | CASP3 |
| 0.00009 | 2.43120 | 1.8E-03 | PLSCR1 |
| 0.00005 | 2.43235 | 1.3E-03 | ZNF83 |
| 0.00008 | 2.44816 | 1.8E-03 | MAP4K3 |
| 0.00000 | 2.46365 | 1.9E-06 | ARID4A |
| 0.00002 | 2.48439 | 5.0E-04 | SMARCA5 |
| 0.00003 | 2.48619 | 7.6E-04 | LTN1 |
| 0.00000 | 2.48838 | 7.2E-05 | C6orf211 |
| 0.00004 | 2.49558 | 9.3E-04 | CCPG1 |
| 0.00000 | 2.49792 | 3.8E-08 | ABHD3 |
| 0.00000 | 2.52579 | 1.8E-06 | MYBL1 |
| 0.00000 | 2.52980 | 4.7E-07 | GOLGA8N |
| 0.00001 | 2.53490 | 2.7E-04 | COX7B |
| 0.00000 | 2.53540 | 1.8E-05 | PPIP5K2 |
| 0.00001 | 2.54483 | 3.9E-04 | BAZ2B |
| 0.00000 | 2.54642 | 5.8E-06 | ARGLU1 |
| 0.00003 | 2.58343 | 8.4E-04 | RAP2C |
| 0.00000 | 2.59923 | 6.2E-05 | TRMT13 |
| 0.00000 | 2.61557 | 6.7E-05 | SH2D1A |
| 0.00000 | 2.62412 | 4.3E-07 | EVI2A |
| 0.00000 | 2.63502 | 2.1E-06 | MBNL2 |
| 0.00000 | 2.63544 | 1.9E-06 | C2CD5 |
| 0.00001 | 2.65078 | 2.9E-04 | AHR |
| 0.00002 | 2.66264 | 5.5E-04 | ACSL4 |
| 0.00001 | 2.67964 | 2.3E-04 | HSPE1-MOB4 |
| 0.00000 | 2.71685 | 2.4E-06 | TMED7 |
| 0.00000 | 2.72229 | 1.0E-04 | ASF1A |
| 0.00000 | 2.72386 | 7.0E-05 | RB1 |
| 0.00002 | 2.74702 | 6.1E-04 | DMXL1 |
| 0.00000 | 2.75348 | 1.6E-05 | FAM188A |
| 0.00000 | 2.78852 | 7.7E-06 | SP3 |
| 0.00000 | 2.79111 | 6.8E-06 | CHMP2B |
| 0.00000 | 2.85383 | 5.4E-06 | PSMC6 |
| 0.00000 | 2.85546 | 5.3E-06 | TMEM168 |
| 0.00000 | 2.89005 | 4.5E-06 | MOB1A |
| 0.00000 | 2.90495 | 3.5E-06 | PHTF2 |
| 0.00000 | 2.92840 | 1.8E-06 | ZDHHC17 |
| 0.00000 | 2.96496 | 1.8E-04 | CLK1 |
| 0.00000 | 2.97329 | 1.6E-05 | ZNF107 |
| 0.00000 | 3.09717 | 9.5E-05 | UFL1 |
| 0.00000 | 3.13302 | 1.3E-09 | ANKRD12 |
| 0.00000 | 3.31699 | 9.7E-08 | C12orf29 |
| 0.00000 | 3.33968 | 6.1E-07 | PIKFYVE |
| 0.00000 | 3.34074 | 2.1E-05 | ATF1 |
| 0.00000 | 3.55259 | 1.6E-07 | ZNF267 |
